# Combined analysis of keratinocyte cancers identifies novel genome-wide loci

**DOI:** 10.1093/hmg/ddz121

**Published:** 2019-06-07

**Authors:** Upekha E Liyanage, Matthew H Law, Xikun Han, Jiyuan An, Jue-Sheng Ong, Puya Gharahkhani, Scott Gordon, Rachel E Neale, Catherine M Olsen, 23andMe Research Team, Stuart MacGregor, David C Whiteman

**Affiliations:** 1 Statistical Genetics Lab, QIMR Berghofer Medical Research Institute, 300 Herston Road, Brisbane, QLD 4006, Australia; 2 Genetic Epidemiology, QIMR Berghofer Medical Research Institute, 300 Herston Road, Brisbane, QLD 4006, Australia; 3 Cancer Aetiology and Prevention, QIMR Berghofer Medical Research Institute, 300 Herston Road, Brisbane, QLD 4006, Australia; 4 Cancer Control Group, QIMR Berghofer Medical Research Institute, 300 Herston Road, Brisbane, QLD 4006, Australia; 5 Faculty of Medicine, The University of Queensland, Brisbane, QLD 4006, Australia

## Abstract

The keratinocyte cancers (KC), basal cell carcinoma (BCC) and squamous cell carcinoma (SCC) are the most common cancers in fair-skinned people. KC treatment represents the second highest cancer healthcare expenditure in Australia. Increasing our understanding of the genetic architecture of KC may provide new avenues for prevention and treatment. We first conducted a series of genome-wide association studies (GWAS) of KC across three European ancestry datasets from Australia, Europe and USA, and used linkage disequilibrium (LD) Score regression (LDSC) to estimate their pairwise genetic correlations. We employed a multiple-trait approach to map genes across the combined set of KC GWAS (total *N* = 47 742 cases, 634 413 controls). We also performed meta-analyses of BCC and SCC separately to identify trait specific loci. We found substantial genetic correlations (generally 0.5–1) between BCC and SCC suggesting overlapping genetic risk variants. The multiple trait combined KC GWAS identified 63 independent genome-wide significant loci, 29 of which were novel. Individual separate meta-analyses of BCC and SCC identified an additional 13 novel loci not found in the combined KC analysis. Three new loci were implicated using gene-based tests. New loci included common variants in *BRCA2* (distinct to known rare high penetrance cancer risk variants), and in *CTLA4,* a target of immunotherapy in melanoma. We found shared and trait specific genetic contributions to BCC and SCC. Considering both, we identified a total of 79 independent risk loci, 45 of which are novel.

## Introduction

Basal cell carcinoma (BCC) and squamous cell carcinoma (SCC) are the most common cancers of the skin. Together, they are known as keratinocyte carcinomas (KC), so named because the cells of origin are the abundant, keratin-producing cells of the epidermis. BCC and SCC both arise in the epidermis but are histologically and clinically distinct from each other (Supplementary Material, Fig. S1) (1). BCC and SCC tend to be slow growing skin cancers when compared with melanoma, which is the most aggressive form (2). SCC has a lower prevalence than BCC, but accounts for the most KC-related deaths (2). While the principal treatment modalities for KC (surgery, curettage and destruction) are highly effective in the majority of cases, they cause pain and scarring, carry risks of infection and represent the second highest expenditure on cancer healthcare in Australia (3,4). In the USA the average annual cost of skin cancer (melanoma and keratinocyte cancers) treatment was $8.1 billion, with ~4.9 million patients treated for skin cancer from 2007 to 2011 (5).

BCC and SCC share the major risk factors of ultraviolet radiation (UVR) exposure and fair skin, and as such occur most commonly on sun exposed sites (6). Consequently, KC are extremely frequent in fair-skinned populations living in areas with high UVR such as Australia and the southern United States (2). The age-standardized incidence rate in Australia for BCC is 770 per 100 000 person years; for SCC it is 270 per 100 000 person years (7). In addition to the role of UVR, immunosuppression increases the risk of both SCC and BCC, reinforcing the importance of the immune system in early elimination of cancerous cells (6).

Both skin cancer and its established risk factors, such as pigmentation, have a heritable component. A recent large twin study estimated the heritability of keratinocyte cancer to be 43% [95% confidence interval (CI), 26–59%] (8). Previous genome-wide association studies (GWAS) and related approaches have identified 29 loci associated with BCC susceptibility and 11 loci associated with SCC (9–14). Seven of these loci are associated with both BCC and SCC, suggesting shared genetic pathways in keratinocyte cancer etiology. Some of these common single nucleotide polymorphisms (SNPs) have a functional role in pigmentation (e.g. *MC1R, IRF4, TYR*) and freckling tendency (*BNC2*) (10,12), consistent with the strong causal role of UVR in keratinocyte cancer risk. Identifying additional risk loci may uncover novel genetic pathways underpinning cancer risk and identify new drug targets to assist in the development of future cancer therapies (15).

We first conducted a series of GWAS across four large datasets of ethnically homogenous ancestry (White British/European) from Australia, Europe and the USA. In addition to performing GWAS of BCC and SCC cases, where possible we also analysed a single case group combining all KC cases. For the QSkin and electronic medical records for genetic research (eMERGE) GWAS datasets, while histology records confirmed the occurrence of a KC, a specific diagnosis of BCC or SCC was not available for all participants (Methods). For these two cohorts all cases were included in the KC phenotype.

Given the overlap in the risk loci identified to date in BCC and SCC (e.g. *BNC2, MC1R, ASIP*) (11,12), it is likely these traits have an overlapping genetic basis. To explore this hypothesis, we tested for genetic correlation between the BCC, SCC and the KC phenotype GWAS using linkage disequilibrium (LD) score regression (LDSC) (16). Finding a high degree of genetic correlation across the KC phenotypes, we performed a combined KC analysis bringing together the BCC, SCC and KC GWAS using multiple trait analysis of GWAS (MTAG), a method that accounts for incomplete genetic correlation across traits as well as sample overlap (17). As the genetic correlations across BCC, SCC and the KC phenotype were high but not complete, there are likely genetic variants that contribute to either BCC or SCC specifically. We explored this by performing individual separate meta-analyses of BCC and SCC alone.

## Results

### Genetic correlations across BCC, SCC and KC

**Figure 1:**
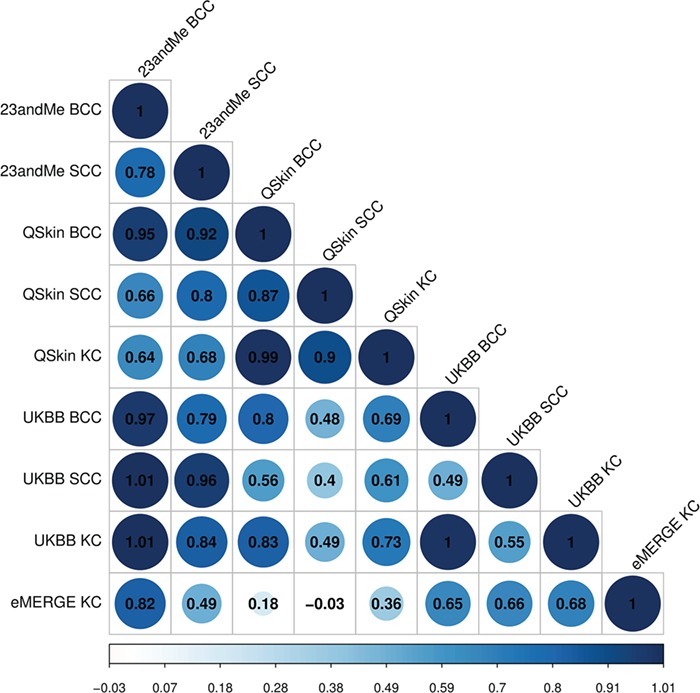
Genetic correlation between BCC, SCC and KC cancers. With the exception of the comparisons between the smallest datasets, all correlations values are significant at *P*-value <0.05 (eMERGE KC versus QSkin BCC *P*-value = 0.37; eMERGE KC versus QSkin SCC *P*-value = 0.88; full results in Supplementary Material, Table S1).

For each of the contributing GWAS there was little to no evidence for inflation due to population stratification (Supplementary Material, Table S1). Using LDSC we found moderate or high genetic correlations between SCC, BCC and combined KC phenotypes (Fig. 1, Supplementary Material, Table S1). For example, UK Biobank (UKBB) BCC GWAS versus the 23andMe SCC GWAS Rg = 0.79 (CI 0.54–1.03, *P*-value = 2.6 × 10^−10^), while the QSkin BCC GWAS versus the UKBB SCC GWAS was Rg = 0.56 (CI 0.11–1.00, *P*-value = 0.01). These correlations indicated that a multivariate analysis using MTAG was likely to be more powerful for identifying loci than separate analyses of BCC or SCC. Moreover, the generally high correlations justified including the ~8000 additional KC cases for which type-specific histology (i.e. BCC or SCC) was not available (Table 1).

**Table 1:** Sample sizes used for GWAS analysis

	**BCC**		**SCC**		**KC**	
**Study sample**	**Cases**	**Controls**	**Cases**	**Controls**	**Cases**	**Controls**
**QSkin**	1995	4797^a^	821	4797^a^	8145	4797^a^
**UKBB**	16 847	340 302^a^	2274	340 302^a^	18 538	340 302^a^
**eMERGE KC**	-	-	-	-	1565	8756
**23andMe**	12 945	274 252^a^	6579	280 558^a^	19524^b^	280 558^b^
**Total**	31 787	619 351	9674	625 657	47 742	634 413

^a^Control sets for QSkin, 23andMe and UKBB GWAS are overlapping. ^b^Combined KC analysis GWAS set for 23andMe reported to illustrate effective sample size for the combined KC analysis using MTAG (which accounts for sample overlap); only individual BCC and SCC GWAS were available for 23andMe.

### Combined multi-trait analysis of KC

Following the multi-trait analysis of the UKBB KC GWAS, 23andMe BCC, 23andMe SCC, QSkin KC and eMERGE KC GWAS (Table 1), the adjusted output for the UKBB KC GWAS identified 78 independent genome-wide significant SNPs assigned to 63 to risk loci, 29 of which are novel (Table 2, complete results for all independent SNPs can found in Supplementary Material, Table S2).

**Table 2:** Novel KC loci identified using MTAG

**CHR**	**BP**	**SNP**	**EA/NEA**	***P***	**OR [95% CI]**	**Nearest protein coding gene**
1	110 724 488	rs535930	G/A	2.08 × 10^−8^	0.95 [0.94–0.97]	*KCNC4*
1	114 303 808	rs6679677	C/A	6.50 × 10^−11^	1.10 [1.07–1.13]	*PHTF1*
1	154 984 363	rs1870940	G/A	7.88 × 10^−11^	0.94 [0.92–0.96]	*ZBTB7B*
1	242 023 898	rs4149909	G/A	2.35 × 10^−9^	1.16 [1.10–1.21]	*EXO1*
2	5 684 786	rs62112661	C/A	4.40 × 10^−8^	0.95 [0.94–0.97]	*SOX11*
2	7 704 860	rs79522206	G/A	4.38 × 10^−16^	0.81 [0.77–0.85]	*RNF144A*
2	37 189 296	rs3845780	T/C	1.85 × 10^−9^	0.95 [0.94–0.97]	*STRN*
2	38 298 139	rs1800440	T/C	4.50 × 10^−12^	1.08 [1.06–1.10]	*CYP1B1*
2	88 554 351	rs6707137	G/A	5.80 × 10^−22^	1.19 [1.15–1.23]	*THNSL2*
2	204 734 487	rs231779	T/C	9.66 × 10^−15^	0.94 [0.92–0.95]	*CTLA4*
3	98 447 140	rs7620634	G/A	1.82 × 10^−8^	1.06 [1.04–1.08]	*ST3GAL6*
5	149 192 846	rs17110447	G/A	1.66 × 10^−9^	0.94 [0.93–0.96]	*PPARGC1B*
6	4 979 956	rs1246946	T/C	5.32 × 10^−15^	0.94 [0.92–0.95]	*RPP40*
6	90 976 768	rs72928038	G/A	1.24 × 10^−20^	1.11 [1.09–1.14]	*BACH2*
7	6 418 673	rs836489	T/G	1.23 × 10^−8^	0.95 [0.94–0.97]	*RAC1*
7	29 132 279	rs117744081	G/A	2.07 × 10^−19^	0.79 [0.75–0.83]	*CPVL*
7	50 176 163	rs10228836	G/A	1.70 × 10^−10^	0.95 [0.93–0.96]	*ZPBP*
8	98 367 884	rs4735451	T/C	4.75 × 10^−9^	1.06 [1.04–1.08]	*TSPYL5*
8	120 082 971	rs13261635	T/C	1.43 × 10^−8^	0.95 [0.93–0.97]	*COLEC10*
9	106 858 192	rs3739737	G/A	3.55 × 10^−8^	1.05 [1.03–1.07]	*SMC2*
10	10 771 564	rs12767525	T/C	5.49 × 10^−9^	1.05 [1.04–1.07]	*CELF2*
10	64 402 433	rs10995255	G/A	6.51 × 10^−11^	0.94 [0.93–0.96]	*ZNF365*
11	64 107 735	rs663743	G/A	2.35 × 10^−9^	0.95 [0.93–0.97]	*CCDC88B*
11	65 580 638	rs12576996	T/G	3.77 × 10^−11^	1.07 [1.05–1.09]	*OVOL1*
12	96 379 806	rs3213737	G/A	1.11 × 10^−8^	1.05 [1.03–1.07]	*HAL*
13	32 954 561	rs7328654	G/A	5.82 × 10^−10^	1.05 [1.04–1.07]	*N4BP2L1*
14	75 935 908	rs77100309	G/A	1.65 × 10^−8^	0.91 [0.89–0.94]	*JDP2*
19	1 106 845	rs2075710	T/C	1.68 × 10^−8^	1.06 [1.04–1.08]	*GPX4*
20	49 399 007	rs62202837	T/C	2.23 × 10^−13^	0.93 [0.91–0.95]	*BCAS4*

Hg19 chromosome (**CHR**) and base pair (**BP**) positions are provided for each SNP. Odds ratios (**OR**) and ***P***-values are from the MTAG analysis of five GWAS datasets; UKBB KC, eMERGE KC, 23andMe BCC, 23andMe SCC and QSkin KC (sample sizes reported in Table 1). The OR and 95% confidence interval is for the effect allele (**EA**); the non-effect allele (**NEA**) is also reported. We also report the **nearest protein coding gene** for each SNP; more complete gene annotation data can be found on Supplementary Material, Table S2.

A number of the newly identified KC SNPs are expression quantitative trait loci (eQTLs) for nearby genes, or are associated with other traits (Table 3; results for all SNP can be found in Supplementary Material, Table 2). Two of the novel KC loci highlight genes that are existing drug targets (Supplementary Material, Table S2). The first, rs231779 on chromosome 2, is an eQTL for *CTLA4*, which is a target for immunotherapy medications including ipilimumab and tremelimumab, which are already in use for melanoma. The second, rs12576996 on chromosome 11 is an eQTL for *MAP 3K11*, which has been investigated as a drug target for Parkinson’s disease using CEP-1347.

**Table 3:** Annotation of novel KC loci

**CHR**	**BP**	**SNP**	**eQTL skin**	**eQTL whole blood**	**eQTL other tissue**	**PheWAS summary**
1	110 724 488	rs535930	-	-	-	Educational level
1	114 303 808	rs6679677	-	-	-	Hyper/hypothyroidism, medications for cholesterol, blood pressure, T1D, RA
1	154 984 363	rs1870940	*ZBTB7B, ADAM15*	-	*ZBTB7B, ADAM15, DCST2, RP11-307C12.11*	Anthropometric measures, BMI, BMR
1	242 023 898	rs4149909	-	-	-	-
2	5 684 786	rs62112661	-	-	-	-
2	7 704 860	rs79522206	-	-	-	-
2	37 189 296	rs3845780	-	*STRN*	*GPATCH11, STRN*	Leg fat percentage
2	38 298 139	rs1800440	-	-	-	-
2	88 554 351	rs6707137	-	-	-	-
2	204 734 487	rs231779	-	-	*CTLA4*	Hyper/hypothyroidism
3	98 447 140	rs7620634	*DCBLD2*, *ST3GAL6-AS1*	-	*ST3GAL6, ST3GAL6-AS1, PDLIM1P4*	-
5	149 192 846	rs17110447	-	-	-	-
6	4 979 956	rs1246946	-	-	*RPP40, RP11-428J1.4, RP11-428J1.5*	-
6	90 976 768	rs72928038	-	-	-	Asthma, T1D, hypothyroidism
7	6 418 673	rs836489	*DAGLB*	*DAGLB, FAM220A*	*RAC1, DAGLB, FAM220A*	Anthropometric measures
7	29 132 279	rs117744081	-	-	*CTB-113D17.1*	-
7	50 176 163	rs10228836	-	-	*C7orf72*	-
8	98 367 884	rs4735451	-	-	-	-
8	120 082 971	rs13261635	-	-	*MAL2*, *NOV*, *COLEC10*	Height
9	106 858 192	rs3739737	-	*SMC2*	*SMC2, RP11-82L2.1*	-
10	10 771 564	rs12767525	-	-	-	-
10	64 402 433	rs10995255	-	*ADO*	*ADO*	-
11	64 107 735	rs663743	*CCDC88B, PPP1R14B*	*AP003774.1*	*AP003774.1, CCDC88B, PPP1R14B, PLCB3*	-
11	65 580 638	rs12576996	*CTSW, EIF1AD, KRT8P26, MAP 3K11*	*MAP 3K11*	*BANF1, CTSW, EFEMP2, EIF1AD, FIBP, KRT8P26, MAP 3K11, NEAT1, OVOL1, SNX32*	Asthma, allergy, eczema, arm impedance
12	96 379 806	rs3213737	*HAL*, *RP11-256L6.3*		*AMDHD1*, *RP11-256L6.3*	Tanning
13	32 954 561	rs7328654	-	-	-	-
14	75 935 908	rs77100309	-	-	-	-
19	1 106 845	rs2075710	*GPX4*	-	*GPX4*	-
20	49 399 007	rs62202837				KC^*^

Hg19 chromosome (**CHR**) and base pair (**BP**) positions are provided for each SNP. Only genes reaching the significance threshold of 2 × 10^−6^, or for PheWAS traits reaching 5 × 10^−8^ are shown. KC, keratinocyte cancer; BMI, body mass index; BMR, basal metabolic rate; T1D, type 1 diabetes; RA, rheumatoid arthritis. Full details are provided in Supplementary Material, Table S2. ^*^Associated with KC at genome-wide significance in the UKBB alone.

### Meta-analysis of BCC and SCC GWAS

After conducting the overall multi-trait KC GWAS, we performed individual meta-analysis of BCC and SCC using a fixed effects meta-analysis to identify any regions reaching genome-wide significance for a specific type of KC (Supplementary Material, Table S3). Seventy three loci were associated with either BCC or SCC, and the majority overlapped (Materials and Methods) with the MTAG KC analyses. A single locus previously reported for each of BCC and SCC reached genome-wide significance in the individual analyses but not in the KC MTAG analysis (rs9419958/*OBFC1* and rs13301660/*SEC16A*, respectively, Supplementary Material, Table S3). We further identified 13 new loci by conducting BCC or SCC GWAS meta-analysis; these loci did not reach genome-wide significance in our KC MTAG analyses and previous studies (Tables 4 and 5; full results including annotation can be found in Supplementary Material, Table S3). The chromosome 2 locus with peak SNP rs7563677 is an eQTL for *ITGB6*, representing a potentially novel drug target for KC. ITGB6 is a target of intetumumab currently in use for the treatment of prostate adenocarcinoma; additional drugs in use for this gene’s product are abituzumab for metastatic colorectal cancer, and STX-100 for idiopathic pulmonary fibrosis and melanoma Supplementary Material, Table S3.

**Table 4:** Novel loci from meta-analysis of BCC or SCC GWAS

**CHR**	**BP**	**SNP**	**EA/NEA**	**P**	**OR [95% CI]**	**Nearest protein coding gene**
2	11 526 716	rs12466910	A/G	2.97 × 10^−8^	0.95 [0.94–0.97]	*ROCK2*
2	161 356 717	rs7563677	C/G	4.58 × 10^−8^	0.93 [0.91–0.96]	*RBMS1*
5	44 412 065	rs11741260	A/G	8.74 × 10^−10^	1.07 [1.05–1.10]	*FGF10*
5	67 751 221	rs42905	A/C	2.82 × 10^−8^	1.05 [1.03–1.06]	*PIK3R1*
6	15 535 321	rs9383064^a^	C/G	1.29 × 10^−8^	1.10 [1.06–1.14]	*DTNBP1*
6	150 353 556	rs12205199	A/C	1.13 × 10^−8^	1.05 [1.03–1.07]	*RAET1L*
8	116 632 819	rs2721936	A/T	2.06 × 10^−9^	1.05 [1.03–1.07]	*TRPS1*
9	681 645	rs9408674	A/G	2.88 × 10^−9^	1.05 [1.03–1.07]	*KANK1*
9	19 059 865	rs60269255	C/G	1.94 × 10^−8^	1.07 [1.04–1.09]	*HAUS6*
10	13 740 917	rs1887004	T/C	6.65 × 10^−10^	0.95 [0.93–0.96]	*RP11-295P9.3*
15	79 237 293	rs2289702	T/C	2.57 × 10^−9^	1.09 [1.06–1.11]	*CTSH*
19	50 151 686	rs7508601	A/T	3.23 × 10^−8^	0.95 [0.94–0.97]	*SCAF1*
20	37 746 454	rs209901	T/G	2.84× 10^−8^	0.95[0.93–0.97]	*DHX35*

a **CHR,** chromosome; **BP,** base pair position; **SNP**, single nucleotide polymorphism; **EA,** effect allele; **NEA**, non-effect allele; P, *P*-value, **OR [95% CI],** odds ratio, 95% confidence interval. ^**a**^The chromosome 6 region with peak SNP rs9383064 is associated with SCC; all other tabulated regions are associated BCC. We reported the **nearest protein coding gene**; for more detailed gene annotation see Supplementary Material, Table S3.

**Table 5:** Annotation of novel loci from meta-analysis of BCC or SCC GWAS

**CHR**	**BP**	**SNP**	**eQTL skin**	**eQTL whole blood**	**eQTL other tissue**	**PheWAS**
2	11 526 716	rs12466910	-	-	*LINC00570*	-
2	161 356 717	rs7563677	-	-	*ITGB6*	-
5	44 412 065	rs11741260	-	-	-	-
5	67 751 221	rs42905	-	-	-	-
6	15 535 321	rs9383064^a^	-	-	*DTNBP1*	-
6	150 353 556	rs12205199	-	-	-	Alopecia areata
8	116 632 819	rs2721936	-	-	-	Anthropometric measures
9	681 645	rs9408674	-	-	*KANK1*	-
9	19 059 865	rs60269255	-	-	*HAUS6*	-
10	13 740 917	rs1887004	-	-	-	-
15	79 237 293	rs2289702	*CTSH*	*CTSH*	*CTSH*	Lung cancer, T1D
19	50 151 686	rs7508601	-	-	-	Hypothyroidism/myxoedema, vitiligo
20	37 746 454	rs209901	-	-	-	-

a **CHR,** chromosome; **BP,** base pair position; **SNP**, single nucleotide polymorphism; **T1D**, type 1 diabetes mellitus. ^**a**^The chromosome 6 region with peak SNP rs9383064 is associated with SCC; all other tabulated regions are associated BCC. Full details are provided in Supplementary Material, Table S3.

### Heritability of BCC and SCC

h^2^_SNP_ (SNP-heritability) estimates for BCC and SCC were 13.1% (95% CI = 9.7–16.5%) and 6.8% (95% CI = 0.9–12.7%), respectively. Of that, GWAS significant loci contributed ~5.9% to BCC h^2^_SNP_ and 2.7% for SCC.

### Consistency of effect sizes across traits

Having identified loci associated with both the KC phenotype, and with BCC and SCC alone, we compared the effect size for the lead SNP from each of the independent loci identified across the three datasets (Methods; Supplementary Material, Table S8).

Most of the SNPs identified in the KC MTAG analysis including rs231779 *(CTLA4)*, rs7328654 *(BRCA2)* and rs3213737 *(HAL)* had consistent effect estimates in the BCC and SCC meta-analysis. However, we noted that when SNPs differed they tended to have larger effect sizes for BCC than SCC, particularly for rs79522206 *(RNU6ATAC37P)* and rs6707137 *(RNY4P15).* This may be due to the effect of winner’s curse bias as the majority of our meta-analysis and MTAG sample set was comprised of BCC GWAS (47). However, some variants have larger effects in the SCC meta-analysis including in genes involved in pigmentation e.g. rs1805007 *(MC1R)*, rs1805008 *(TUBB3)* and rs12203592 *(IRF4)* (11,14). Similar magnitude of effect estimates for BCC and SCC was noted for other pigmentation variants, including rs1126809 (*TYR*) and rs35407 (*SLC45A2*) (11). Some of the previously known variants associated with BCC or SCC but not associated with pigmentation pathway particularly, rs2853677 *(TERT)* and rs78378222 *(TP53)* (48,49), showed significantly larger effect estimates on BCC risk than SCC risk.

### Gene-based approaches for KC

FastBAT gene-based test for KC revealed an additional three genes in two loci not identified in the single SNP analyses for KC, BCC or SCC (Methods; novel genes marked in bold in Supplementary Material, Table S4**)**. We also conducted a gene-set enrichment analysis using the KC GWAS results (Methods), which identified that there was significant enrichment for the gene-sets ‘T cell selection’ (*P*-value = 2.1 × 10^−7^, Bonferroni-corrected *P*-value = 0.002) and ‘keratin filament’ (*P*-value = 1.9 × 10^−6^, Bonferroni-corrected *P*-value = 0.02).

As a parallel approach we performed a transcriptome wide association study (TWAS), as implemented in MetaXcan, using two skin and a whole blood expression set from GTEx Supplementary Material, Tables S5, S6 and S7. This approach determines the genetically predicted change in gene expression due to variants associated with KC, and can predict the potential functional target gene at loci that reach significance (Materials and Methods) in the MTAG analysis. In addition TWAS can identify additional regions where imputed gene expression levels are associated with KC at significance but no single SNP reaches genome-wide significance. A number of imputed genes are associated with KC in regions identified in the single SNP MTAG results, and an additional gene on chromosome 10 is significantly associated with KC (*SLC35G1 P*-value = 1.69 × 10^−6^ sun-exposed skin, highlighted in bold in (Supplementary Material, Table S5). When considered together, the FastBAT and TWAS approaches highlight a total of four genes in three loci (*ASXL2, RABEPK, HSPA5, SLC35G1*) that are significant a *P*-value <0.05 corrected for the number of genes and are not identified in the single SNP analyses.

## Discussion

In this well-powered analysis of keratinocyte cancer, we have assembled large GWAS datasets examining BCC, SCC and, for the majority of cohorts, KC. We showed substantial genetic correlation between discrete GWAS of BCC, SCC and the KC phenotype (Fig. 1, Supplementary Material, Table S1), and leveraged this correlation in a multiple trait combined KC GWAS analysis to identify 83 independent genome-wide significant genetic variants in 63 loci (Table 2, Supplementary Materials, Tables S2 and S3). Thirty-four of these loci have been previously reported for BCC or SCC alone using a range of approaches (9–12,14), while 29 of them are novel (Supplementary Material, Table S2). Most of the variants affected both BCC and SCC, with a small subset affecting only one or the other of these cancers (Supplementary Material, Table S8). However, few loci, which were GWAS significant in previous studies, were not replicated in our study (e.g. rs192481803 (2p22.3), rs74899442 (11q23.3) for SCC (11) and rs78097823 (22q 12.1), rs1050529 (6p 21.33) for BCC (12). Some of these were rare SNPs (rs192481803, rs74899442) and were filtered out in MTAG analysis.

For the first time we found an SNP in the cytotoxic lymphocyte-associated antigen-4 (*CTLA4*) gene associated with KC risk (2q33.2, rs231779 OR = 0.94; 95% CI = 0.92–0.95, *P*-value = 9.7 × 10^−15^; Supplementary Material, Table S2). The importance of this finding is that the *CTLA4* protein plays a key role in activating the anti-tumour response against cancer cells (50). In addition, according to a previous animal study CTLA4 mediates the effects of UV-induced immunosuppression, which is particularly important in the development of skin cancer (51). Ipilimumab (antibodies targeting CTLA4) are currently in use to treat melanoma patients with stage III or IV disease (43,50). However, this particular SNP has not been identified in GWAS of melanoma (52,53). Identification of this SNP highlights the potential future avenue to use Ipilimumab for KC therapy.

SNP rs7563677 at 2q24.2 is an eQTL for *ITGB6*, representing a putatively novel drug target for KC, with an associated drug STX-100 currently in a Phase I clinical trial for melanoma. This SNP only reaches formal significance in the BCC analysis, although in practice this particular SNP fails quality control (QC) in some KC/SCC only datasets. However, the effect estimates of the proxy SNP, rs6736111 LD (r^2^ = 0.55) were similar across all traits suggesting that it is a KC SNP (see Supplementary Material, Table S8). Furthermore, the KC associated SNP rs12576996 (11q13.1) is an eQTL for a gene (*MAP 3K11*) that is a potential drug target (CEP-1347), which is currently in clinical trials for Parkinson’s disease, and our findings may support repurposing for KC.

One of the novel KC SNPs we identified, rs7328654, lies in an intron of *BRCA2* (13q13.1). *BRCA2* is a tumor suppressor gene; rare variants in this gene, including rs11571833, are associated with a dramatically increased risk of various cancers including breast, lung, ovarian and prostate (54–57). A recent study conducted by Rafnar *et al*. (58) identified that K3326* variant (*BRCA2*) is associated with SCC of the skin (OR = 1.69; 95% CI = 1.26–2.26, *P* = 4.2 × 10^−4^) However, similar effect estimates were noted (OR = 1.94; 95% CI = 1.10–3.38) for rs11571833 (A/T) with a *P*-value of 0.02 in our SCC meta-analysis. The common variant that we identified (rs7328654) is not in LD with the rare high penetrance variants (e.g. for rs11571833 r^2^ = 0.001; the other pathogenic *BRCA2* variants are also very rare and so cannot be in strong LD with our common variant). The common variant we identify (rs7328654) has not been reported for any cancer although it is associated with LDL cholesterol levels (59).

As expected, many of the KC risk variants are involved in pigmentation. rs6059655 (*ASIP*) in 20q11.22, rs1805007 (*MC1R*) in 16q24.3, rs12203592 (*IRF4*) in 6p25.3, rs1126809 (*TYR*) in 11q14.3 and rs16891982 (*SLC45A2*) in 5p15.33 are non-synonymous SNPs, or enhancers, which regulate gene expression. These SNPs have previously been found to be associated with SCC, BCC and melanoma risk (9–12,51–53,60–62). We found an association between rs3213737 (*HAL*, 12q23.1) and KC. This SNP was recently identified as associated with vitamin D levels (63) in the GeneATLAS database of UKBB data (64), and is genome-wide significantly associated with ease of skin tanning. This gene encodes an enzyme required to produce urocanic acid, which protects skin against UVR damage and UVR-induced immune suppression (65). A study conducted by Welsh *et al*. (66) did not find association for rs7297245 (*HAL*) with overall BCC or SCC risk; however, stratified analysis revealed that rs7297245 (A/A) (LD r^2^ = 0.2 with our lead SNP rs3213737) is associated with SCC in women alone. While we are unable to perform sex-stratified analyses, the T allele of rs7297245 [Haplotype reference consortium (HRC) reports for the other strand] is not associated with KC (UKBB MTAG output for rs7297245 *P* = 0.3) suggesting our signal is independent to the one reported by Welsh et al. Several of the implicated genes are involved in functions relating to immune response. Our gene-set enrichment analysis implicated the positive T-cell selection pathway. As previously reported for BCC and SCC, we identified associations in the major histocompatibility complex (MHC) region (rs9271611/*HLA-DQA16* in p21.32, and rs9277332/HLA-DPA1 in 6p21.32, rs2507999/HLA-B in 6p21.33) (67,68). In addition to the previously mentioned *CTLA4* locus, the new loci with peak SNPs rs6679677 and rs72928038 are associated with autoimmune-related traits (Table 3).

In addition to the multi-trait analysis of KC, we also performed individual separate meta-analyses of BCC and SCC, as the differing clinical outcomes and high but incomplete genetic correlation between SCC and BCC suggests that there may be both overlapping and discrete genetic risks for these conditions. While the majority of the loci overlapped with the KC analysis, there were a further 12 BCC and 1 SCC loci that did not overlap (Supplementary Material, Table S3). In terms of genes/loci common to both BCC and SCC, in addition to pigmentation genes (e.g. *MC1R*), overlapping pathways include immune response (e.g. rs2507999/*HLA-B* and rs231779/*CTLA4*) and telomere length (rs9419958/*OBFC1*) (Supplementary Material, Table S8). Associations of note specific to BCC include rs78378222 near *TP53*, which is critical for DNA repair and cell cycle control (69), and rs9383064 near *DTNBP1*, which is associated with SCC alone (Supplementary Material, Table S8).

Of note, identification of these novel loci associated with KC improves the predictability of polygenic risk scores, but marginally as the new variants have relatively smaller effect sizes.

### Strengths and limitations

Our study is the largest genetic study of KC to date and as a result we have more than doubled the number of significant loci. One major advantage of using MTAG is it allows the combination of genetically correlated traits, which would not necessarily be clinically similar traits. As we discuss above BCC and SCC are two different skin cancers with an overlapping risk factors and genetic susceptibility, and we used a range of approaches to identify overlapping and unique genetic risks.

Our study comprised (multi-trait) meta-analyses of GWAS where each individual study included ancestry matched, ethnically homogeneous cases and controls, reducing the likelihood that our associations were due to differences in population substructure.

A limitation is that a proportion of our cases were derived from self-reported data. Since people may misreport the type of KC they have, this may make it difficult to accurately assess the true genetic correlations between KC subtypes. Histology reports were obtained only for a subset of QSkin participants; the remainder did have histologically-confirmed keratinocyte cancers but the type-specific histology was not available for analysis. For this latter group, we included them only in the combined KC analysis. 23andMe included self-reported data; however, a separate sub study confirmed the validity of using self-report of KC type, which had high sensitivity (93%) and specificity (99%) compared with medical records (11).

We did not perform anatomical site specific analysis of KC or the analysis to determine genetic basis of having a single, multiple or recurrent KC lesions because the relevant data was not uniformly available across the datasets. Another possible limitation is that we were unable to perform histological subtype-specific GWAS meta-analysis (e.g. limited to the micronodular BCC subtype) as we did not have the requisite data. Even though we implicitly assume that the genetic risk is similar in all subtypes in our analysis, we acknowledge that the genetic signals we identify may derive from common subtypes rather than the rarer because the majority of our samples will represent the common forms of BCC and SCC.

Our most powerful analysis leveraged the strong genetic correlation between the input traits to maximize power for identifying new loci using the MTAG software. MTAG makes the assumption that the genetic correlations across traits are relatively homogeneous across the genome; while this seems likely in our situation (the effect sizes for previously identified BCC and SCC loci are broadly similar, in keeping with their high genome-wide genetic correlation), this is difficult to test formally. While MTAG maximizes our power for KC discovery generally, as our numbers for SCC are much smaller than those for BCC there may be additional SCC loci that we failed to identify due to poor statistical power.

Finally although our analysis identified new genes (including potential novel drug targets for KC), further fine-mapping and molecular genetic characterization is essential to pinpoint the underlying causal variants and to reveal the biological functions of the peak SNPs. Further, while we report potential drug targets for genes we identified as associated with KC, these would need to be assessed through preclinical models and clinical trials to fully evaluate their efficacy, safety, benefits versus risks before repurposing.

## Conclusions

Keratinocyte carcinomas are the most numerous of the cancers in countries with populations primarily of European ancestry. While mortality is low, morbidity is considerable and they impose substantial burdens on health systems around the world. With our large GWAS meta-analysis, and by combining a range of approaches, we were able to identify 45 novel regions associated with skin cancer risk, providing new insights into disease etiology and putatively novel drug targets for KC. However, replication studies are warranted to confirm these novel genome-wide associations.

## Materials and Methods

### Study participants

#### QSkin

QSkin is a large prospective study on KCs; it comprises a cohort of 43 794 men and women aged 40–69 years (18). The aim of the study is to identify the environmental and genetic risk factors for skin cancer. At baseline in 2011, data on participants’ phenotype, lifestyle and exposures to environmental risks were collected using a self-administered questionnaire. KCs are not routinely registered in Australian cancer registries, so health administration data from Medicare were used to identify treatments for KCs for the period from date of consent though to June 30, 2014. Exact diagnoses of BCC and SCC were established through further linkage with pathology records. Medicare is Australia’s universal health insurance scheme with virtually 100% coverage. The eight Medicare item codes for billing claims for excision of histologically confirmed KC have very high concordance (~97%) with histopathologic diagnoses (19).

Genotyping of 17 965 participants using Illumina Global Screening Array (San Diego, CA, USA) was conducted in 2017, which included 8803 individuals undergoing excision for one or more KC during the follow-up period 2011–2014. Prior to GWAS QC (see methods). Pathology records were able to confirm a BCC diagnosis for 2066 of these individuals, and for 854 SCC. Pathology records confirming the specific type of KC was unavailable for the remaining cases, allowing their use only in the KC phenotype analyses. Controls were QSkin individuals who reported no history of actinic skin lesions prior to baseline, and for whom no KC excisions were observed during follow-up.

#### UKBB

The UKBB is a biorepository database, which has information on genetics and more than 2000 phenotypes acquired through self-report, hospital records and linkage to registries for about half a million participants in the UK (20). This large prospective cohort study data recruitment was conducted from 2006 to 2010, aiming to collect a wide range of physical, biological, lifestyle measures and clinical outcomes of these voluntary participants. Further, these data have been embedded in national electronic health records for further clarification, completeness and accuracy (20).

We used clinically and histopathologically defined ICD9 and ICD10 definitions to classify BCC and SCC cases (Table 1; for full details of selection of case see the Supplemental Methods). We selected the cancer-free controls on the basis of not having (a) a current or prior cancer registry history of cancer, or benign or *in situ* tumors, including C44, unspecified malignant neoplasms of skin; and (b) not having self-reported cancer at the time of enrollment of the study (21) (for final numbers see following sections). Genotyping was conducted using Affymetrix UK Biobank Axiom array (Santa Clara, CA, USA) and Affymetrix UK BiLEVE Axiom array (22).

#### eMERGE

eMERGE is a database that contains a collection of genetic and phenotypic data identified through electronic medical records in five participating groups in the USA (23). A more detailed description of this cohort is available via the dbGaP website (dbGaP ID phs000360) (24). Briefly, cases were those with a KC phenotype, derived from at least two reports of ICD9 code of 173–173.9, V10.83, 209.31–209.26 or 173.00–173.99. KC free controls were those without any report of ICD9 code 172–173.99. Prior to GWAS QC (see following sections) there was GWAS data available for 1666 KC cases and 9643 KC free controls. eMERGE KC cases and cancer-free controls were genotyped on the Illumina Human660W-Quad_v1_A array (San Diego, CA, USA) (24).

#### 23andMe

23andMe is a personal genetics company located in Mountain View, California (25). GWAS data were available for participants who self-reported a history of SCC or BCC (11,12). A separate, sub study has been performed to ascertain the validity of self-reported skin cancer status by Chahal *et al.* (11). In this, randomly selected patients from the Stanford outpatient clinics were given the same questionnaire as used by 23andMe and were followed up in medical records to check the concordance between self-report data and medical records. A high percentage of true positives and true negatives were identified (sensitivity, specificity: BCC 93% and 99%, SCC 92% and 98%, respectively) (11,12). Hence, these figures support the accuracy of the self-reported questionnaire used in 23andMe data. Genotyping was conducted using four different arrays (V1, V2, V3, V4), which included custom variants as well as content from the Illumina HumanHap550+ BeadChip, and Illumina OmniExpress+ BeadChip arrays (12).

### GWAS analyses

#### QSkin

We filtered out 189 387 SNPs from the Illumina Global Screening array (San Diego, CA, USA) array data if the GenTrain score was <0.6, Hardy–Weinberg equilibrium (HWE) *P*-value was <1 × 10^−6^ and/or minor allele frequency (MAF) was <1% using GenomeStudio and PLINK (v1.9) (26), leaving 496 695 SNPs. We excluded participants with >5% missing genotypes from the initial set of 17 965 QSkin participants (remaining *N* = 17 643).

Using the University of Michigan Imputation Server genotypes were phased by Eagle 2 (27) and imputed to the Haplotype reference consortium (HRC) version r1.1 (28) via minimac version 3 (29). Following imputation, identity by descent (IBD) as measured by PLINK v1.9 was used to drop one individual from pairs with >0.1875 PI_HAT scores (*N* = 400 individuals) from the analysis. Further, we identified and removed prior to analysis 378 individuals with ancestral principal component values more than six standard deviations (SD) away from European HapMap populations. In total, 776 individuals were removed as one IBD pair were also principal components analysis (PCA) outliers.

Of the 16 687 post QC samples there were 8145 individuals with any KC diagnosis confirmed by Australian Medicare records, of which 1995 had a histologically confirmed BCC diagnosis and 821 a histologically confirmed SCC diagnosis. There were also 4797 controls who were cancer free without a history of actinic lesions. Using the same control set we performed a BCC, SCC and a KC GWAS (Table 1).

SNPs with MAF >0.01 and imputation quality score >0.3 were analysed with PLINK 2.0, with the first 10 principal components (PC), age, age^2^_,_ sex, sex * age and sex * age^2^ fitted as covariates. Following this we used the univariate LDSC approach (30) to ensure that our GWAS results were not inflated due to the biases such as model misspecification, population stratification and cryptic relatedness. The LDSC intercept for the SCC, BCC and KC GWAS was 1.01–1.04 indicating the majority of apparent inflation (λ ~1.1) is polygenic signal (Supplementary Material, Table S1).

#### UKBB

A detailed description of the UKB GWAS QC procedures has been provided elsewhere (22) and is summarized here. We restricted our analysis to 438 870 participants who were either white-British or were genetically similar to white-British UKBB participants based on PCA (e.g. Irish ancestry) (21). In total, we included 2274 SCC cases, 16 847 BCC cases and 340 302 cancer free controls in the study. Using a common control set we performed a BCC, SCC and by combining the BCC and SCC case set as a KC phenotype GWAS. Note that there were 583 people who had both BCC and SCC and we included them in the individual BCC and SCC analyses as a case (Table 1).

The relatedness among individuals in UKBB is higher than that in a random population sample (30.3% of UKBB participants had third degree or closer relationships) (22). Hence, we used BOLT-LMM v1.2 (31) for our association analysis. BOLT-LMM uses linear mixed models to have better control over population stratification and cryptic relatedness than standard methods (31). We first used a sparse set of 368 802 genotyped autosomal SNPs to calibrate the Bayesian prior used for the actual BOLT-LMM association tests. We obtained association estimates for 97 million imputed SNPs. SNPs with poor imputation scores (INFO <0.4) or MAF <0.01 were discarded, yielding results for 7.6 million SNPs. Given that BOLT-LMM assumes that our trait of interest is quantitative, we applied a formula (32) to transform the linear-scale beta(s) into values on the log (OR) scale—using BOLT-LMM in this way is valid for analysis of large, non-ascertained population cohorts such as UKBB (31). The LDSC intercepts for the UKBB BCC and SCC GWAS were 1.03 and 1.00, respectively (Supplementary Material, Table S1).

#### eMERGE

We performed genotype QC using PLINK v1.90b5.4 (26,33) by removing SNPs with call rate <97%, MAF <0.01 and HWE P-value <10^−4^ in controls and *P*-value <10^−10^ in cases. Individuals with >3% missing genotypes were removed. Autosomal markers were used to compute IBD in PLINK, and one of each pair of related individuals with PI_HAT scores >0.2 were removed from the analysis. We conducted the PCA in PLINK using all the participants and reference samples of Northern European ancestry (1000G British, CEU, and Finland). We removed the ancestry outliers with PC1 and PC2 values more than six SD from the mean of the reference panel. Following QC there were 1565 cases with a KC diagnosis, 8756 cancer free controls, and 310 260 SNPs available for imputation (Table 1).

Phasing of the genotyped SNPs was performed in ShapeIT (34), and imputation in Minimac3 through the Michigan imputation server (29). We used HRC version r1.1 (28) as the reference panel for imputation. SNPs with MAF >0.001 and imputation quality (r^2^) >0.3 were used for association analysis. We performed the association testing using PLINK v2.00a2LM (26) under an additive genetic model adjusted for sex and the first 10 PCs. The LDSC intercept for the eMERGE GWAS was 1.00 [standard error (SE) = 0.007], indicating that there was very little evidence for stratification or structural biases (Supplementary Material, Table S1).

#### 23andMe

For detailed QC information see (12,35); this is summarized briefly here. Individuals with <97% European ancestry were identified and excluded using an ancestry deconvolution model (36). SNPs with genotype call rate <95%, HWE *P*-value <10^−20^ were removed from the analysis. If the allele frequencies were different in 23andMe compared to the European 1000 Genomes reference panel, they were also excluded from the analysis. Phasing of genotyped SNPs was performed using Beagle (version 3.3.1) (37). Imputation to version 3 release of the 1000 Genomes reference haplotypes (38) was conducted using Minimac2 (35). GWAS data using an overlapping set of controls were available for European ancestry participants of 23andMe for SCC (6579 cases and 280 558 controls) and BCC (12 945 cases and 274 252 controls) (Table 1). In our analysis we included 9 061 544 SNPs after excluding those variants with a MAF <0.01 and an imputation score <0.3. LDSC indicated little to no evidence of residual inflation (intercept SCC 1.04, BCC 1.06, Supplementary Material, Table S1).

### Genetic overlap

In light of the previously identified common risk factors and shared genes of SCC and BCC risk we hypothesized that BCC and SCC may have a high genetic correlation. Hence, we explored the genetic overlap between these skin cancers using the bivariate LDSC v.1.0.0 method (16). LDSC is a feasible way of estimating genetic correlations between traits using GWAS summary statistics (Supplementary Material, Table S1). Furthermore, the LDSC method appropriately accounts for overlapping samples when analysing GWAS summary statistics.

### Multiple trait analysis of KC

To model the high but incomplete correlation between BCC and SCC, we conducted a meta-analysis using multi-trait analysis of GWAS (MTAG), a method-of-moments framework, which requires only GWAS summary statistics for the included traits (39,40). MTAG models the incomplete correlation between the input traits using bivariate LDSC. MTAG has the useful property that due to the modelling done in LDSC, it accounts for sample overlap across the input traits (39,40). This feature is important as some of the datasets used in our analysis have a large degree overlap among the control sets (Table 1).

We performed a multiple trait combined KC GWAS analysis using; UKBB KC (18 538 cases, 340 302 controls), eMERGE KC (1565 cases, 8756 controls), 23andMe BCC (12 945 cases, 274 252 controls), 23andMe SCC (6579 cases, 280 558 controls) and QSkin KC (8145 cases, 4797 controls) (Methods; Table 1). MTAG has the advantage of outputting a result for each input trait adjusted by all other input traits; the results we present here are the MTAG output for the UKBB KC GWAS. (Supplementary Material, Table S2). Manhattan plot, Q-Q plot and the regional plots for the output of the UKBB KC GWAS MTAG output can be found in Supplementary Materials, Figures S2, S3 and S8.

### Conditional analysis and locus assignment

To identify all independent SNPs associated with KC in the MTAG analysis, we performed a joint conditional analysis using the GCTA v1.26 software (41), with stepwise model selection and the parameters set to distance of SNPs 2000Kb, threshold *P*-value 5 × 10^−8^ and the collinearity between the SNPs to 0.05 (Supplementary Material, Table S2). A LD reference panel was constructed using 5000 randomly selected participants from the UKBB. Independent SNPs within 1 mb (mega base) of each other were assigned to the same locus with the exception of SNPs around *ASIP* at 20q11 where long-range LD has been observed and a wider boundary of 2.5 mb was used.

### BCC and SCC meta-analyses

To identify genetic variants specific to BCC and SCC, we performed meta-analyses of the individual cancers using METAL (39). For BCC meta-analyses we used samples from UKBB BCC (16 847 cases, 340 302 controls), 23andMe BCC (12 945 cases, 274 252 controls) and QSkin BCC (1995 cases, 4797 controls). SCC GWAS was conducted using UKBB SCC (2274 cases, 340 302 controls), 23andMe SCC (6579 cases, 280 558 controls) and QSkin SCC (821 cases, 4797 controls). The full results for these analyses can be found in Supplementary Material, Table S3. Conditional analysis was performed for the BCC and SCC meta-analyses as above, and BCC and SCC loci were deemed the same as those identified in the KC analysis if they were within 1 mb of each other; the LD r^2^ between lead SNPs and the matching KC locus can be found in Supplementary Material, Table S3. Manhattan plots, Q-Q plots and the regional plots for the output of the BCC and SCC meta-analyses can be found in Supplementary Materials, Figures S4–S7, S9, S10.

### Estimating heritability

We calculated the heritability due to common SNPs (h^2^_SNP_) estimates on the liability scale using LDSC and assuming that the prevalence of BCC to be 3.5% and SCC to be 0.5%, respectively, in the UK (prevalence calculated using the proportions within UKBB). Briefly, GWAS results for UKBB BCC and UKBB SCC analyses were filtered to SNPs MAF >0.01%, and present in the Hapmap3 reference list provided by LDSC.

Then we computed the h^2^_SNP_ following the removal of the GWAS significant loci for BCC and SCC, respectively. For each loci we filtered out the top independent SNP and all SNPs within 1 mb either side. For *HLA* and *MC1R* this window was expanded to 2 mb, and for *ASIP* we removed 31 to 36 mb of chr20 due to the long range LD exhibited at this locus.

### Comparison of effect estimates between BCC, SCC and KC

To further explore the concordance in effect sizes for genetic variants identified in the multiple trait combined KC GWAS, or the BCC and SCC specific meta-analyses, we extracted the effect sizes and SE for each independent SNP from the three datasets. We used the difference between SCC and BCC log (OR) and its SE (computed under the approximation of independence across the samples) to test for a difference in log (OR). In total 95 independent SNPs were extracted, and *P*-value <~ 5 × 10^−4^ was assigned as the significance threshold (Supplementary Material, Table S8).

### Gene-based methods

We performed gene-based tests using the fastBAT (42) software, based on a Bonferroni-corrected significance threshold of 0.05/23957 = 2.1 × 10^−6^. Gene-based results with peak single SNP results >5 × 10^−8^ (that is, gene-based results not derived from genes containing a genome-wide significant SNP in their own right) are shown in Supplementary Material, Table S4. To ensure we were not capturing long-range LD with loci discovered in the single SNP GWAS, genes with significant gene-based results were deemed as novel loci if >1 mb from an SNP with a single SNP result *P*-value <5 × 10^−8^, and that the peak SNP in each gene region was not in LD (r^2^ >0.01) with any single SNP with a GWAS *P*-value <5 × 10^−8^ within 2 mb. Adjacent gene-based results were combined into a single locus if within 1 mb of each other (Supplementary Material, Table S4).

### Post-GWAS analysis

The overall GWAS results for BCC, SCC and KC were further examined using FUMA (43) to test whether any predefined gene sets show enrichment, based on the GWAS results (MAGMA gene set analysis). GWAS loci were also characterized in terms of eQTL data and potential drug targets using the Open Targets platform (44) and the Gtex portal (v7) (45) using the strongest single SNP within in each gene region (Tables 3 and 4; full results are in Supplementary Material, Table S2, Supplementary Material, Table S3, and Supplementary Material, Table S4).

### Analysis of predicted gene expression

To further understand the biological mechanisms of novel genome-wide SNPs, we performed a TWAS using the MetaXcan software, a method for relating predicted tissue expression levels of genes to phenotypes (46). We specifically investigated gene expression in whole blood (6759 genes), sun-exposed skin (7637 genes) and skin not exposed to the sun (5802 genes) from GTEx (45) (Supplementary Materials, Tables S5, S6 and S7). Associations were deemed significant at the Bonferroni-corrected threshold of 0.05/(20 198) = 2.48 × 10^−6^.

## Supplementary Material

suppl_data_ddz121
