# Supplementary material for: Combined analysis of keratinocyte cancers identifies novel genome-wide loci
Source: Hum Mol Genet. 2019 Jun 7;28(18):3148–60. doi: 10.1093/hmg/ddz121 (PMC6737293; doi:10.1093/hmg/ddz121)
Supplement: suppl_data_ddz121 [file suppl_data_ddz121.zip › KC_GWAS_supp_R1_(1)_ddz121.docx]

# Supplementary Data

# Combined analysis of keratinocyte cancers identifies novel genome-wide loci

## Authors

Upekha E. Liyanage^1^**^*^**, Matthew H. Law^1^, Xikun Han^1^, Jiyuan An^1^, Jue-Sheng Ong^1^, Puya Gharahkhani^1^, Scott Gordon^2^, Rachel E. Neale^3^, Catherine M. Olsen^4,5^, 23andMe Research Team, Stuart MacGregor^1a^, David C. Whiteman^4a^

## Affiliations

**^a^** Equal contribution

^1^ Statistical Genetics Lab, QIMR Berghofer Medical Research Institute, 300, Herston road, Brisbane, QLD 4006

^2^ Genetic Epidemiology, QIMR Berghofer Medical Research Institute, 300, Herston road, Brisbane, QLD 4006

^3^ Cancer Aetiology & Prevention, QIMR Berghofer Medical Research Institute, 300, Herston road, Brisbane, QLD 4006

^4^ Cancer Control Group, QIMR Berghofer Medical Research Institute, 300, Herston road, Brisbane, QLD 4006

^5^ Faculty of Medicine, University of Queensland, Brisbane, QLD

***Corresponding author:** Upekha Liyanage, Address - Statistical Genetics Lab, QIMR Berghofer Medical Research Institute, 300, Herston road, Brisbane, QLD 4006, Telephone - 0738453713, [upekha.liyanage@qimrberghofer.edu.au](mailto:upekha.liyanage@qimrberghofer.edu.au)

# Supplementary Tables

## Footnotes for tables in the supplemental worksheets.

(Supplementary Table 1 - 8 are provided as separate excel files.)

### [Supplementary Table 1](https://docs.google.com/spreadsheets/d/1ItQWzzdZNWNXapkMav7BCi4Ni_hutN8A-2tyFti8NGY/edit#gid=0): LD score analysis of individual GWAS datasets

The pairwise genetic correlations as measured by bivariate LD Score regression (LDSC) [(3)](https://paperpile.com/c/eIABLH/3nwl) of indicated GWAS (**Phenotype 1, P1, and Phenotype 2, P2**) are shown in the rg column; Standard error(**SE**) and P-value (**P**) of rg is reported. For each comparison the GWAS lambda (λ) and LD Score intercept is displayed.

### [Supplementary Table 2](https://docs.google.com/spreadsheets/d/1ItQWzzdZNWNXapkMav7BCi4Ni_hutN8A-2tyFti8NGY/edit#gid=1511085375): Complete results for independent SNPs reach genome-wide significance for MTAG analysis of KC

Chromosome (**CHR**), region and positions (**BP**) displayed using hg19 coordinates. Loci reaching genome-wide significance are counted in order (**Locus**), with the peak and any independent secondary SNPs for each locus reported in **SNP**. The effect and non effect allele (**EA/NEA**), effect allele frequency (**EAF**), P-value (**P**), log(Odds Ratio) (**BETA**), standard error (**SE**) as well as the odds ratio and its 95% confidence interval (**OR [95% CI]**) from the MTAG analysis [(4)](https://paperpile.com/c/eIABLH/0sLR) output for the UKBB KC datasets are also reported.

GCTA joint conditional analysis (GCTA-cojo) [(5)](https://paperpile.com/c/eIABLH/zerR) was used to identify independent SNPs, and the conditional P-value from fitting all independent SNPs jointly is provided (**Joint conditional P**).

Genomic context, including the **Nearest gene** and **Nearest protein coding gene** are reported, as is whether the locus is novel (**Novel?** column) in terms of prior publication for BCC, SCC or KC; if not the associated trait is indicated, and the relevant publication provided. TWAS SCC indicates the gene was associated with SCC using a transcriptome wide association study (TWAS)[(6)](https://paperpile.com/c/eIABLH/GdhY) approach; see [(7)](https://paperpile.com/c/eIABLH/cogE).

Annotation information, including GTex v7 [(8)](https://paperpile.com/c/eIABLH/GeVU) **eQTL results** in the format of gene names with tissue and P-value in brackets are listed if significance (P < 2.1 × 10^-6^). These same eQTL results are further summarised by tissues likely to be involved in KC (skin and whole blood to encapsulate immune cells) or all other GTEx tissue the the columns labelled **eQTL Skin**, **eQTL Whole Blood**, and **eQTL Other Tissue**. We also report via the Opentargets platform [(9)](https://paperpile.com/c/eIABLH/DHR7) a summary of GWAS summary **(GWAS summary)**, PheWAS (**PheWAS summary**) results for the peak SNP, and if any of the implicated genes listed for this SNP are drug targets (**Drug(s)**) and indicate if these drugs are utilised for treatment or control of skin cancer.

###

### [Supplementary Table 3](https://docs.google.com/spreadsheets/d/1ItQWzzdZNWNXapkMav7BCi4Ni_hutN8A-2tyFti8NGY/edit#gid=1953256512): Complete results for independent SNPs reach genome-wide significance for fixed effect meta-analysis of BCC or SCC

Using the same column format as Supplementary Table 2 the independent, genome-wide significant SNPs for the METAL meta-analysis [(10)](https://paperpile.com/c/eIABLH/ZaT3) of BCC or SCC GWAS datasets are provided. Additional fields are the locus number assigned in Supplementary Table 2 if overlapping (**Overlapping KC locus**), LD r^2^ between lead SNPs and the matching KC locus (**r^2^**), or if novel to the KC MTAG analysis [(4)](https://paperpile.com/c/eIABLH/0sLR) (**Novel locus to BCC or SCC META relative to KC MTAG ?**). Where loci overlap with those discovered in the KC MTAG analysis, we provide the relevant GWAS summary statistics (**MTAG KC BETA** etc) in addition to the fixed effect meta-analysis results (**METAL BETA** etc)

[**Supplementary Table 4**](https://docs.google.com/spreadsheets/d/1ItQWzzdZNWNXapkMav7BCi4Ni_hutN8A-2tyFti8NGY/edit#gid=1623983545)**: FastBAT gene-based analysis of KC GWAS meta-analysis**

FastBAT[(11)](https://paperpile.com/c/eIABLH/a2rM) Gene based results were filtered for gene-based significance (Methods) and further filtered if the gene region contained a SNP reaching genome-wide significance in the MTAG[(4)](https://paperpile.com/c/eIABLH/0sLR) GWAS results. The remaining gene based results were assigned to an existing locus if within 1 Mb (megabase), and/or the lead SNP for the locus and the gene were in LD r^2^ > 0.01.

**Novel Locus** indicates if the locus has been reported previously or identified by the single SNP GWAS performed in this analysis. New loci are numbered sequentially, and newly identified genes within 1 Mb of one another are assigned to the same locus; LD between the peak SNP in adjacent genes is listed (**r^2^ with nearby gene peaks**). **Gene** name is reported, as is the hg19 positional information including gene start and stop.

The nearest **KC MTAG**, or **BCC**/**SCC** GWAS meta-analysis loci are reported in the relevant columns. The LD r^2^ between the GWAS loci peak SNP and the strongest SNP in the gene-based region is listed in the “**LD** **r2 with overlapping SNP**” column, as is if the gene is within 1Mb of a known locus.

The top SNP within each gene, its P-value and the FastBAT gene-based P-value is shown in the **TopSNP**, **TopSNP P,** and **Gene-based** **P** columns.

The **eQTL** and **PheWAS** annotation columns are as described in Supplementary Tables 2 and 3.

##

## [Supplementary Table 5](https://docs.google.com/spreadsheets/d/1ItQWzzdZNWNXapkMav7BCi4Ni_hutN8A-2tyFti8NGY/edit#gid=775565848): MetaXcan gene-based test using sun exposed skin expression data

TWAS MetaXcan [(6)](https://paperpile.com/c/eIABLH/GdhY) results were filtered to the Bonferroni corrected threshold of 2.48 × 10^-6 .^ Where the genes overlap with a locus identified in the single SNP based analysis, the details for the overlapping KC MTAG [(4)](https://paperpile.com/c/eIABLH/0sLR) locus and, and its peak SNP is provided (**Region**, **BP**, **Overlapping KC Locus**, and **Overlapping KC SNP**).

Hg19 chromosome (**CHR**), positional information (**Gene position**), ensemble ID (**Gene**), and (**Gene name)** are tabulated, as are the summary TWAS results from metaXcan. **Effect size**, P- value (**P**), and **Z-score** for the association between phenotype (sun exposed skin) and the predicted gene expression are reported. The variance in gene expression explained by the chosen SNPs (**Var g)**, Cross-validated R2 value (**Pred perf R2**), P-value of the correlation between cross-validated prediction and observed expression (**Pred perf p**), q-value obtained when analyzing the initial distribution of P-values (**Pred perf q**), number of SNPs used to predict the expression level of the gene (**Number of SNPs used**), number of SNPs in covariance (**Number of SNPs in cov**), number of SNPs used to predict the expression level of the gene, number of SNPs in the model (**Number of SNPs in model**) are reported.

### [Supplementary Table 6](https://docs.google.com/spreadsheets/d/1ItQWzzdZNWNXapkMav7BCi4Ni_hutN8A-2tyFti8NGY/edit#gid=1347900941): MetaXcan gene-based test using non sun exposed skin expression data

### Putative target genes of our MTAG [(4)](https://paperpile.com/c/eIABLH/0sLR) KC results related to non sun exposed skin are reported here with the same column format of the Supplementary Table 5.

##

## [Supplementary Table 7](https://docs.google.com/spreadsheets/d/1ItQWzzdZNWNXapkMav7BCi4Ni_hutN8A-2tyFti8NGY/edit#gid=1115625039): MetaXcan gene-based test using whole blood expression data

### Putative target genes of our MTAG [(4)](https://paperpile.com/c/eIABLH/0sLR) KC results related to blood are reported here with the same column format of the Supplementary Table 5.

[**Supplementary Table 8**](https://docs.google.com/spreadsheets/d/1unYOWRNyBNC97O-H2J6ChzuX3T-DJrJNNs-7oNLWwwc/edit#gid=282527902)**: Comparison of KC, BCC, SCC effect estimates**

Chromosome (**CHR**), positions (**BP**) effect and non effect allele (**EA/NEA**), P-value (**P**), log (Odds Ratio) (**BETA**), standard error (**SE**) as well as the **nearest gene** and **nearest protein coding gene** are reported for all the GWAS significant KC, BCC, SCC SNPs. For each SNP, Effect estimate (**BETA**), Standard Error (**SE**), odds ratio and its 95% confidence interval (**OR [95% CI]**), P-value (**P**) from the MTAG analysis [(4)](https://paperpile.com/c/eIABLH/0sLR) output for the UKBB KC datasets, BCC meta-analysis and SCC meta-analysis are also reported for the comparison. Ambiguous SNPs are replaced with the highest LD proxy SNPs: rs6736111 for *rs7563677 (C/G), r^2^=0.55; rs9396592 for *rs9383064 (C/G), r^2^=0.63; rs2721934 for *rs2721936 (A/T) r^2^=0.97; rs4977494 for *rs60269255(C/G), r^2^=0.99; rs12984428 for *rs7508601(A/T) r^2^=0.42. ***^a^***Of note we included rs9383064(C/G) SNP information which is not reported in MTAG KC UKBB output and we report the UKBB KC GWAS BETA effect estimates.

##

# Supplementary Figures

### Supplementary Figure 1: Layers of the skin representing squamous cells and basal cells

Source: Wikimedia commons

**
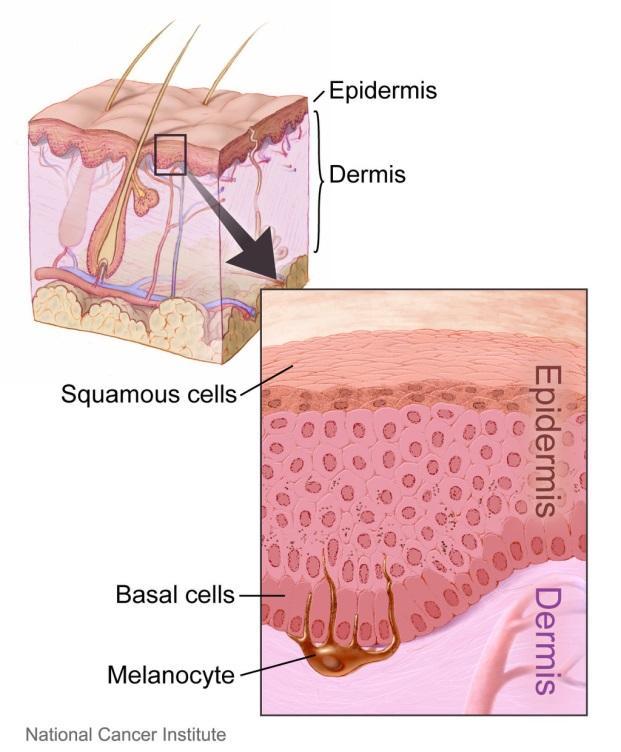
**

### Supplementary Figure 2: Manhattan plot of KC multi-trait analysis for UKBB KC output

Manhattan plot generated using FUMA [(1)](https://paperpile.com/c/eIABLH/gHMa). Corresponding negative log 10 P-value of each SNP (Y -axis) is plotted against the position of SNP in ascending order of each chromosome (X-axis).

**
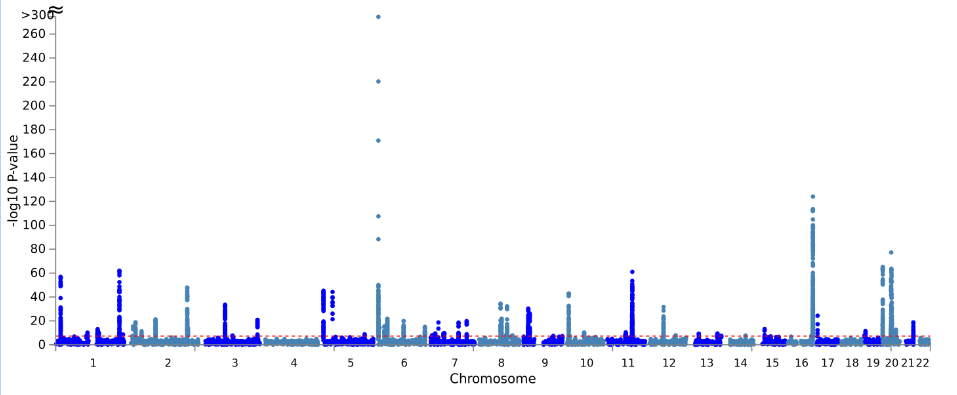
**

### Supplementary Figure 3: Q-Q plot KC multi-trait analysis for UKBB KC output

Quantile-Quantile plot generated using FUMA [(1)](https://paperpile.com/c/eIABLH/gHMa) showing observed and expected P-values.

**
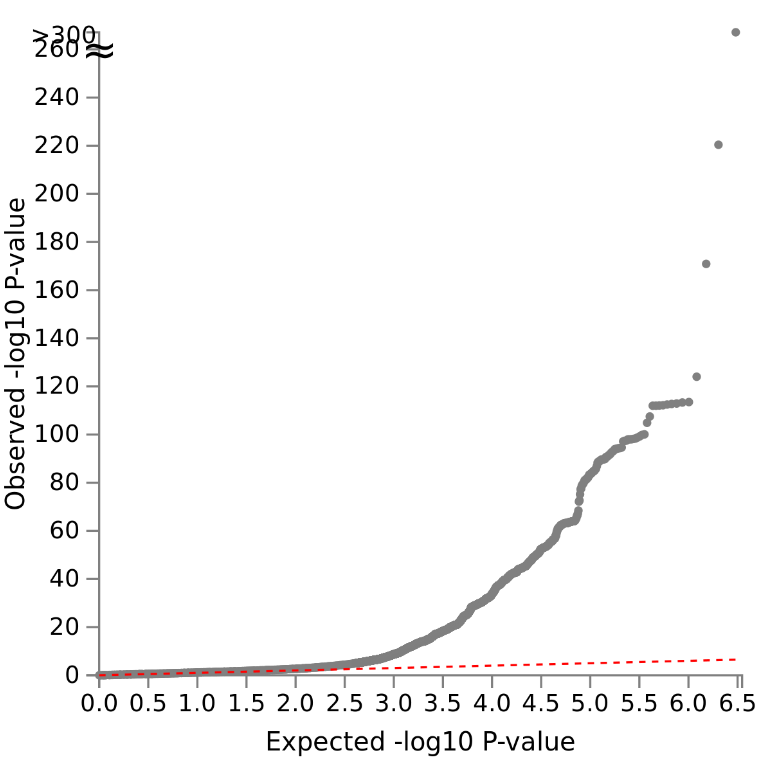
**

### Supplementary Figure 4: Manhattan plot BCC meta-analysis

Manhattan plot generated using FUMA [(1)](https://paperpile.com/c/eIABLH/gHMa). Corresponding negative log 10 P-value of each SNP (Y -axis) is plotted against the position of SNP in ascending order of each chromosome (X-axis).

**
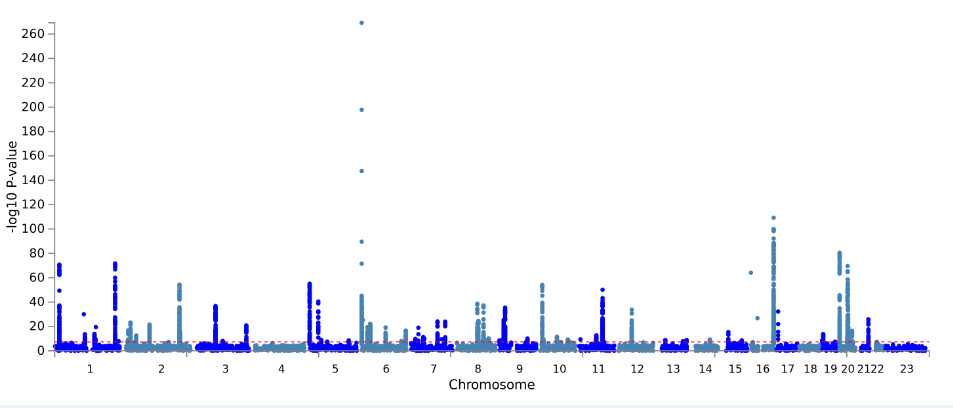
**

### Supplementary Figure 5: Q-Q plot BCC meta-analysis

Quantile-Quantile plot generated using FUMA [(1)](https://paperpile.com/c/eIABLH/gHMa) showing observed and expected P-values.

**
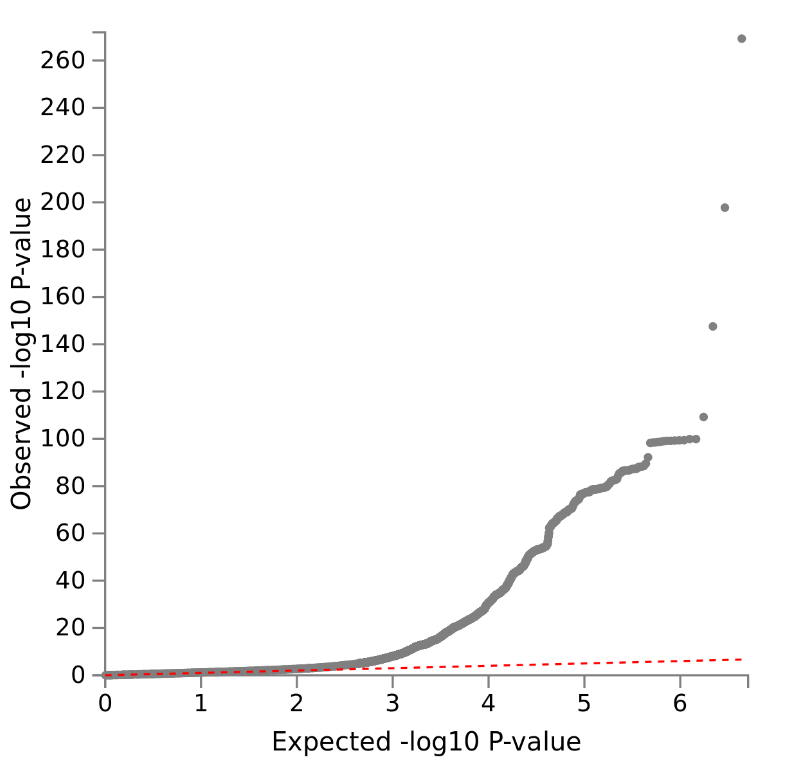
**

###

### Supplementary Figure 6: Manhattan plot SCC meta-analysis

Manhattan plot generated using FUMA [(1)](https://paperpile.com/c/eIABLH/gHMa). Corresponding negative log 10 P-value of each SNP (Y -axis) is plotted against the position of SNP in ascending order of each chromosome (X-axis).

**
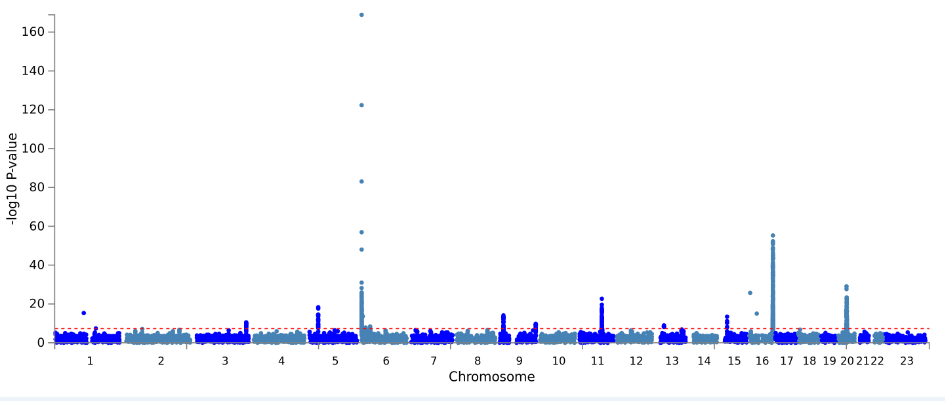
**

**Supplementary Figure 7: Q-Q plot SCC meta-analysis**

Quantile-Quantile plot generated using FUMA [(1)](https://paperpile.com/c/eIABLH/gHMa) showing observed and expected P-values.

**
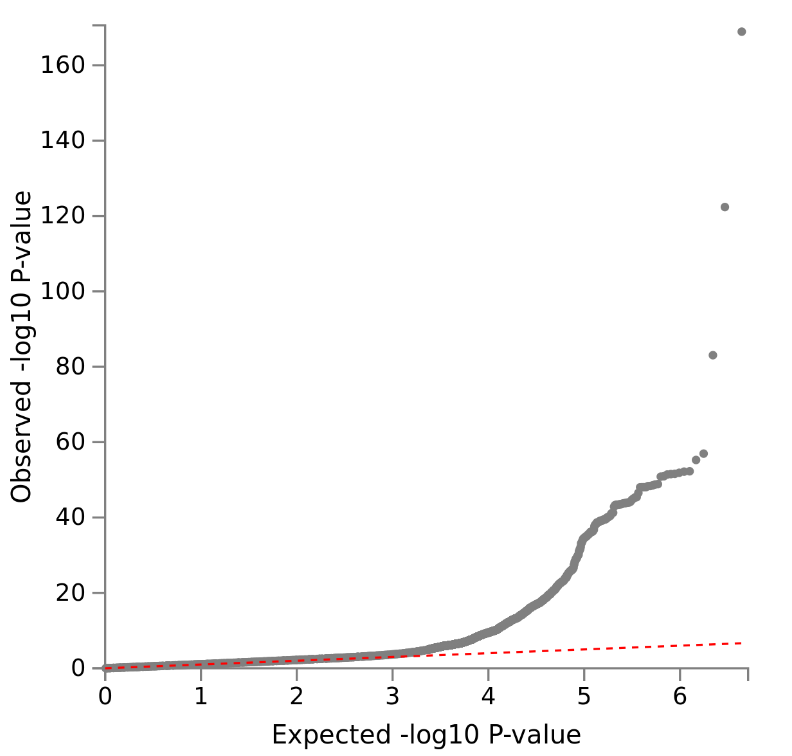
**

**Supplementary Figure 8: Regional association plots for independent SNPs reaching significance for multi-trait analysis of KC**

(Regional association plots are attached separately)

Significant SNPs of multi-trait analysis of KC of UKBB output have been displayed here with the genomic position (Mb) and the negative log 10 of P-value. Plots were generated using LocusZoom [(2)](https://paperpile.com/c/eIABLH/zCO2). rsID of peak SNP is mentioned and illustrated in purple. Nearby SNPs are assigned different colors in regards to the LD r2 with the peak SNP. For P-values and effect sizes of the peak SNP see Supplementary Table 2.

**Supplementary Figure 9: Regional association plots for independent novel SNPs reaching significance for meta-analysis of BCC**

(Regional association plots are attached separately)

Novel SNPs reached GWAS significance in BCC meta-analysis is displayed here with genomic position (Mb) and the negative log 10 of P-value. Plots were generated using LocusZoom [(2)](https://paperpile.com/c/eIABLH/zCO2). rsID of peak SNP is mentioned and illustrated in purple. Nearby SNPs are assigned different colors in regards to the LD r2 with the peak SNP. For P-values and effect sizes of the peak SNP see Supplementary Table 3.

**Supplementary Figure 10: Regional association plot for independent SNPs reaching significance for meta-analysis of SCC**

(Regional association plots are attached separately)

Novel SNPs reached GWAS significance in SCC meta-analysis is displayed here with genomic position (Mb) and the negative log 10 of P-value. Plots were generated using LocusZoom [(2)](https://paperpile.com/c/eIABLH/zCO2). rsID of peak SNP is mentioned and illustrated in purple. Nearby SNPs are assigned different colors in regards to the LD r2 with the peak SNP. For P-values and effect sizes of the peak SNP see Supplementary Table 3.

#

## Supplementary Methods

##

### Inclusion criteria for BCC and SCC cases in UK Biobank data

To be a SCC case, need to have

#ICD10 invasive

anything starting from C44 (e.g. C447, C442 etc) in f.40006.0.0 PLUS 8070-8076 or 8078 in f.40011.0.0

or

anything starting from C44 (e.g. C447, C442 etc) in f.40006.1.0 PLUS 8070-8076 or 8078 in f.40011.1.0 (the change is f.40006.0.0 -> f.40006.1.0 and f.40011.0.0 to f.40011.1.0)

or

...etc down to x=31 in f.40006.x.0 and f.40011.x.0

#ICD9 invasive

or

anything starting 173 (e.g. 1730, 1731 etc) in f.40013.0.0 PLUS 8070-8076 or 8078 in f.40011.0.0 (this is ICD9 codes; above for f.40006.0.0 was ICD10)

or

anything starting 173 (e.g. 1730, 1731 etc) in f.40013.1.0 PLUS 8070-8076 or 8078 in f.40011.0.0

or

...etc down to x=29 in f.40006.x.0 and f.40011.x.0

To be a BCC case, need to have,

#ICD10 invasive

anything starting C44 (e.g. C447, C442 etc) in f.40006.0.0 PLUS 8090-8094 or 8097-98 in f.40011.0.0

or

anything starting C44 (e.g. C447, C442 etc) in f.40006.1.0 PLUS 8090-8094 or 8097-98 in f.40011.1.0 (the change is f.40006.0.0 -> f.40006.1.0 and f.40011.0.0 to f.40011.1.0)

or

...etc down to x=31 in f.40006.x.0 and f.40011.x.0

#ICD9 invasive

or

anything starting 173 (e.g. 1730, 1731 etc) in f.40013.0.0 PLUS 8090-8094 or 8097-98 in f.40011.0.0 (this is ICD9 codes; above for f.40006.0.0 was ICD10)

or

anything starting 173 (e.g. 1730, 1731 etc) in f.40013.1.0 PLUS 8090-8094 or 8097-98 in f.40011.0.0

or

...etc down to x=29 in f.40006.x.0 and f.40011.x.0

**References**

[1. Watanabe,K., Taskesen,E., van Bochoven,A. and Posthuma,D. (2017) Functional mapping and annotation of genetic associations with FUMA. *Nat. Commun.*, **8**, 1826.](http://paperpile.com/b/eIABLH/gHMa)

[2. Pruim,R.J., Welch,R.P., Sanna,S., Teslovich,T.M., Chines,P.S., Gliedt,T.P., Boehnke,M., Abecasis,G.R. and Willer,C.J. (2010) LocusZoom: regional visualization of genome-wide association scan results. *Bioinformatics*, **26**, 2336–2337.](http://paperpile.com/b/eIABLH/zCO2)

[3. Bulik-Sullivan,B., Finucane,H.K., Anttila,V., Gusev,A., Day,F.R., Loh,P.-R., ReproGen Consortium, Psychiatric Genomics Consortium, Genetic Consortium for Anorexia Nervosa of the Wellcome Trust Case Control Consortium 3, Duncan,L., *et al.* (2015) An atlas of genetic correlations across human diseases and traits. *Nat. Genet.*, **47**, 1236–1241.](http://paperpile.com/b/eIABLH/3nwl)

[4. Turley,P., Walters,R.K., Maghzian,O., Okbay,A., Lee,J.J., Fontana,M.A., Nguyen-Viet,T.A., Wedow,R., Zacher,M., Furlotte,N.A., *et al.* (2018) Multi-trait analysis of genome-wide association summary statistics using MTAG. *Nat. Genet.*, **50**, 229–237.](http://paperpile.com/b/eIABLH/0sLR)

[5. Yang,J., Lee,S.H., Goddard,M.E. and Visscher,P.M. (2011) GCTA: A Tool for Genome-wide Complex Trait Analysis. *Am. J. Hum. Genet.*, **88**, 76–82.](http://paperpile.com/b/eIABLH/zerR)

[6. Barbeira,A.N., Dickinson,S.P., Torres,J.M., Bonazzola,R., Zheng,J., Torstenson,E.S., Wheeler,H.E., Shah,K.P., Edwards,T., Garcia,T., *et al.* Exploring the phenotypic consequences of tissue specific gene expression variation inferred from GWAS summary statistics.](http://paperpile.com/b/eIABLH/GdhY) [10.1101/045260](http://dx.doi.org/10.1101/045260)[.](http://paperpile.com/b/eIABLH/GdhY)

[7. Ioannidis,N.M., Wang,W., Furlotte,N.A., Hinds,D.A., 23andMe Research Team, Bustamante,C.D., Jorgenson,E., Asgari,M.M. and Whittemore,A.S. (2018) Gene expression imputation identifies candidate genes and susceptibility loci associated with cutaneous squamous cell carcinoma. *Nat. Commun.*, **9**, 4264.](http://paperpile.com/b/eIABLH/cogE)

[8. Carithers,L.J. and Moore,H.M. (2015) The Genotype-Tissue Expression (GTEx) Project. *Biopreservation and Biobanking*, **13**, 307–308.](http://paperpile.com/b/eIABLH/GeVU)

[9. Carvalho-Silva,D., Pierleoni,A., Pignatelli,M., Ong,C., Fumis,L., Karamanis,N., Carmona,M., Faulconbridge,A., Hercules,A., McAuley,E., *et al.* (2019) Open Targets Platform: new developments and updates two years on. *Nucleic Acids Res.*, **47**, D1056–D1065.](http://paperpile.com/b/eIABLH/DHR7)

[10. Willer,C.J., Li,Y. and Abecasis,G.R. (2010) METAL: fast and efficient meta-analysis of genomewide association scans. *Bioinformatics*, **26**, 2190–2191.](http://paperpile.com/b/eIABLH/ZaT3)

[11. Bakshi,A., Zhu,Z., Vinkhuyzen,A.A.E., Hill,W.D., McRae,A.F., Visscher,P.M. and Yang,J. (2016) Fast set-based association analysis using summary data from GWAS identifies novel gene loci for human complex traits. *Sci. Rep.*, **6**, 32894.](http://paperpile.com/b/eIABLH/a2rM)

##

## Abbreviations

ASR - Age-standardised incidence rate

BCC - Basal cell carcinoma

CI - Confidence interval

eMERGE - Electronic Medical Records for Genetic Research

eQTL - Expression quantitative trait loci

GWAS - Genome-wide association study

h^2^_SNP -_ SNP-heritability

HRC - Haplotype reference consortium

HWE - Hardy-Weinberg equilibrium

IBD - Identity by descent

KC - Keratinocyte carcinoma

LD - Linkage disequilibrium

LDSC - LD Score regression

MAF - Minor allele frequency

mb - Megabase

MTAG - Multi-Trait analysis of GWAS

OR - Odds ratio

PC - Principal components

PCA - Principal components analysis

QC - Quality control

SCC - Squamous cell carcinoma

SD - Standard deviation

SE - Standard error

SNPs - Single nucleotide polymorphisms

TWAS - Transcriptome wide association study

UKBB - UK Biobank

UVR - Ultraviolet radiation
