# Supplementary figures and images for: Combined analysis of keratinocyte cancers identifies novel genome-wide loci
Source: Hum Mol Genet. 2019 Jun 7;28(18):3148–60. doi: 10.1093/hmg/ddz121 (PMC6737293; doi:10.1093/hmg/ddz121)

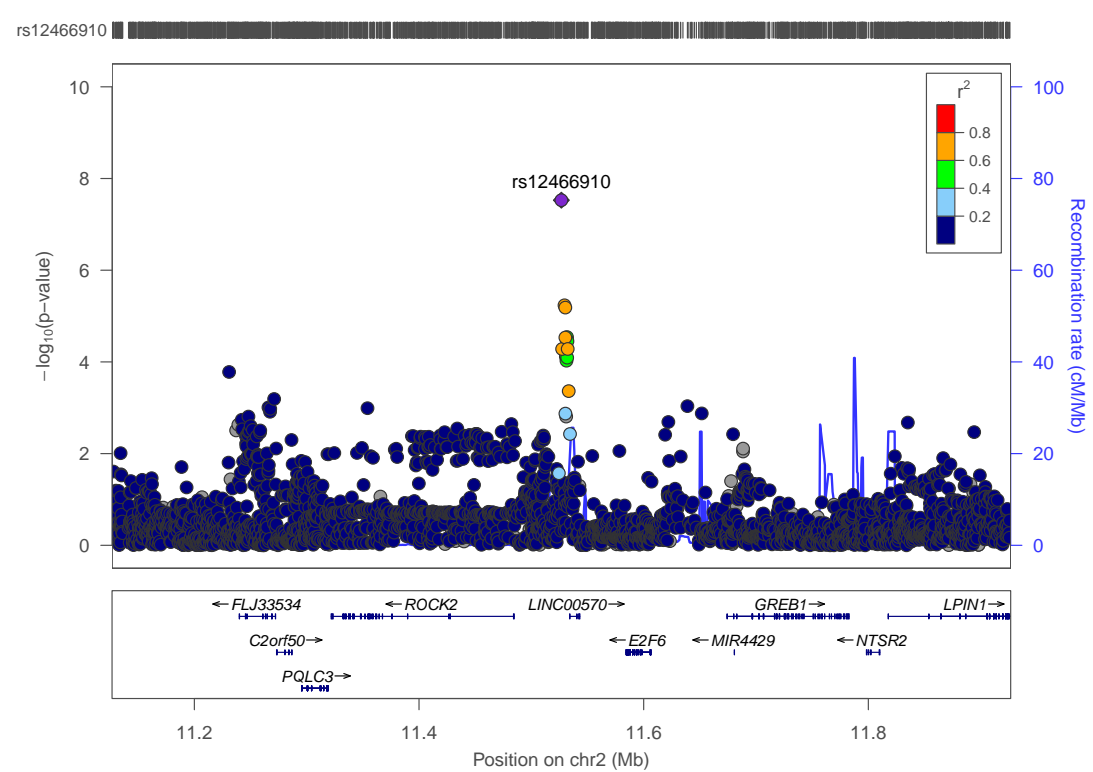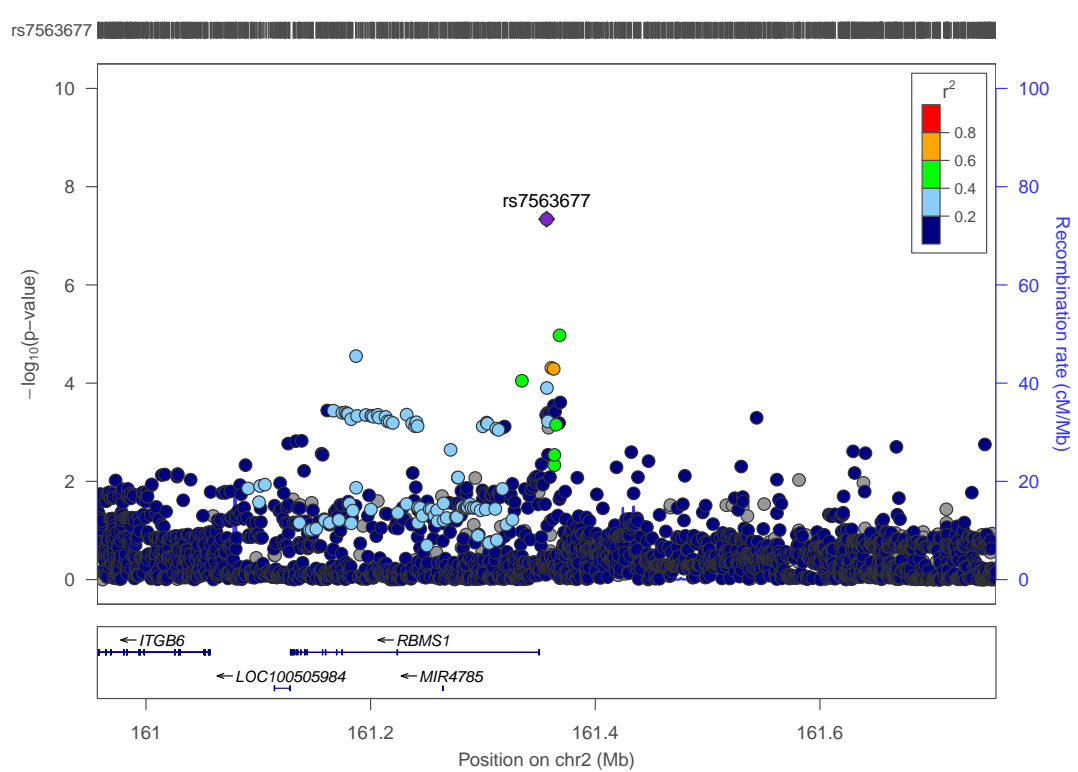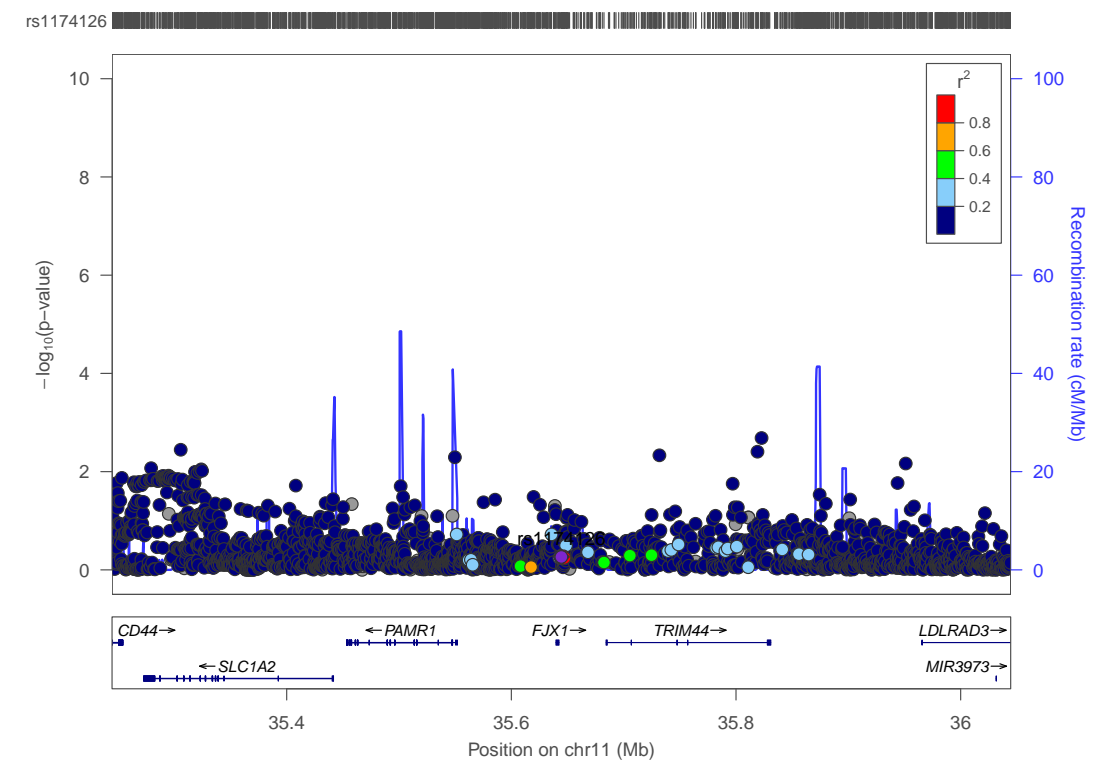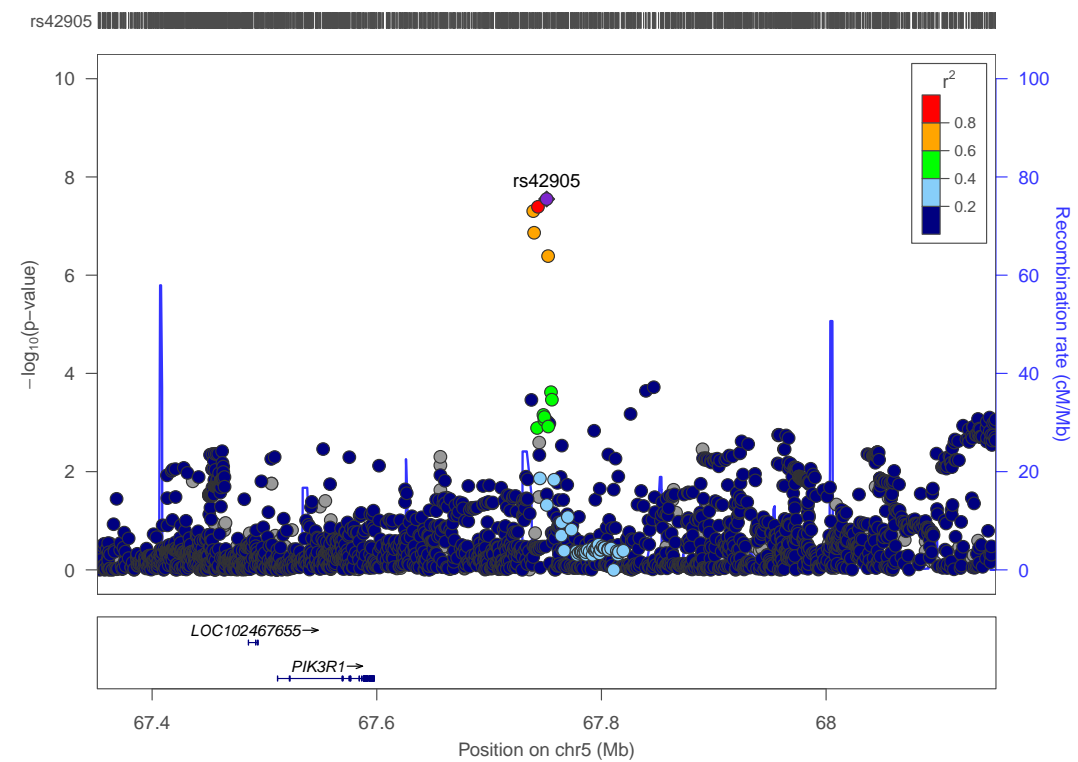

rs1220519

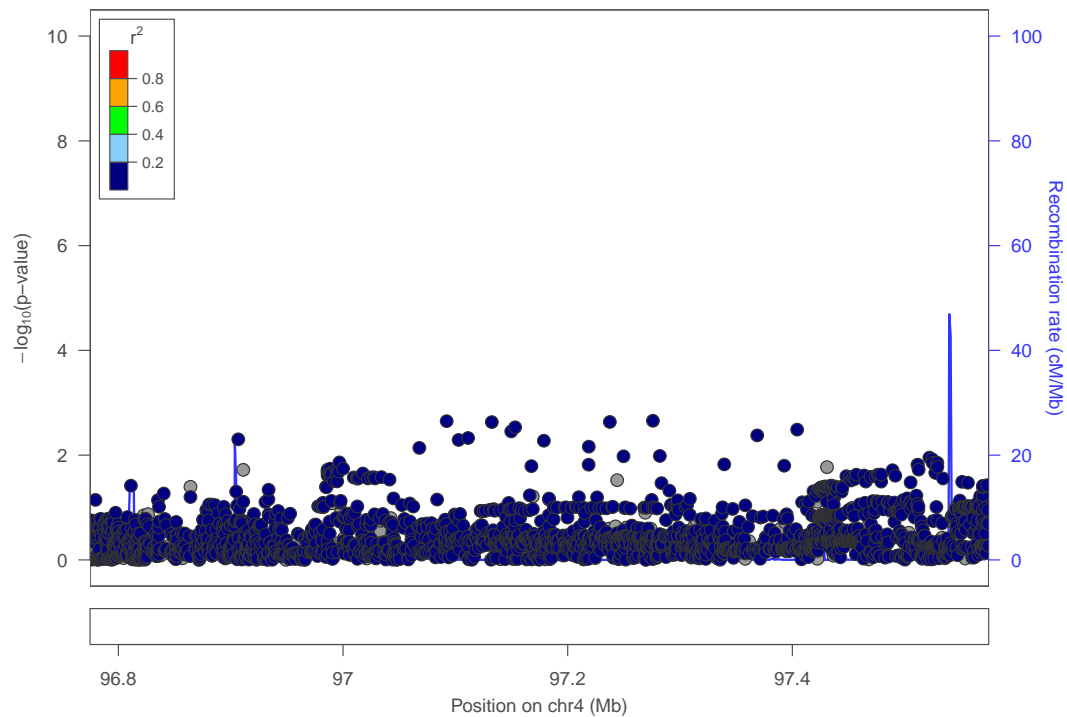

rs2721936

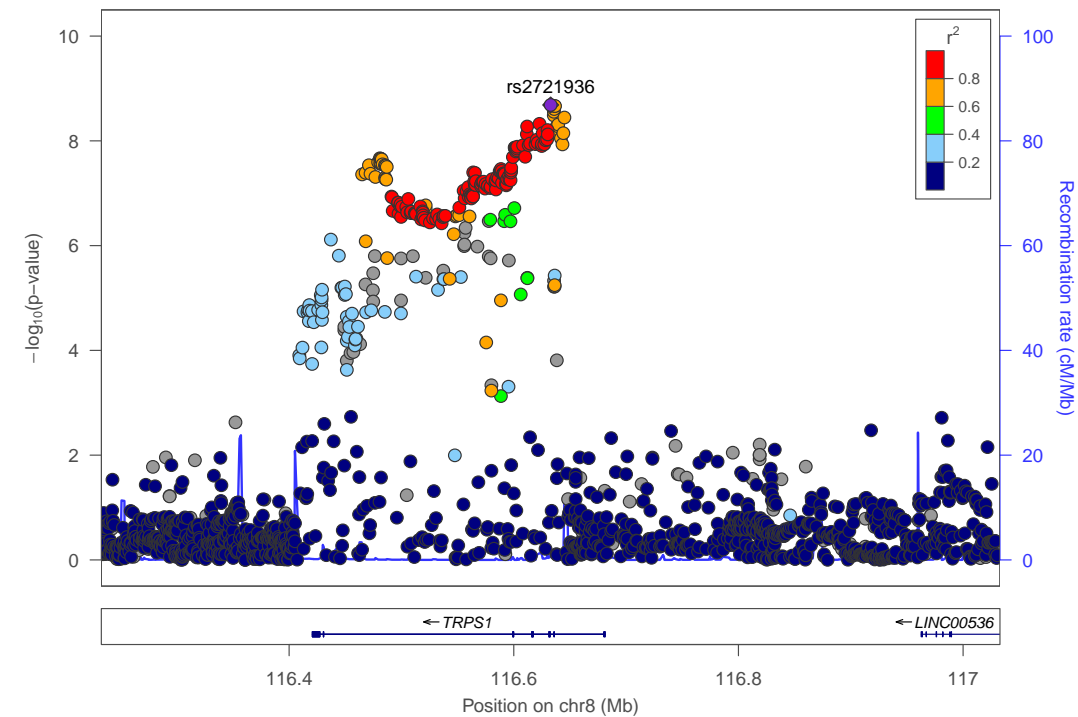

rs9408674

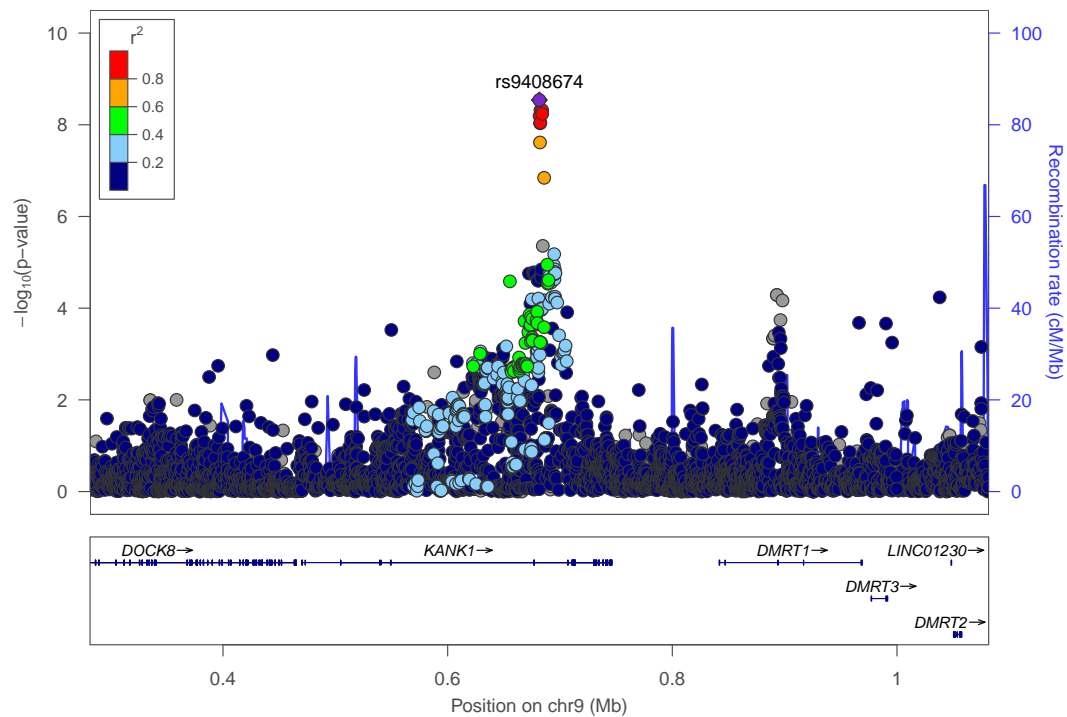

rs60269255

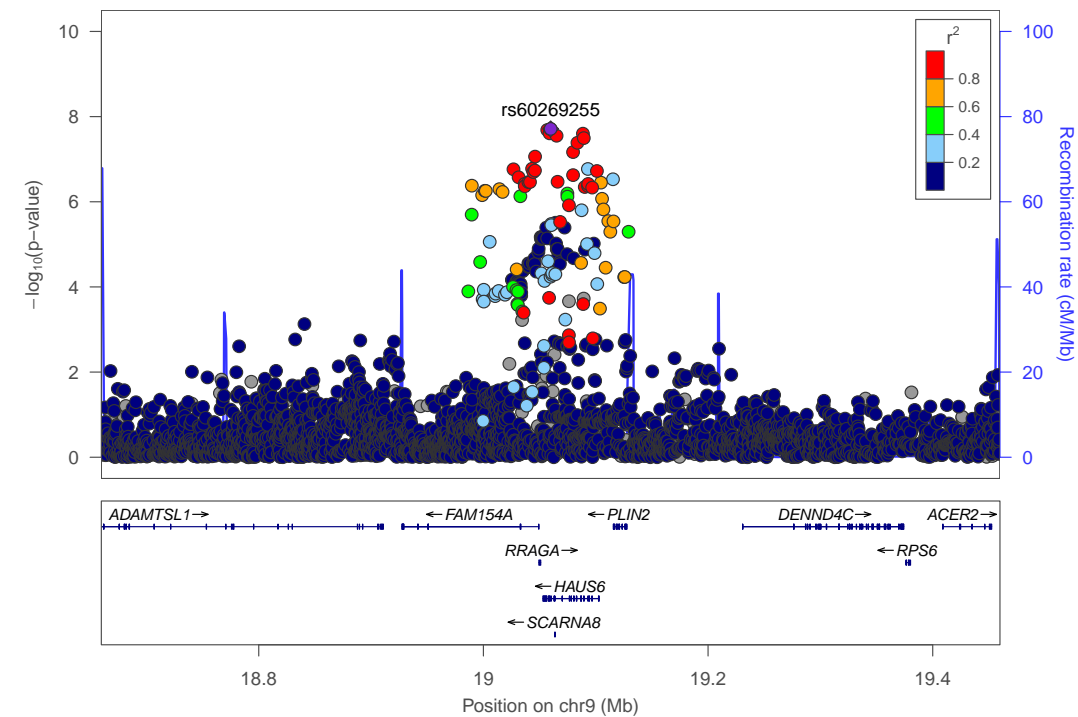

rs1887004

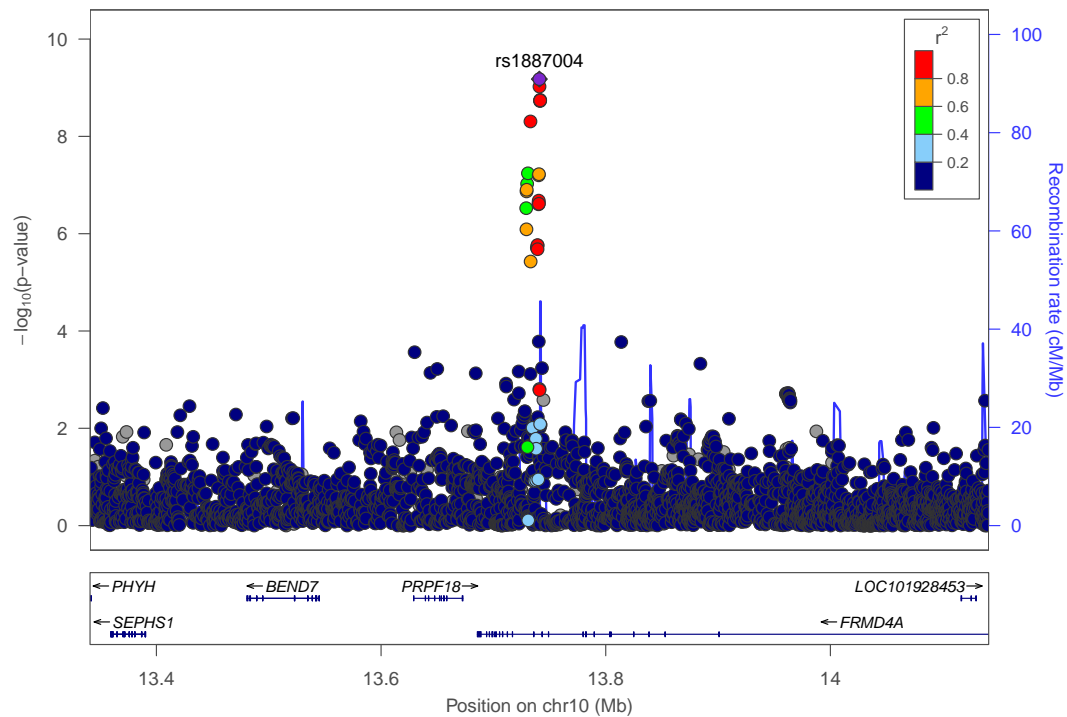

rs2289702

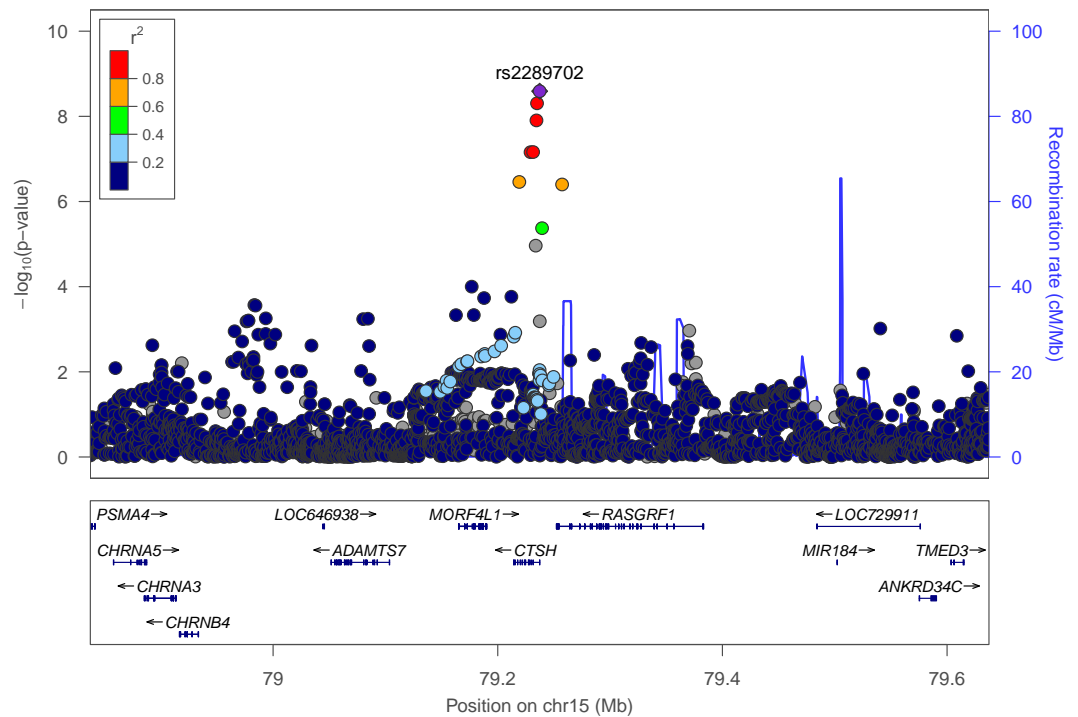

rs7508601

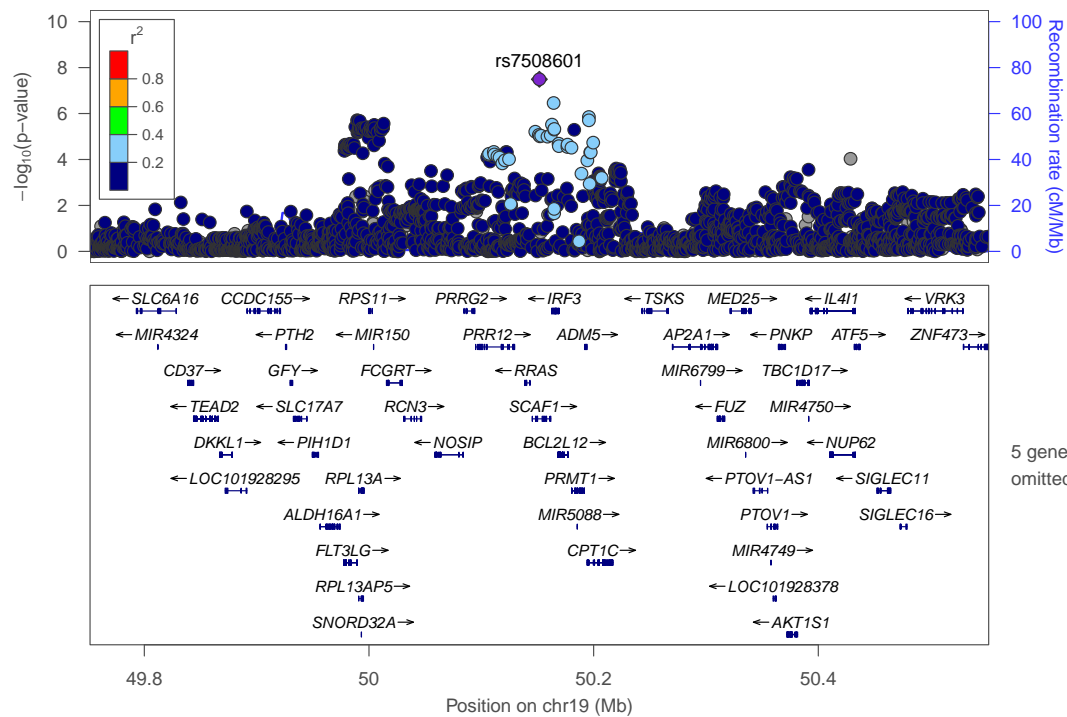

rs209901

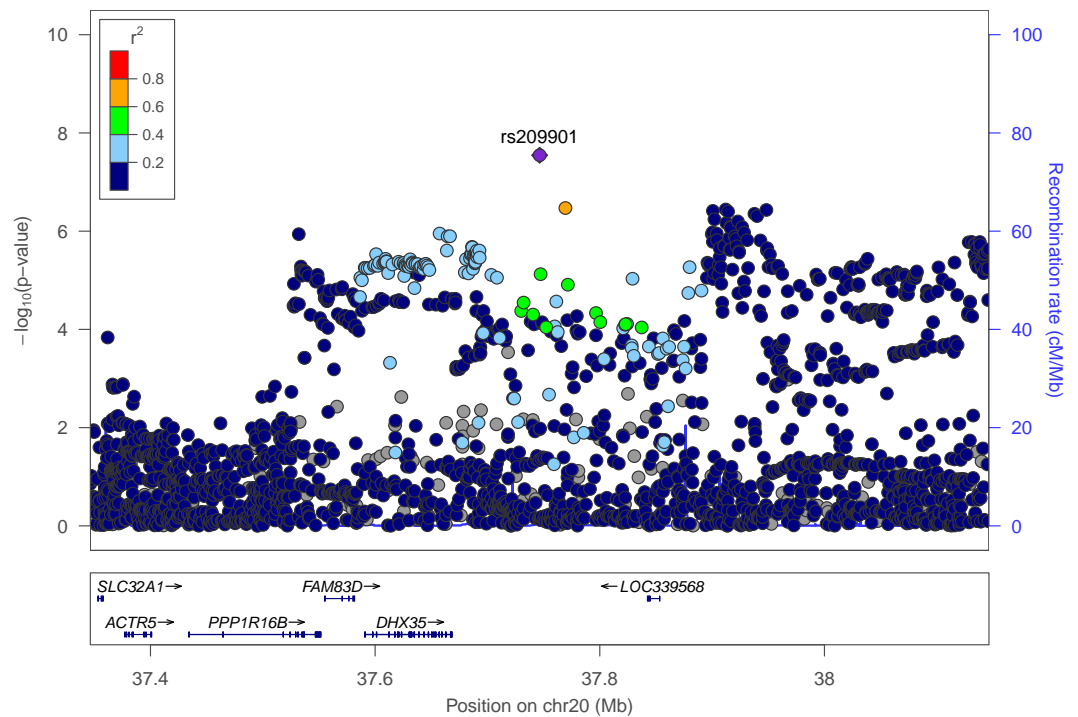

Supplement: suppl_data_ddz121 [file suppl_data_ddz121.zip › BCC_regional_plot_Supplementary_Figure_9_ddz121.pdf]

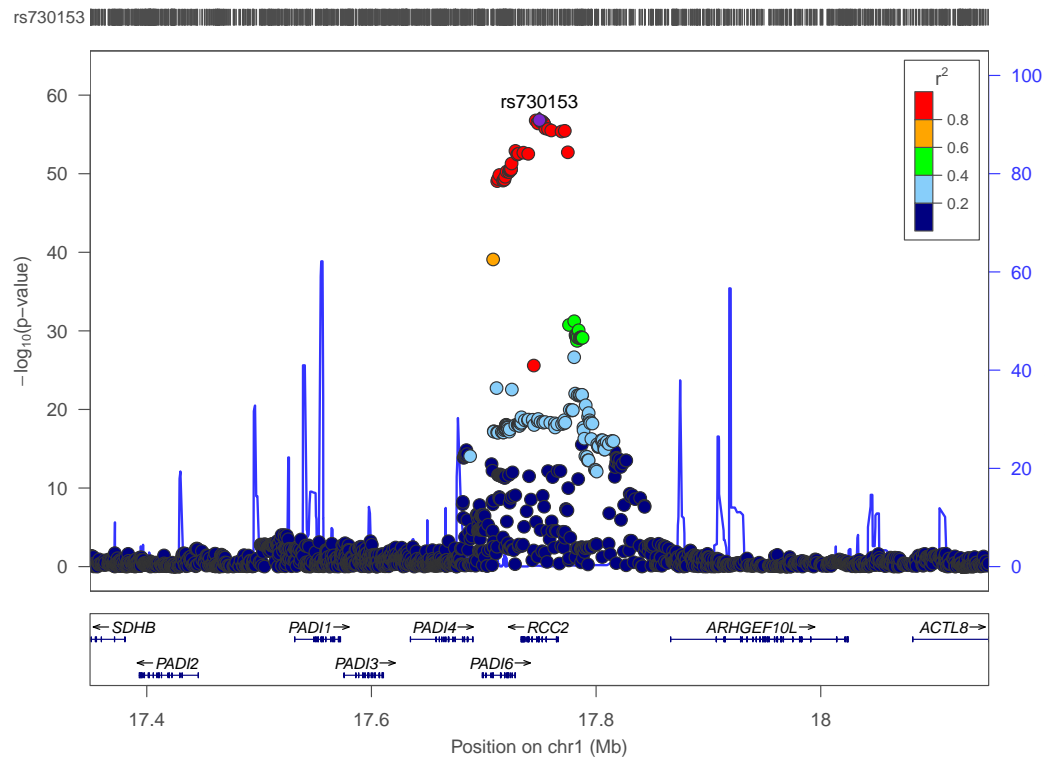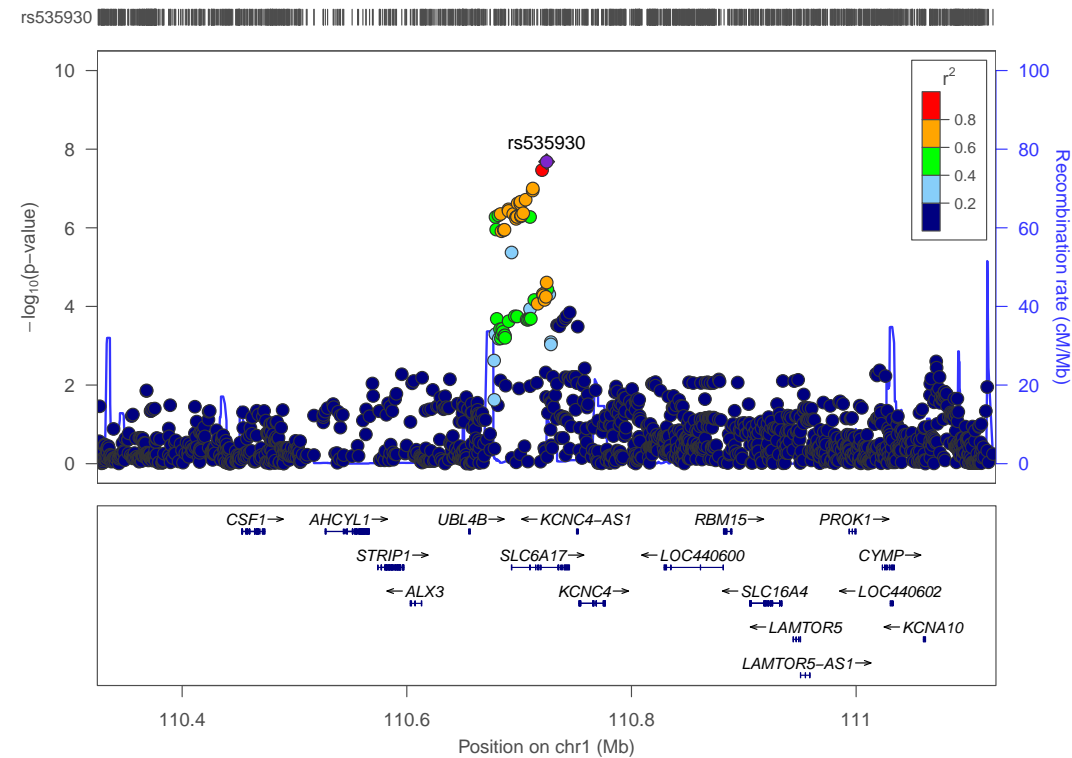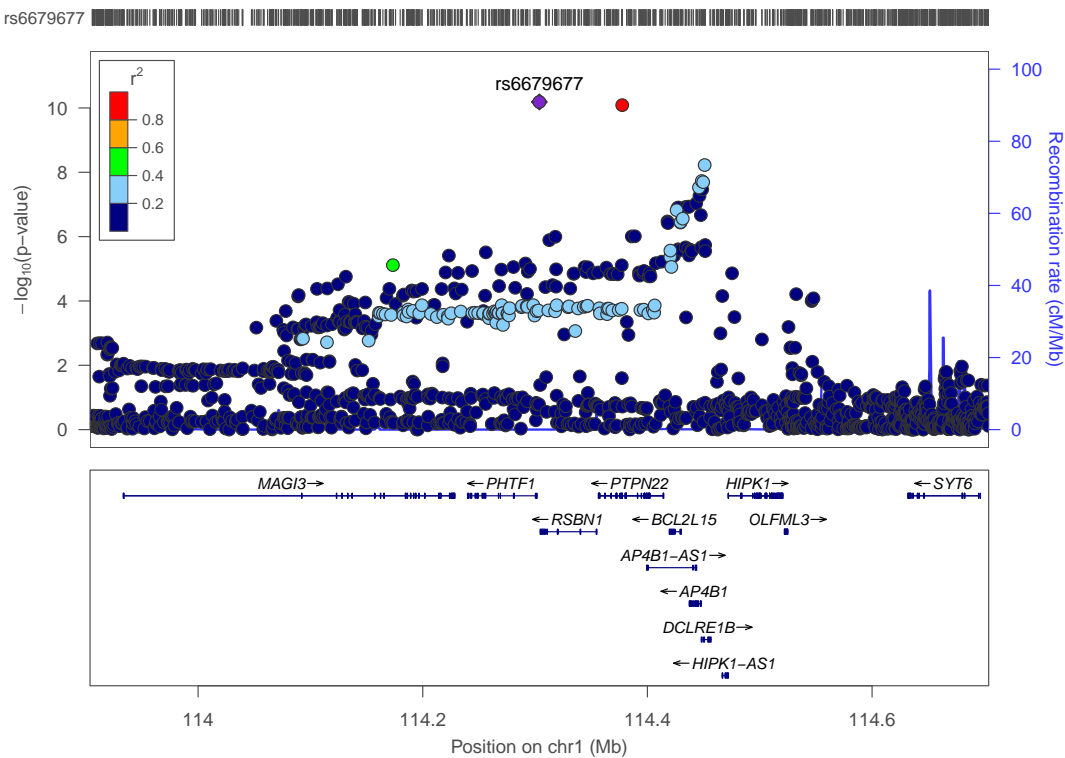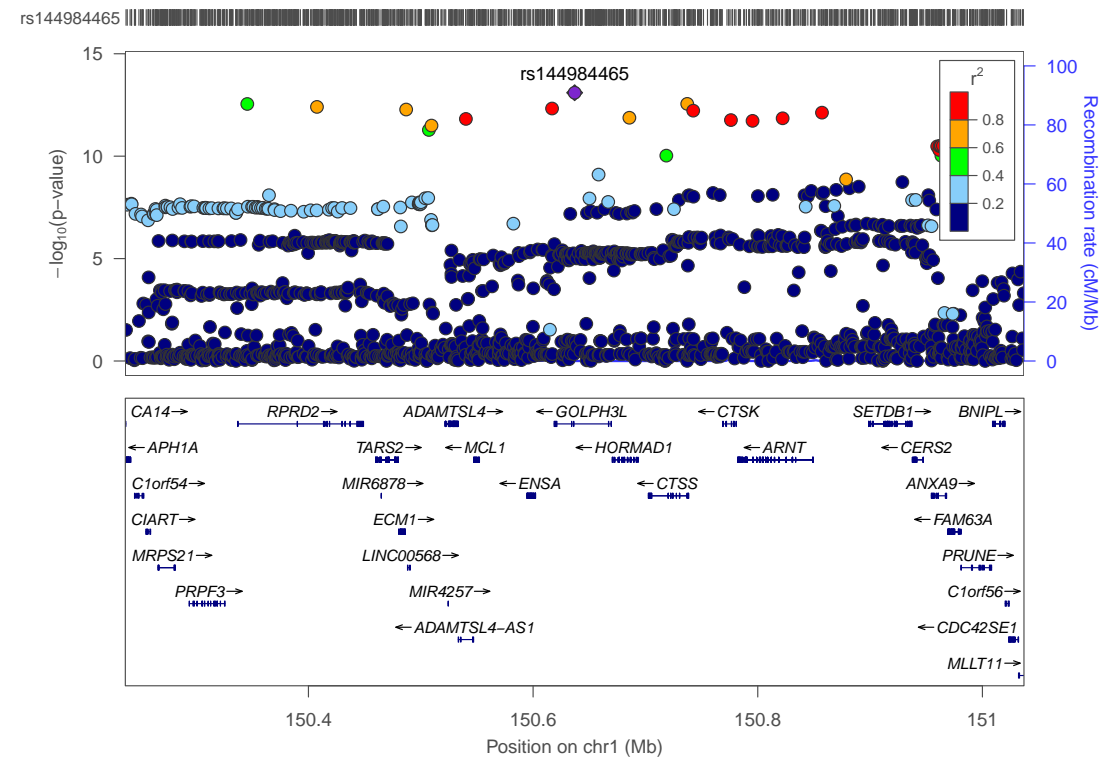

rs1870940

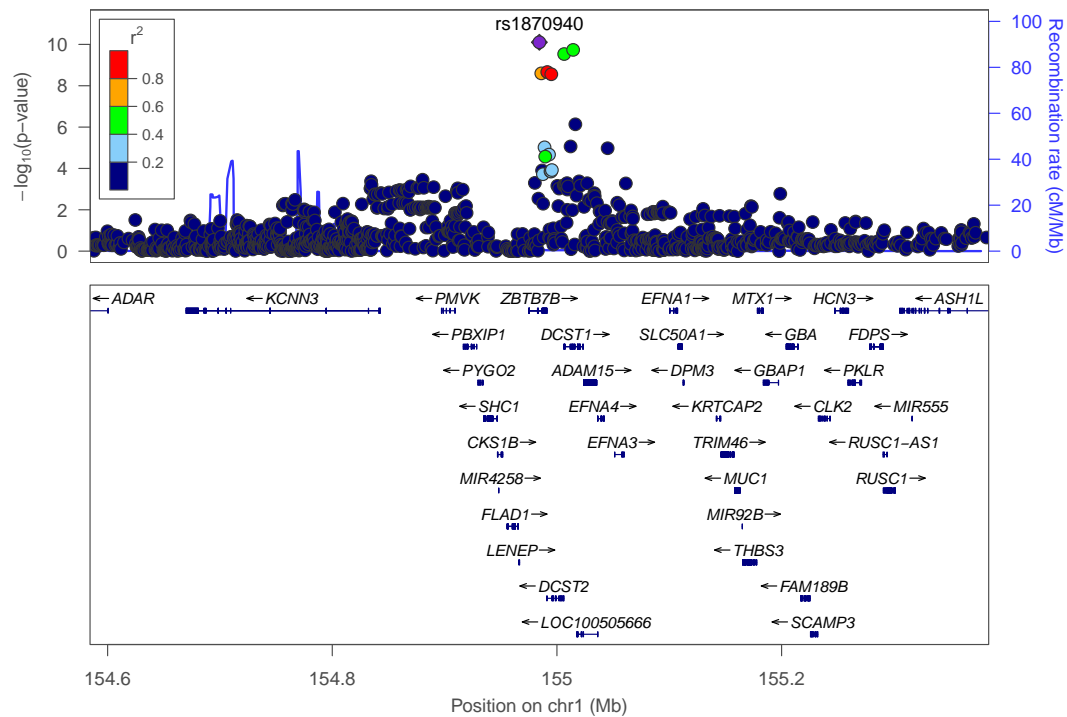

rs12070203

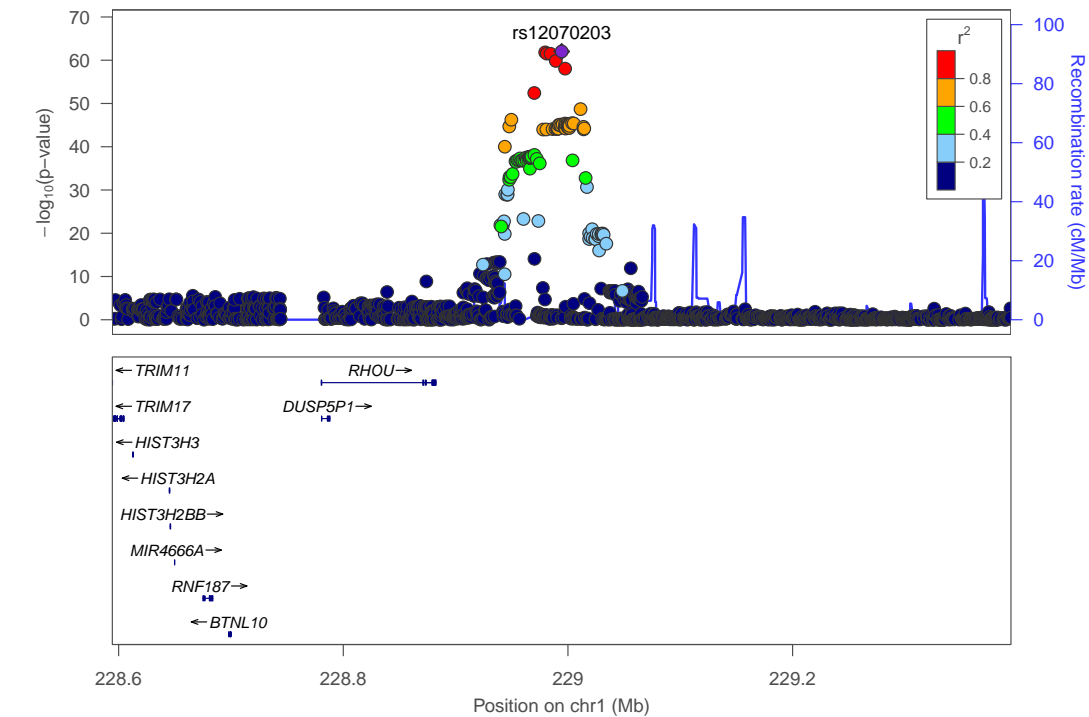

rs4149909

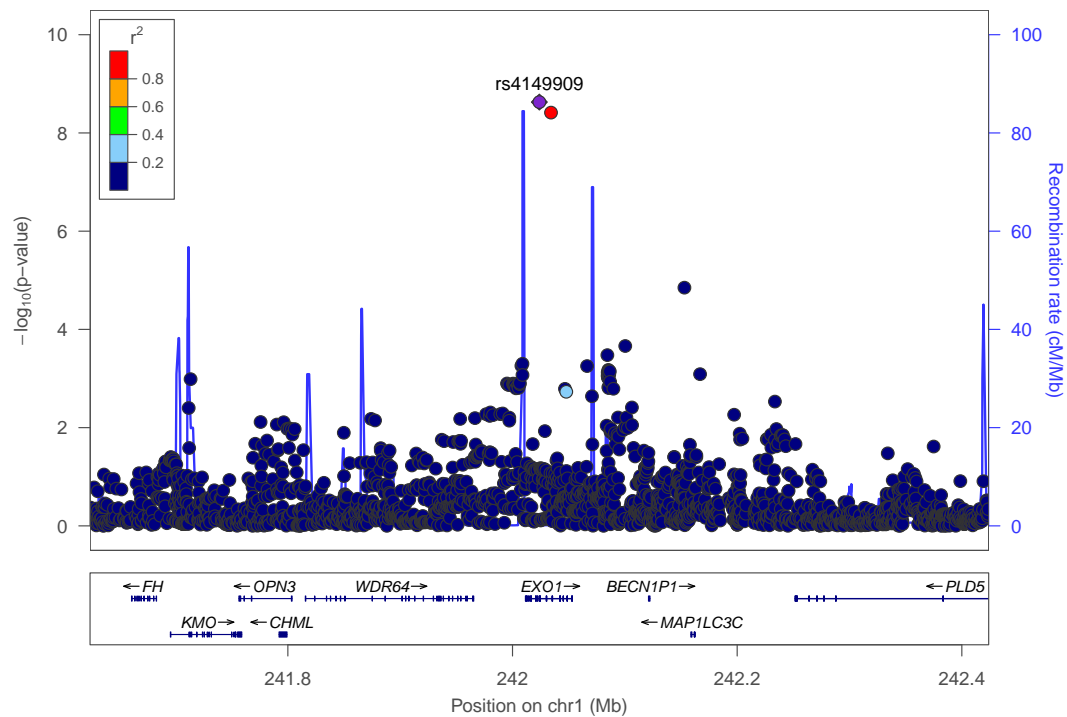

rs62112661

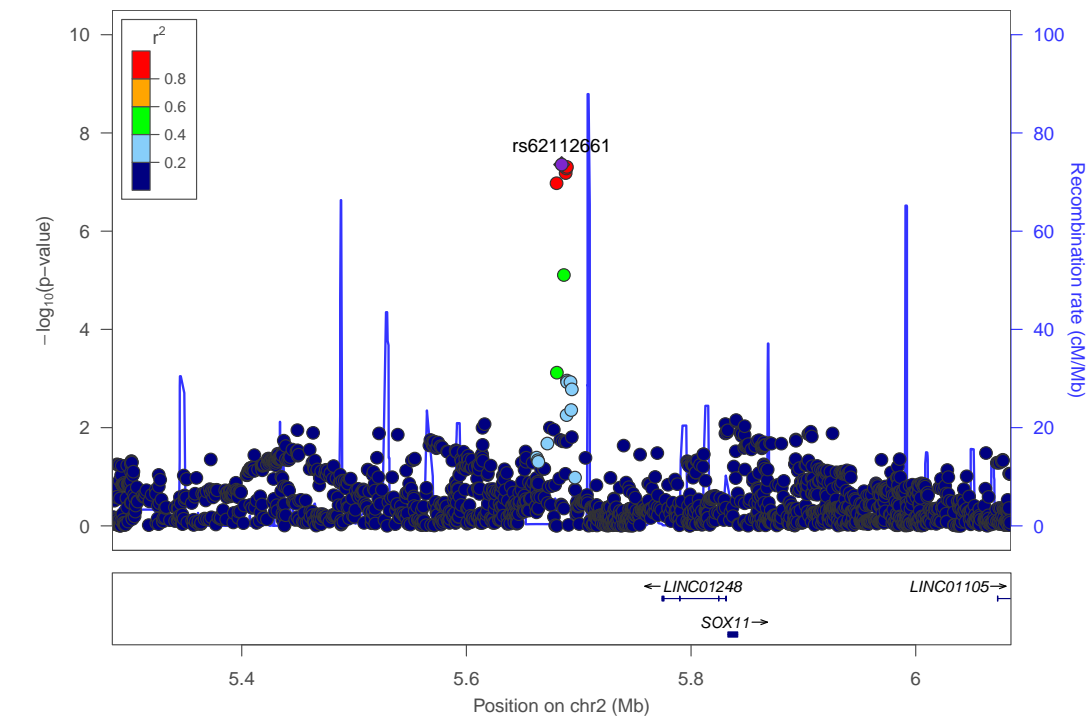

rs79522206

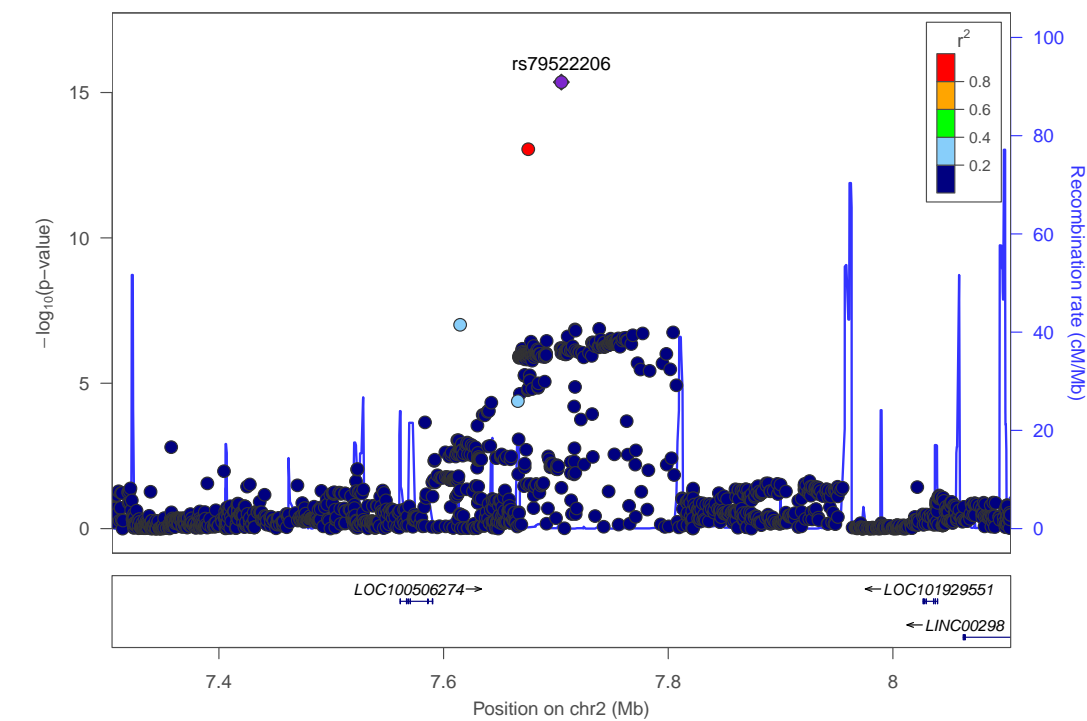

rs6739779

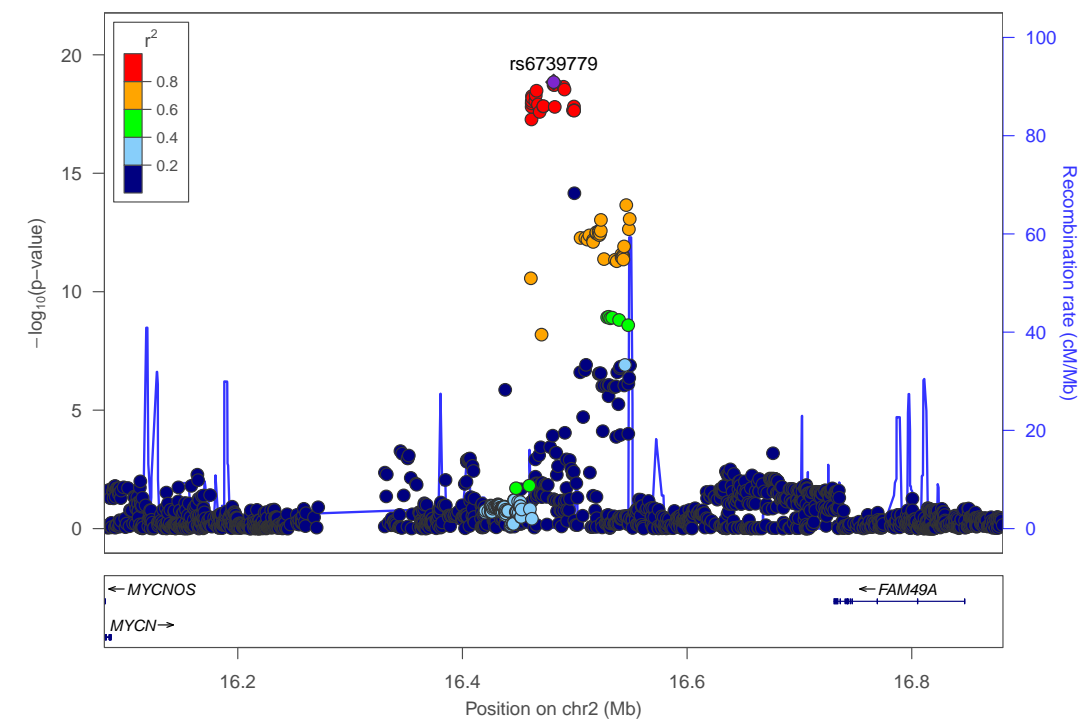

rs3845780

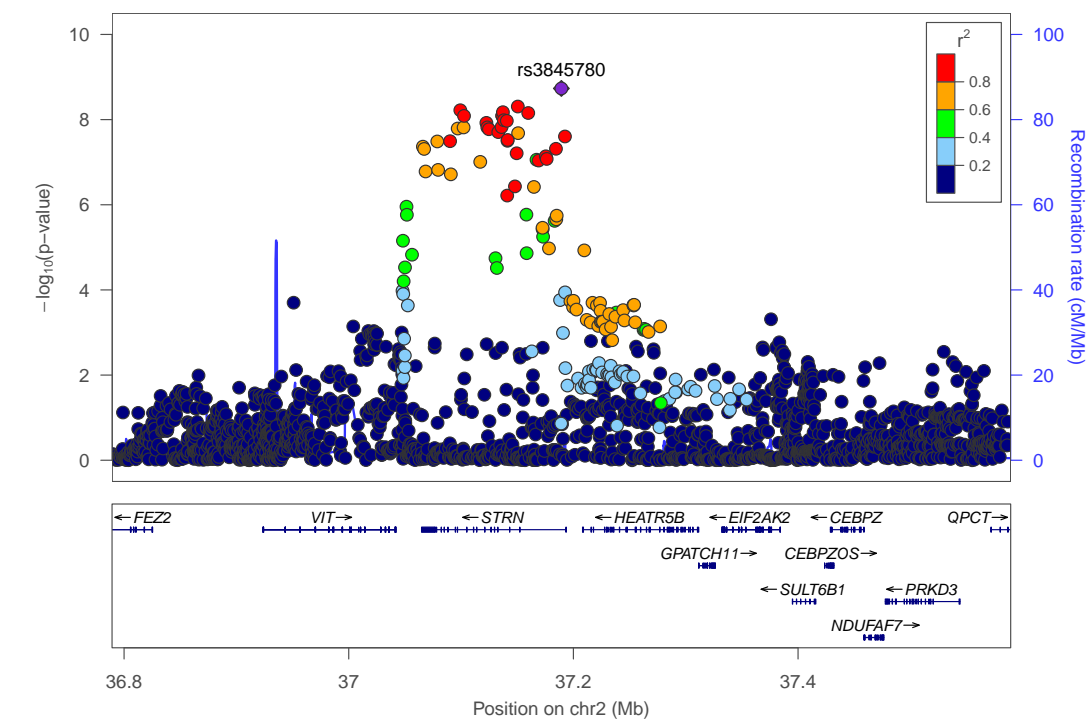

rs1800440

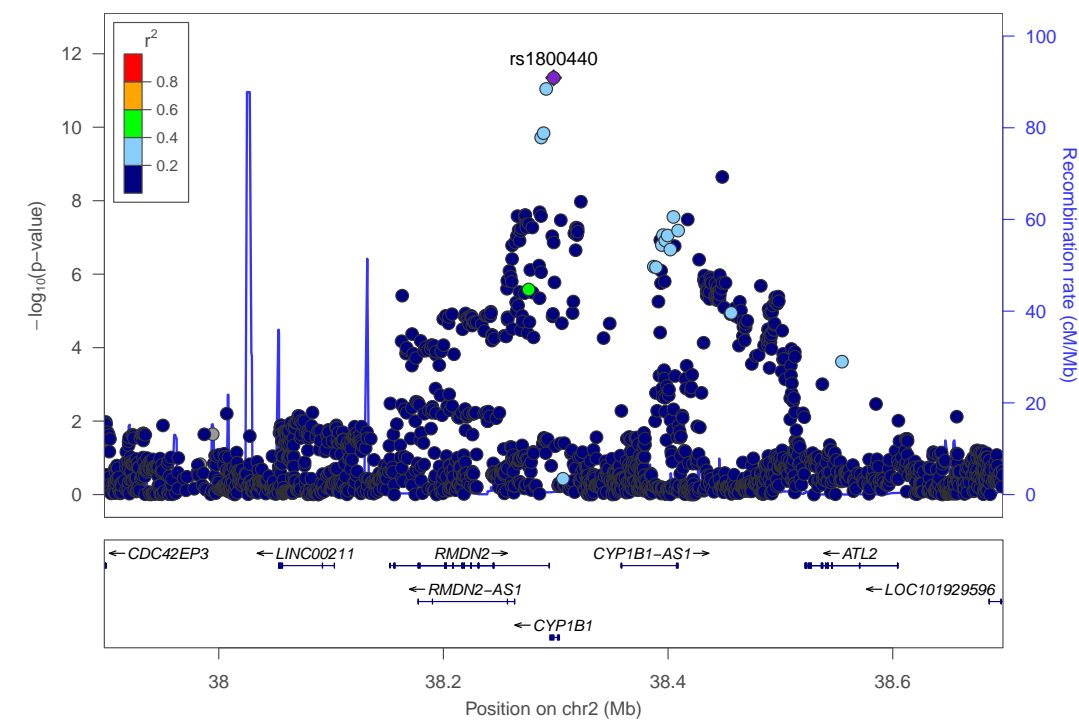

rs6707137

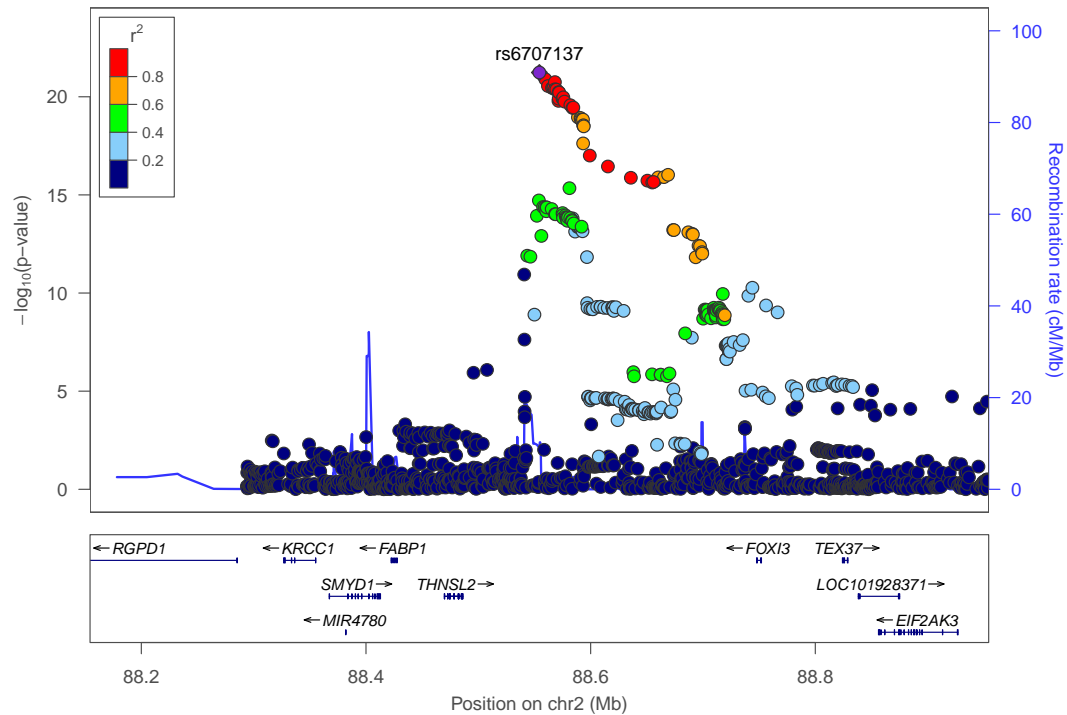

rs6743068

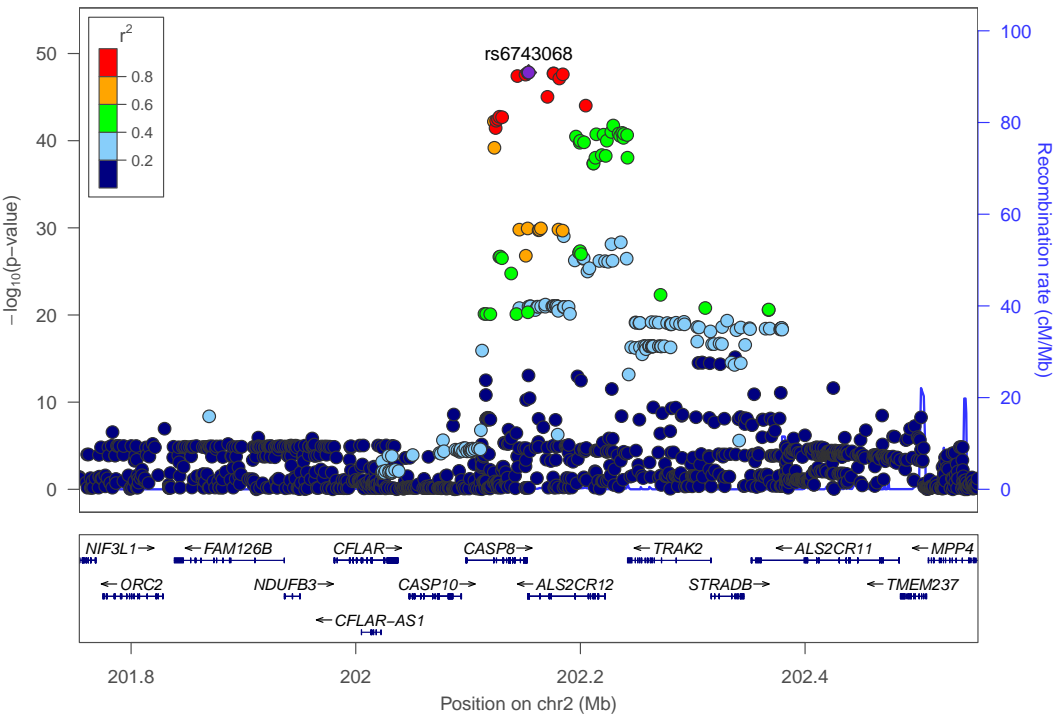

rs231779

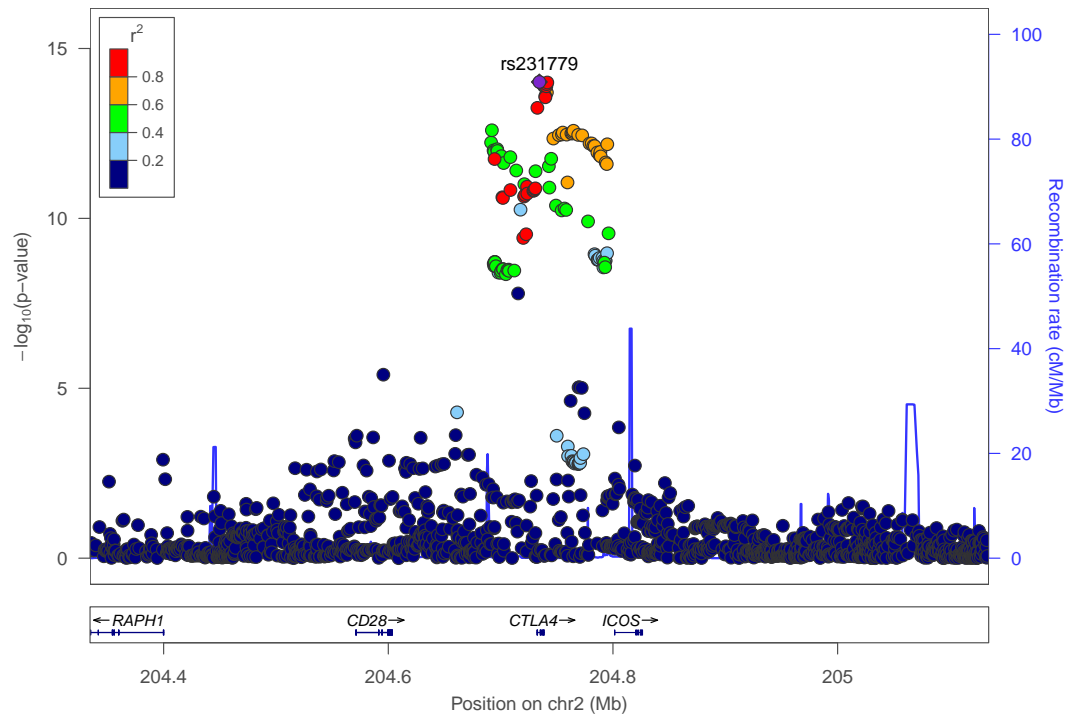

rs11707890

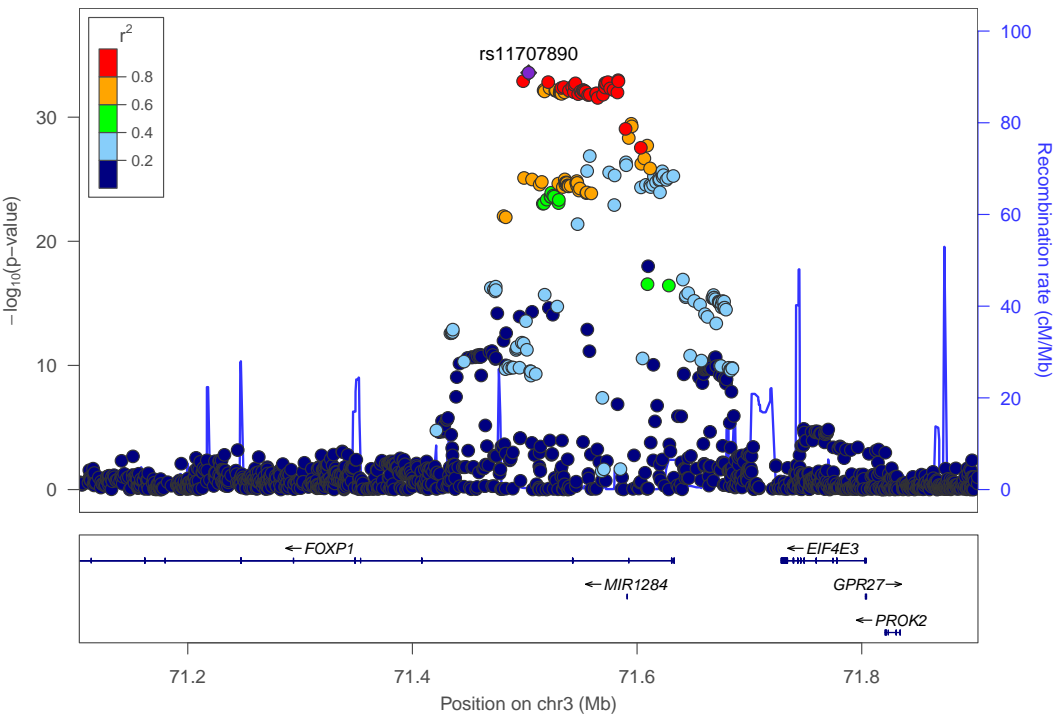

rs7620634

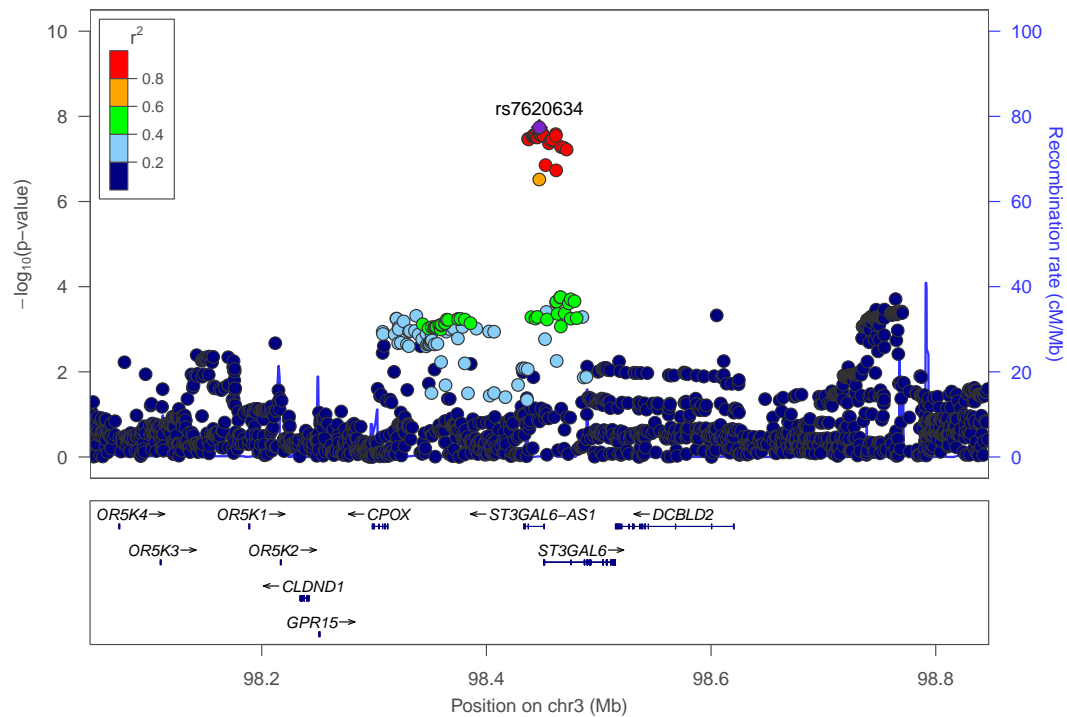

rs1915930

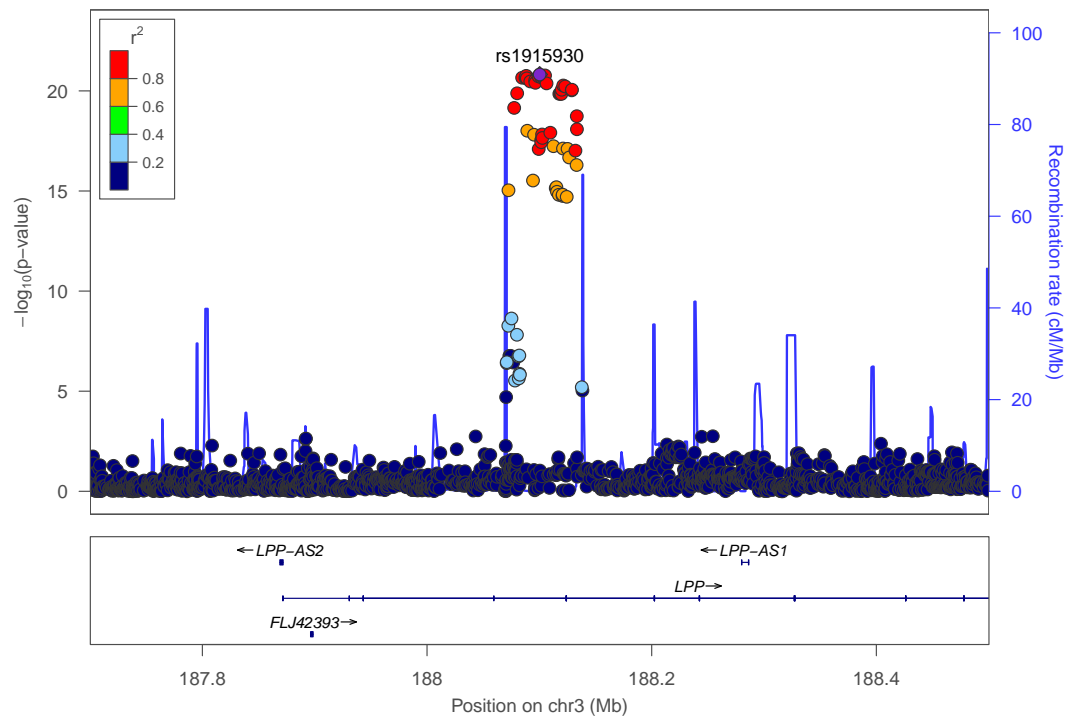

rs2853677

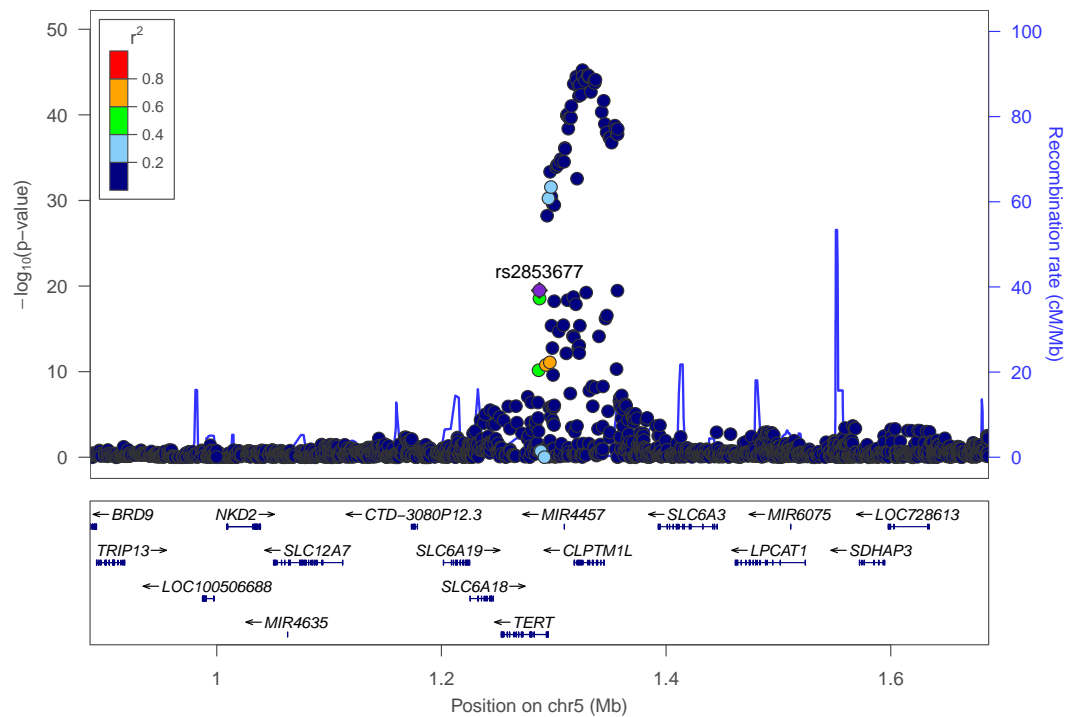

rs421284

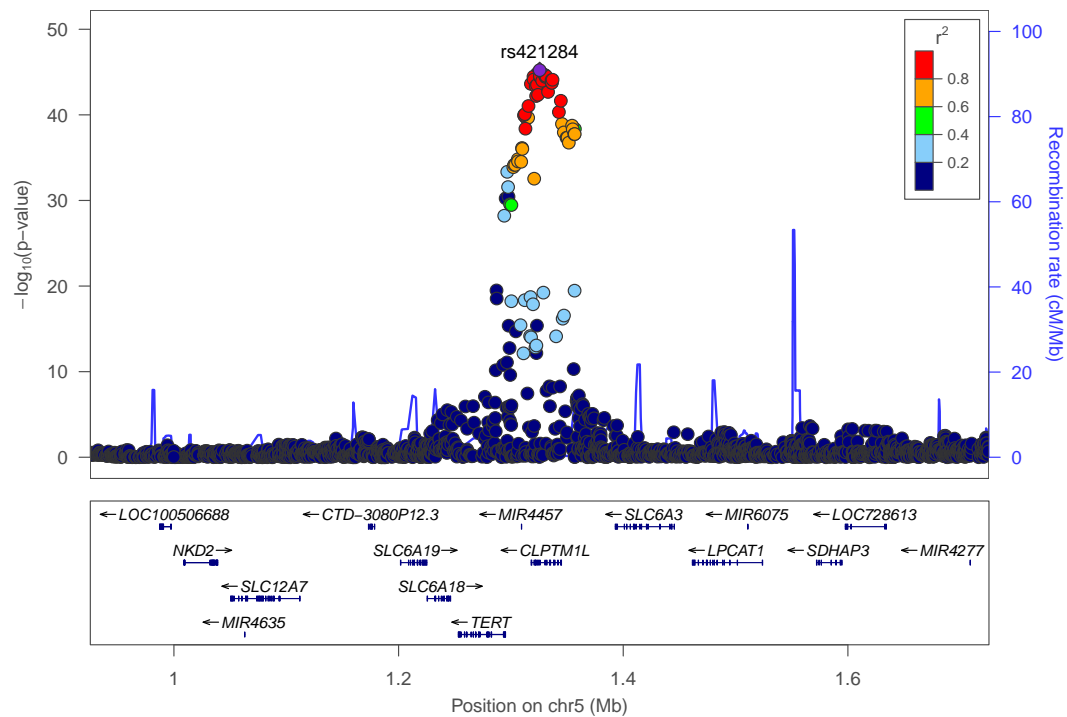

rs35407

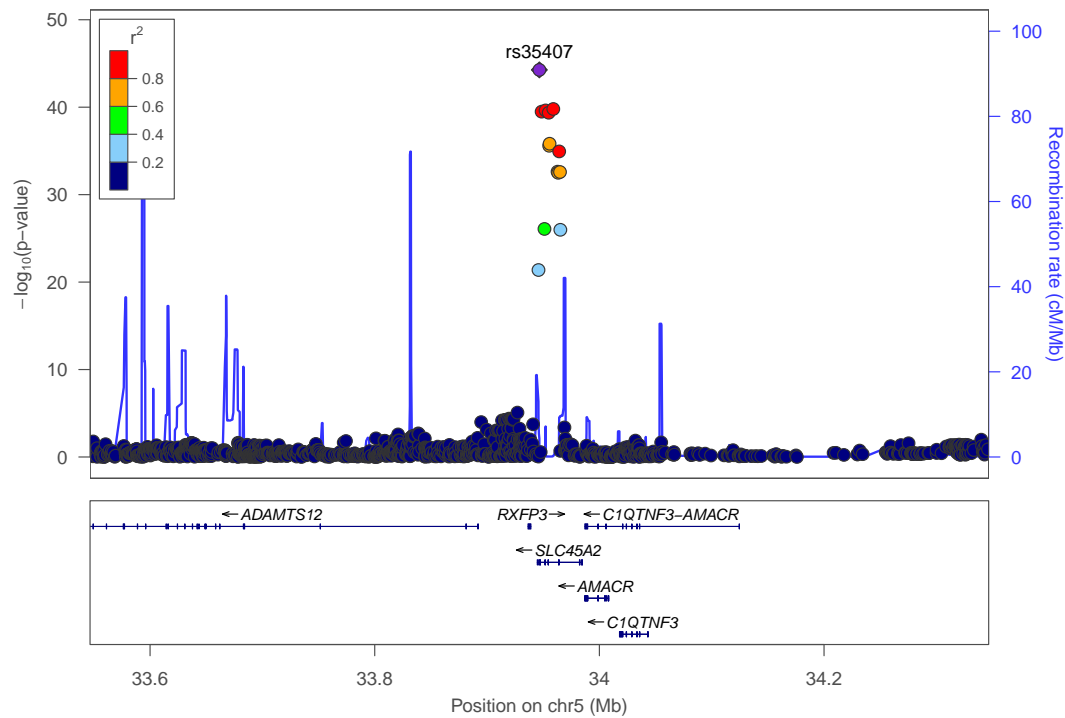

rs17110447

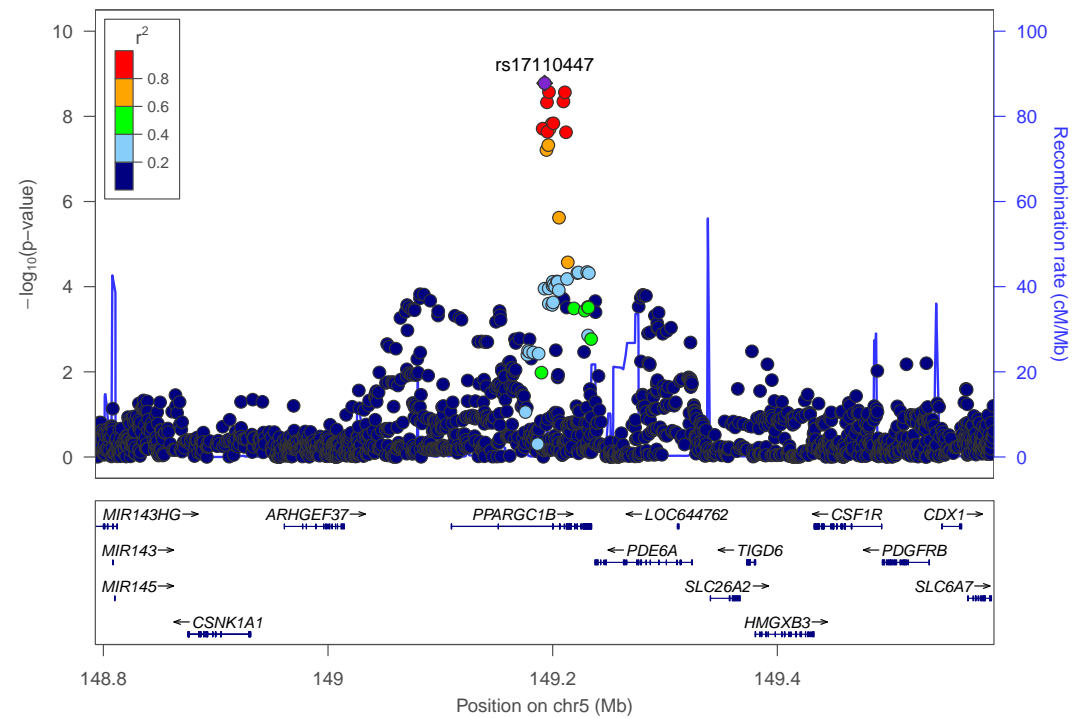

rs12203592

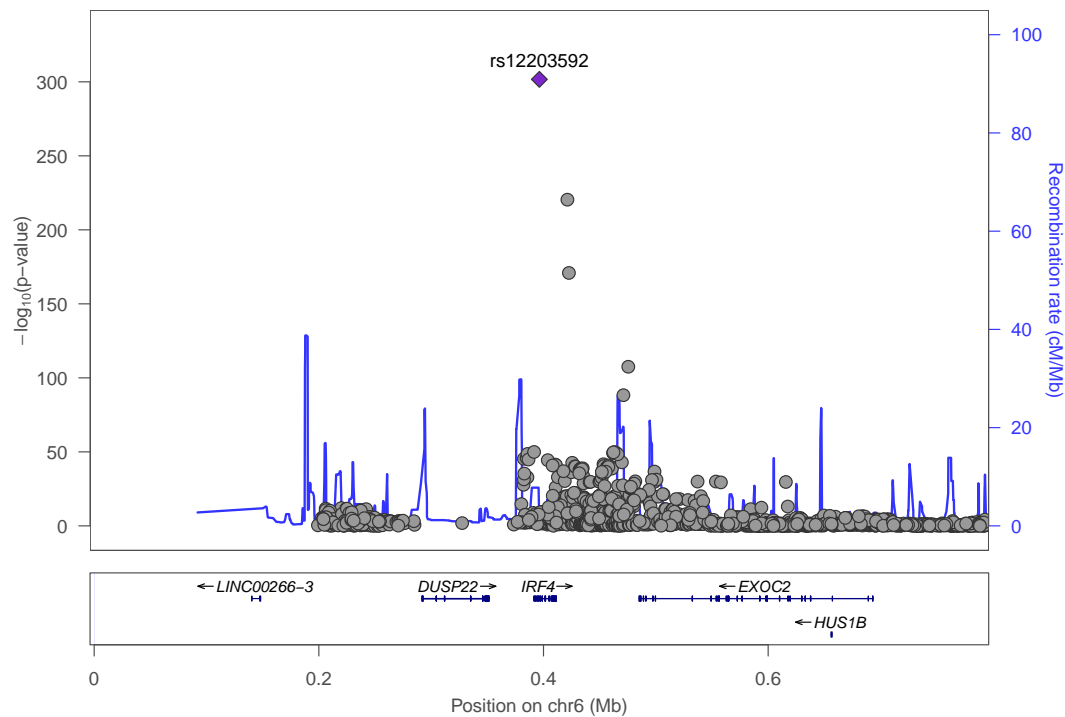

rs1246946

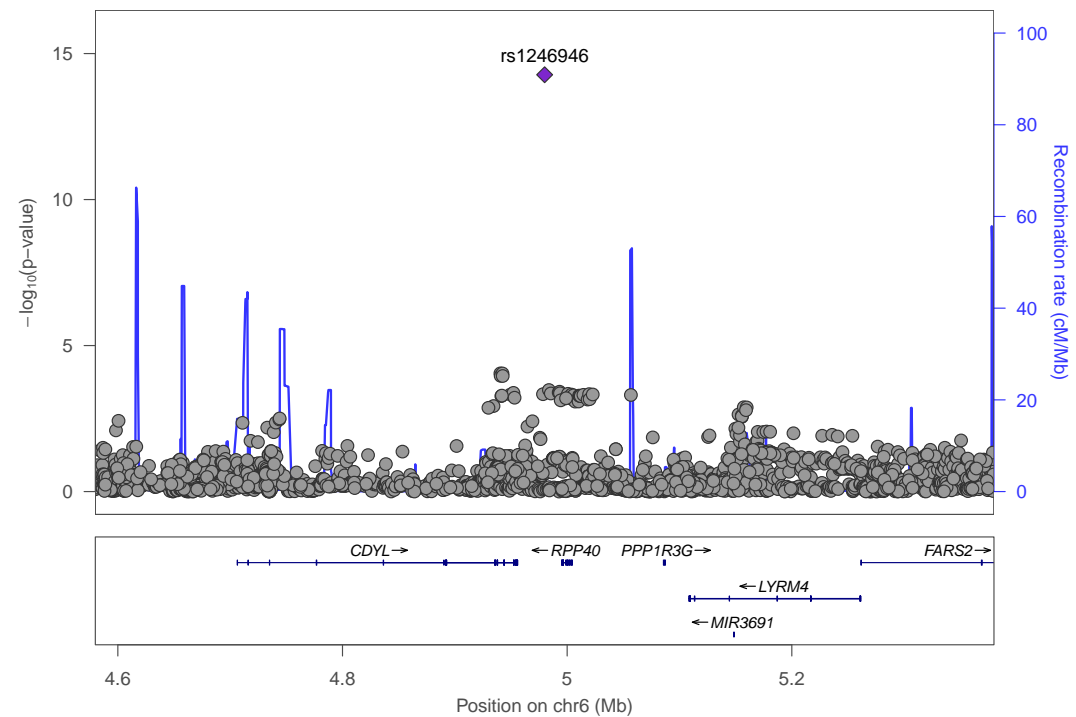

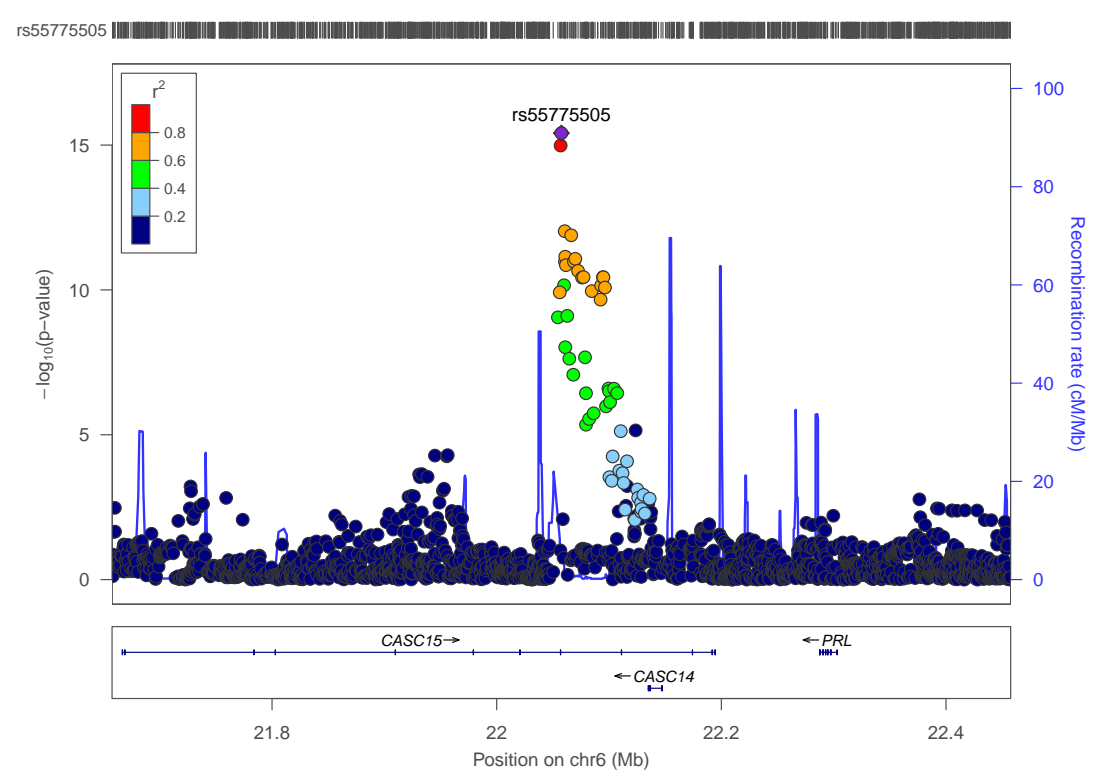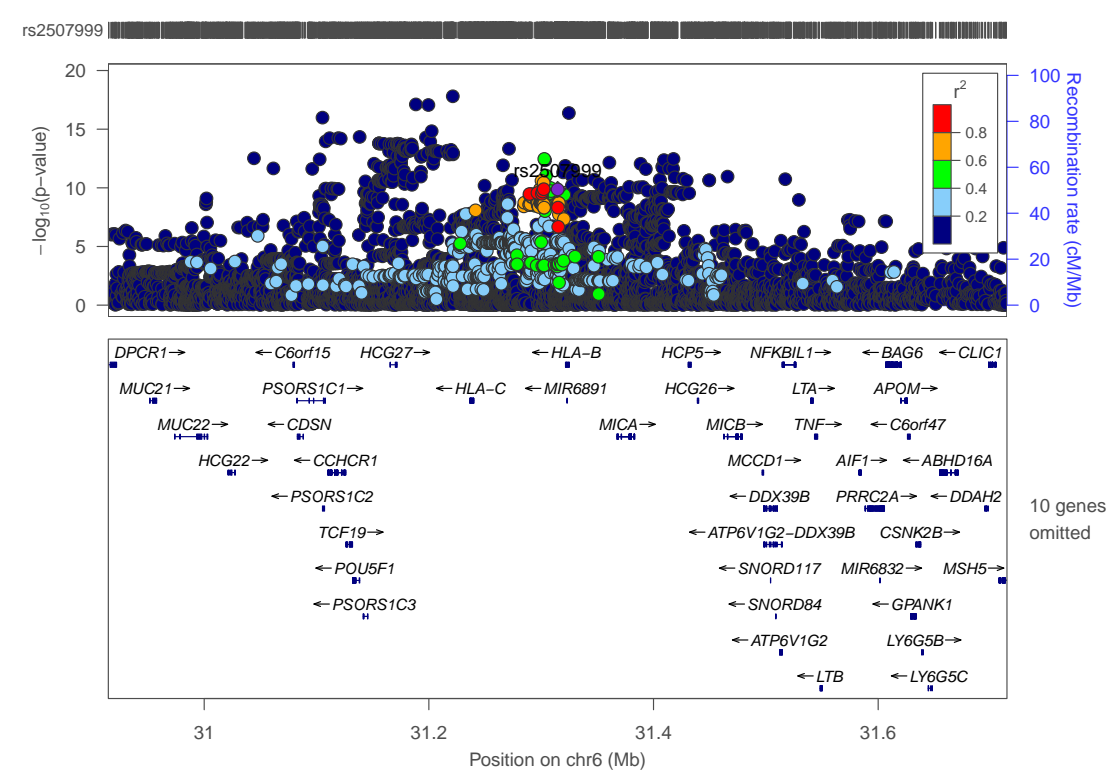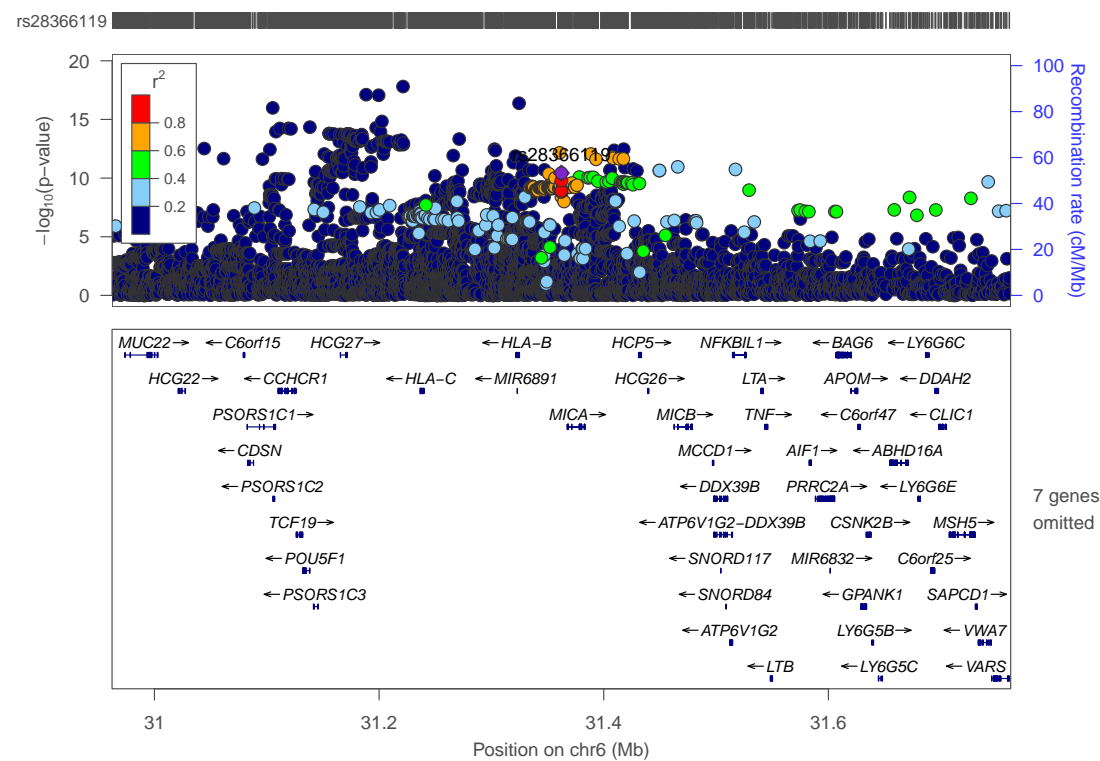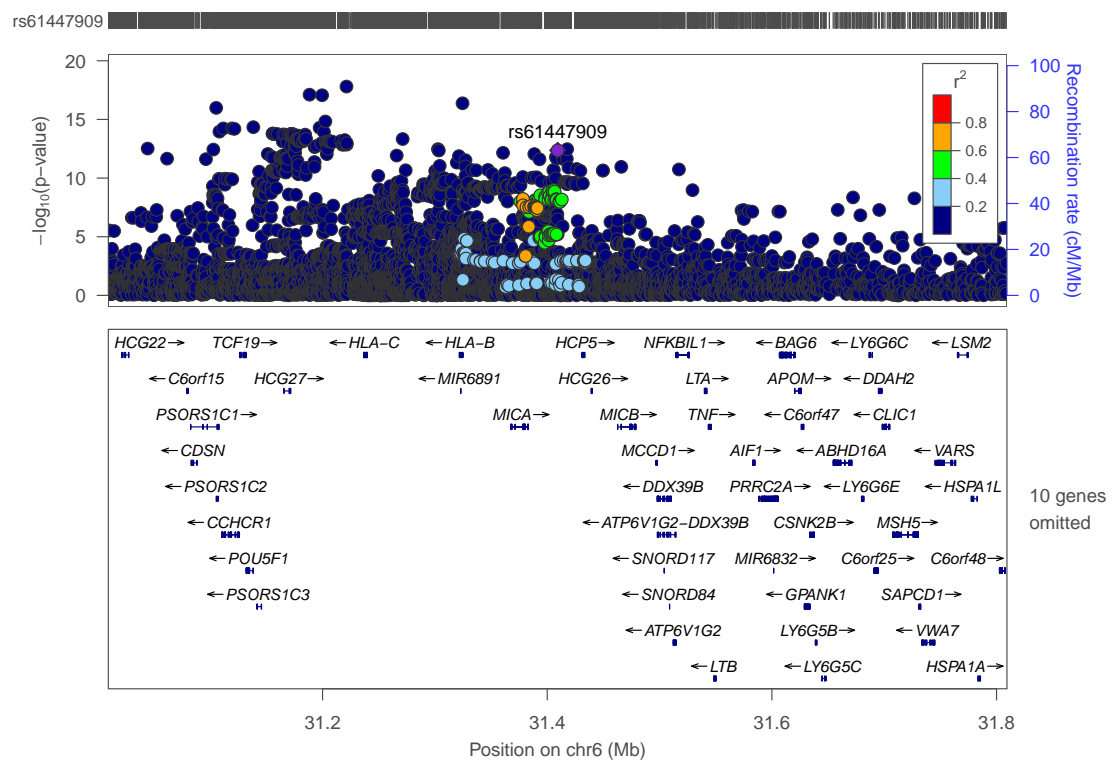

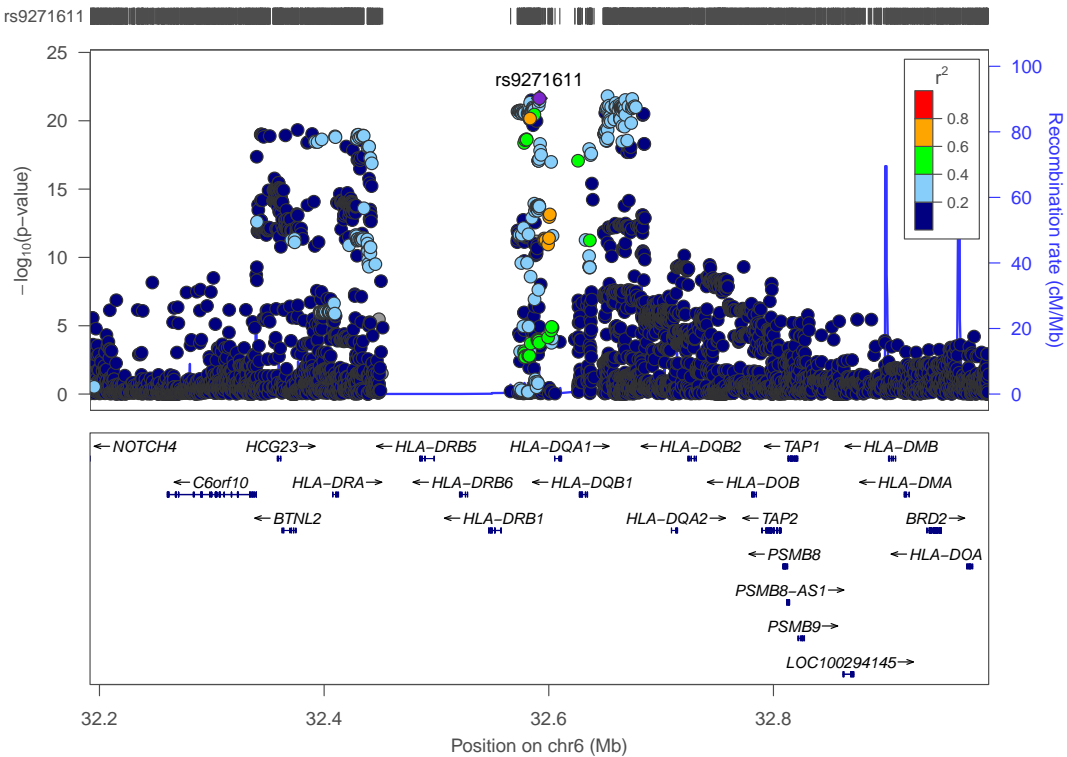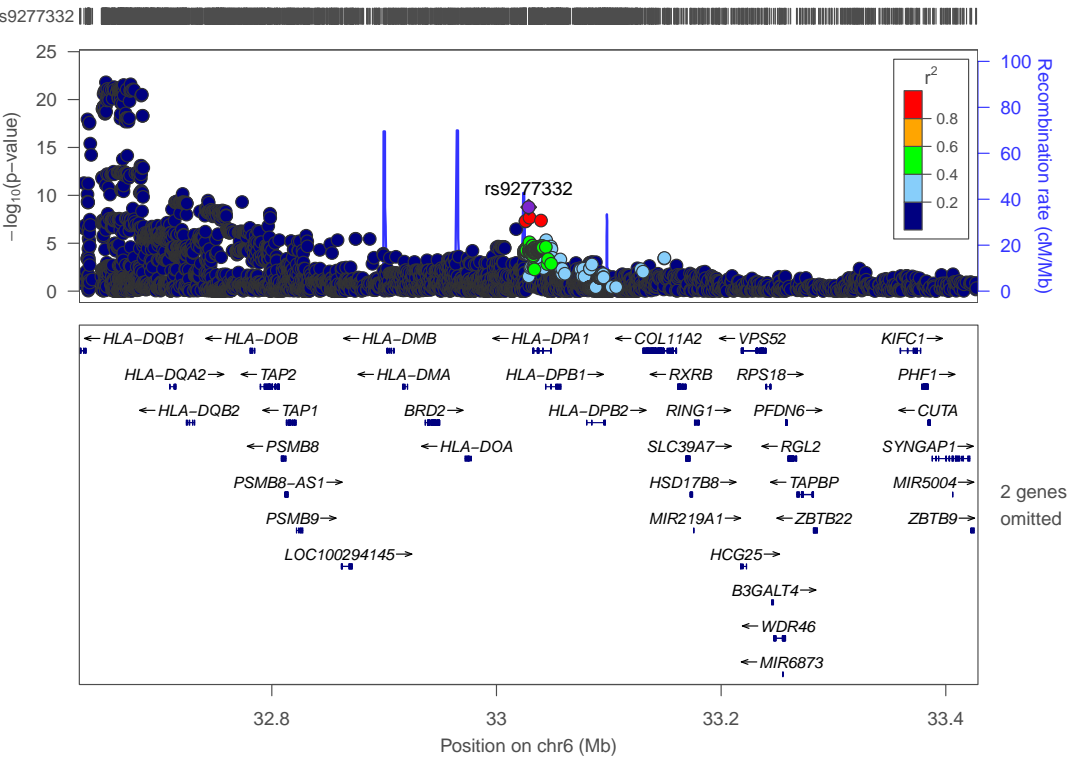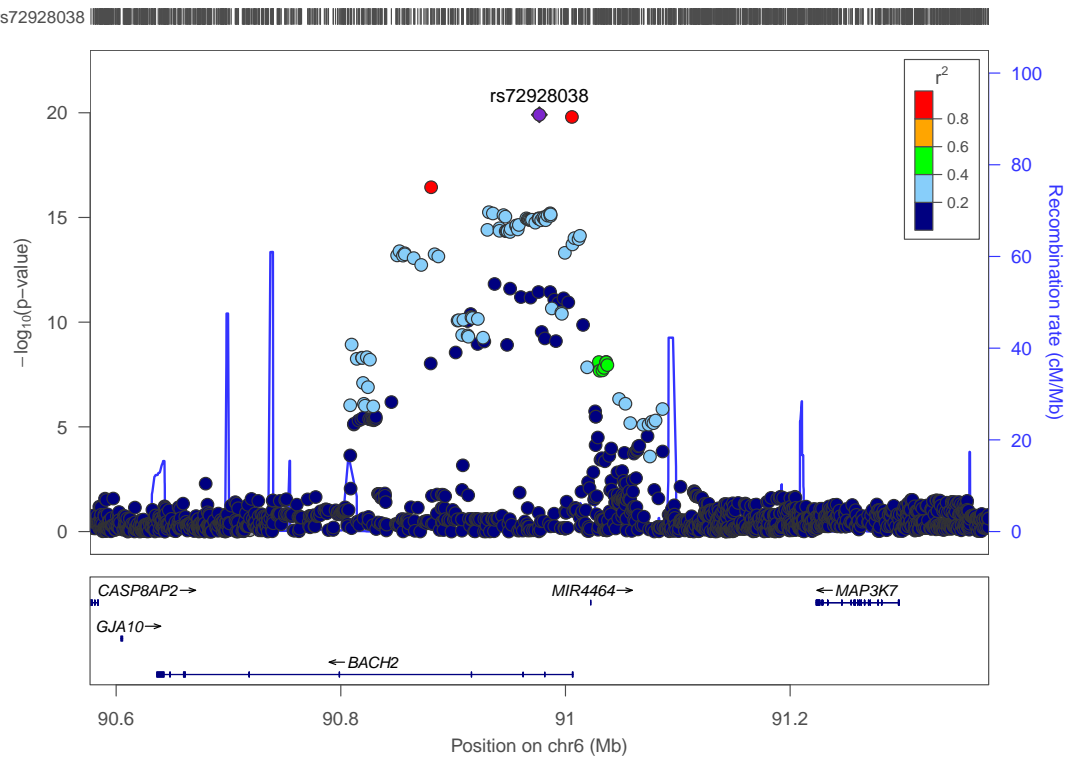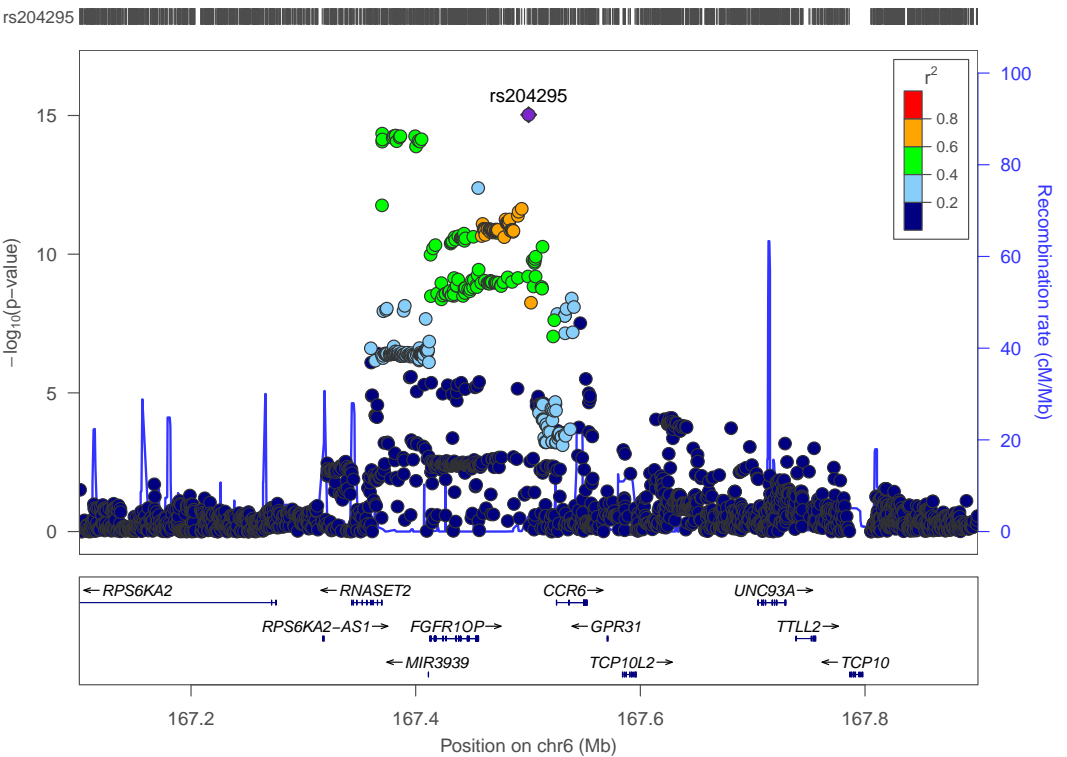

rs836489

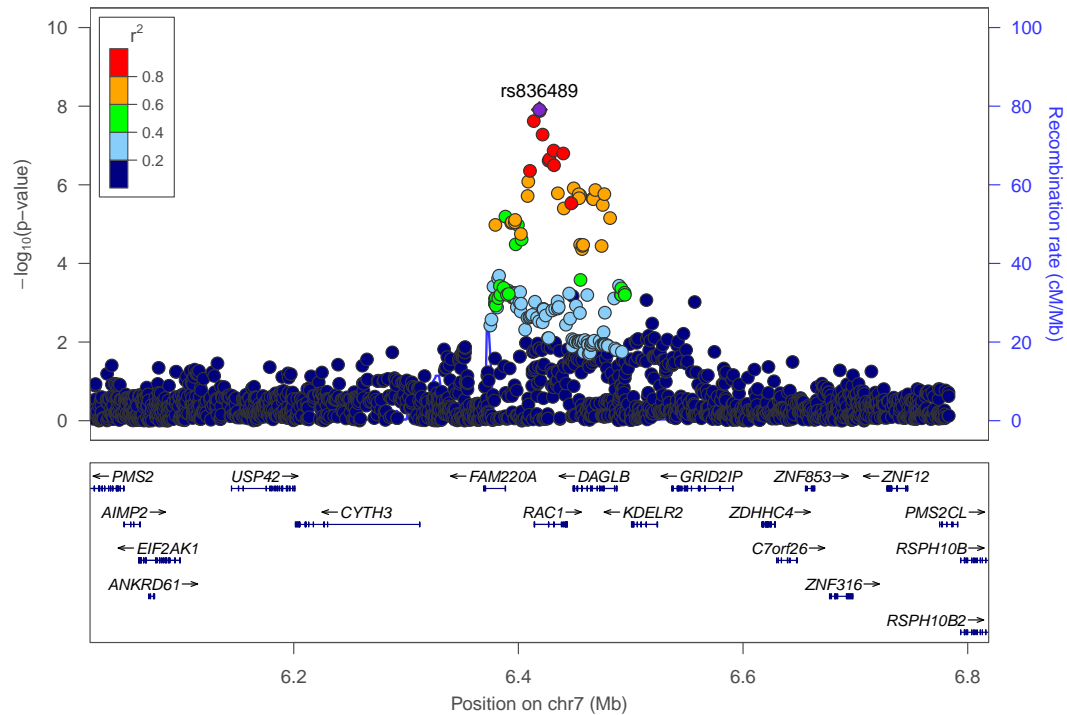

rs76128631

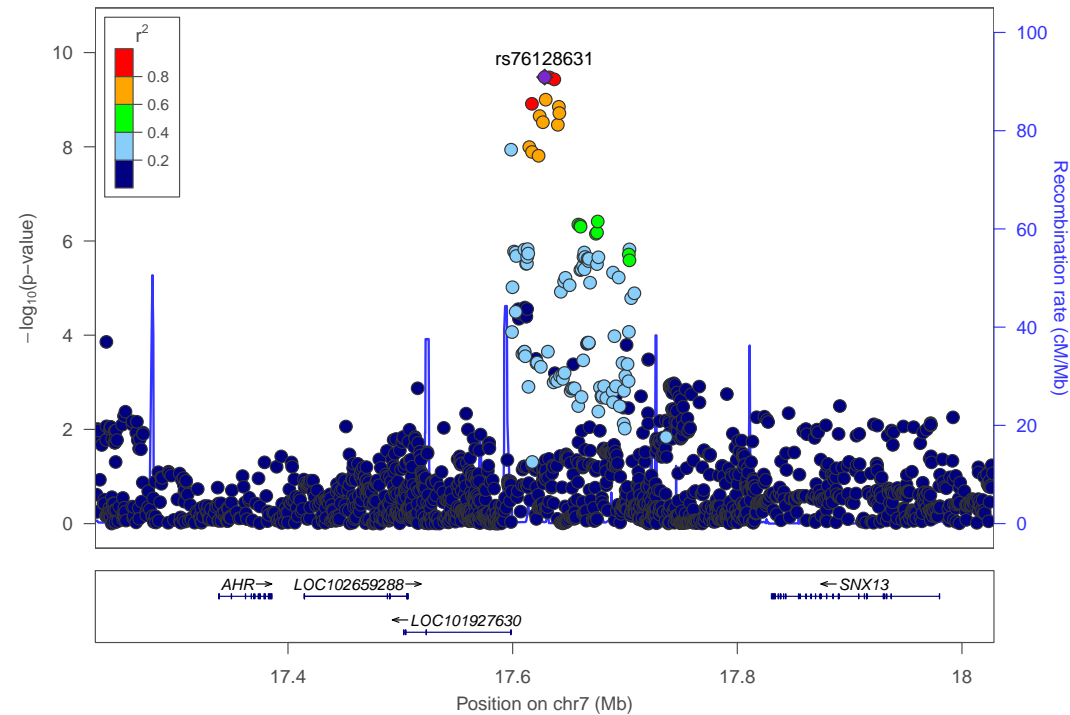

rs117744081

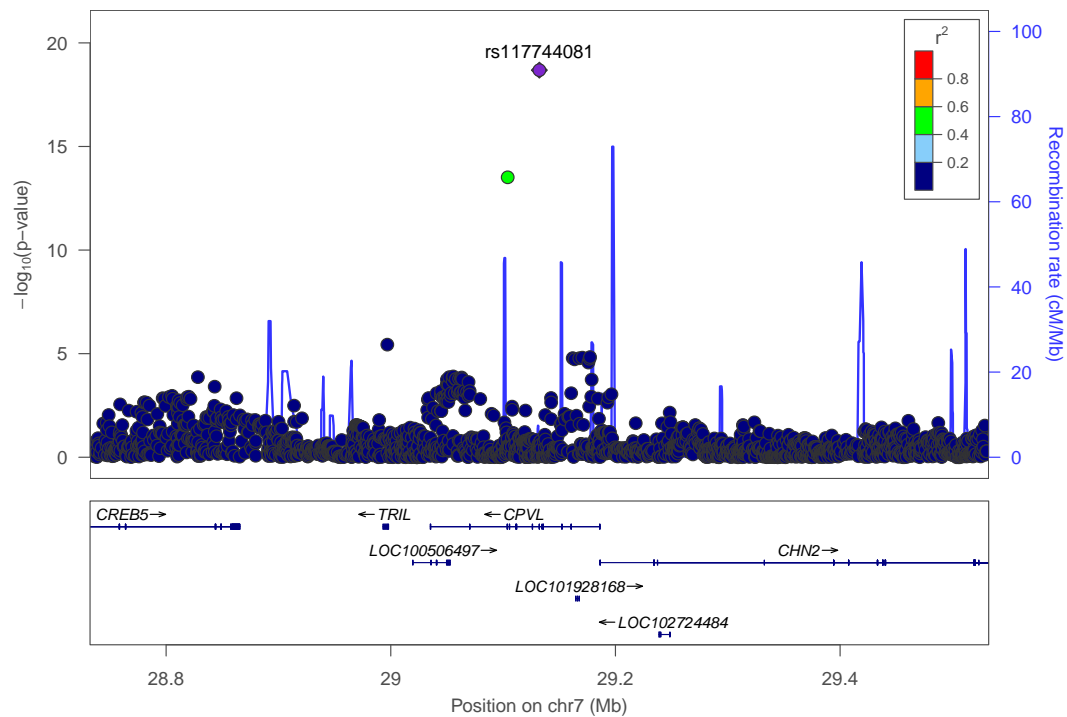

rs700755

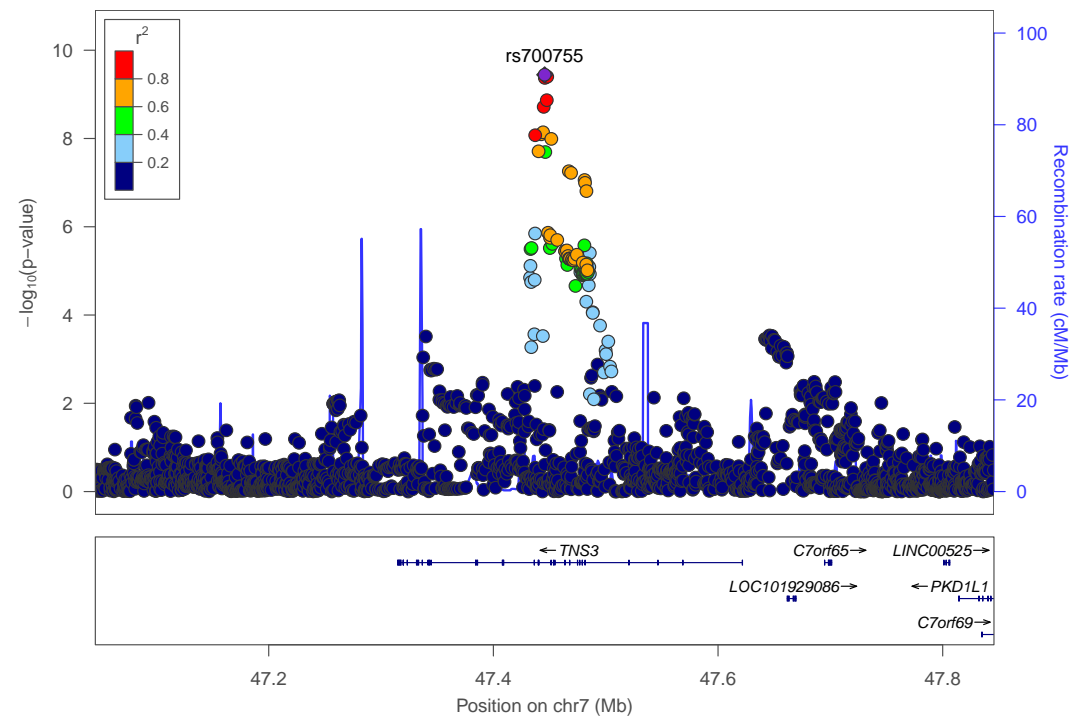

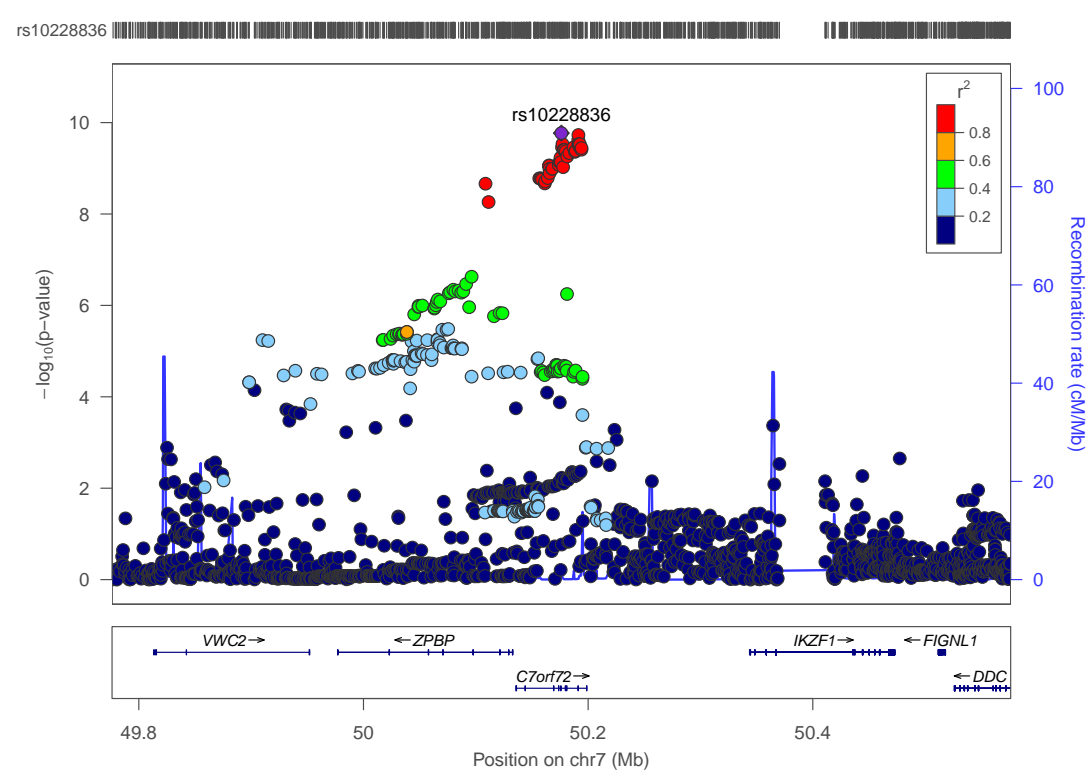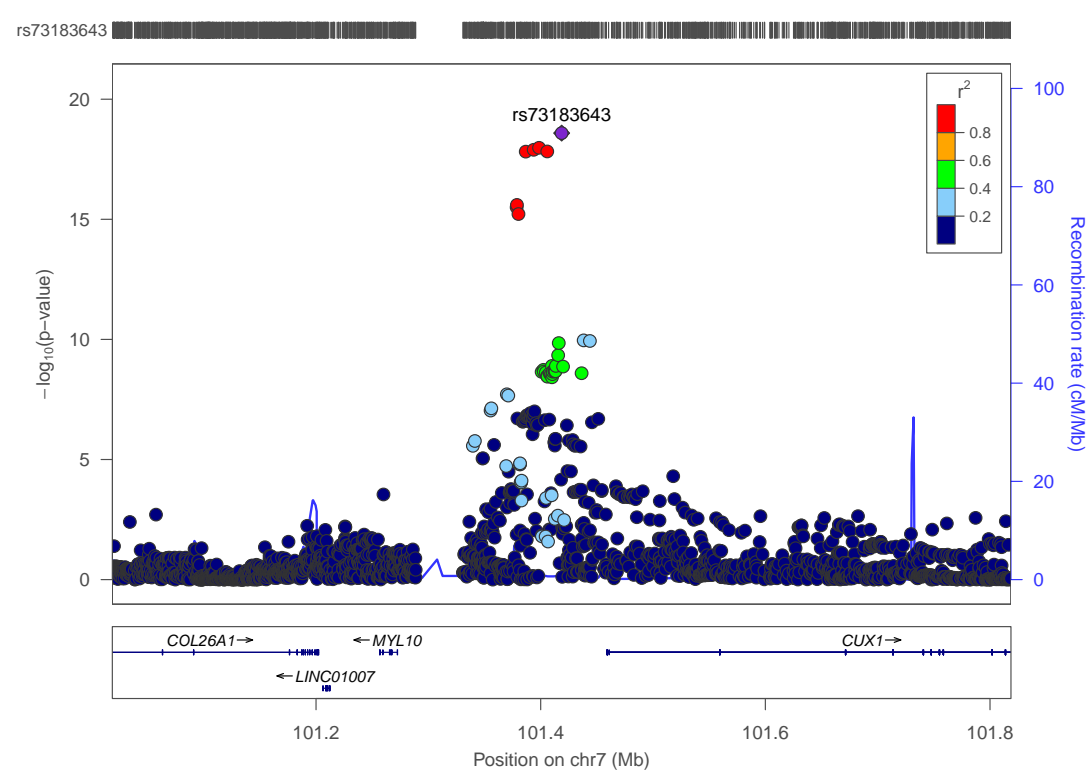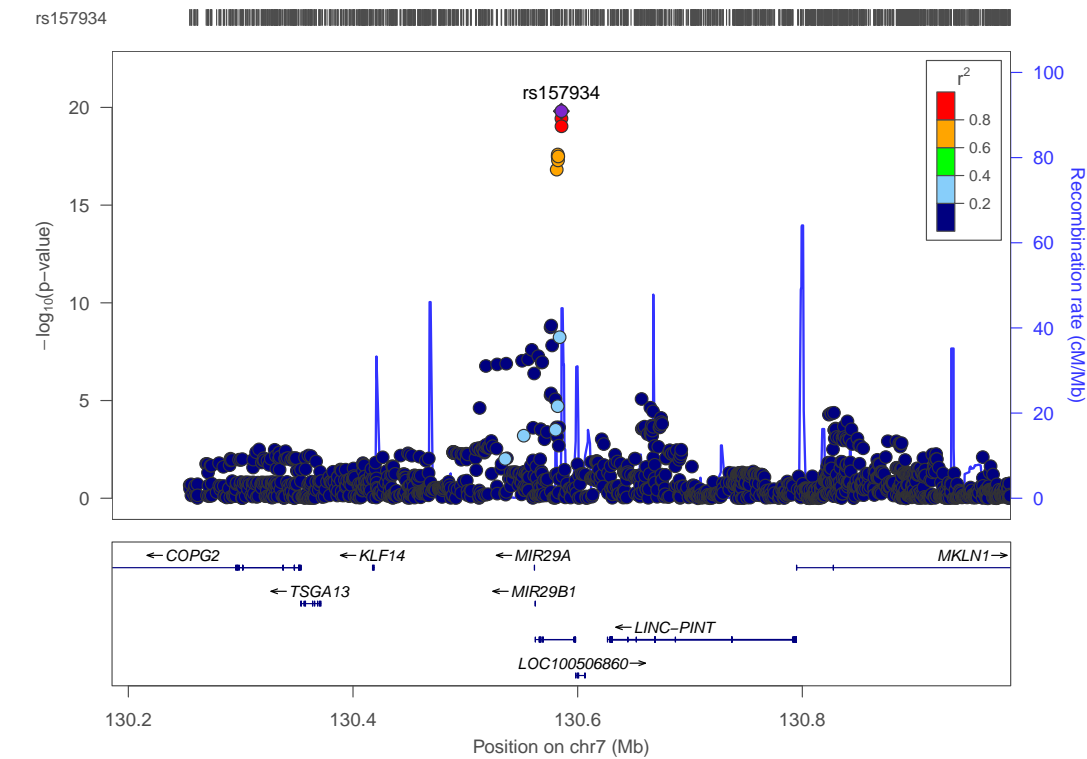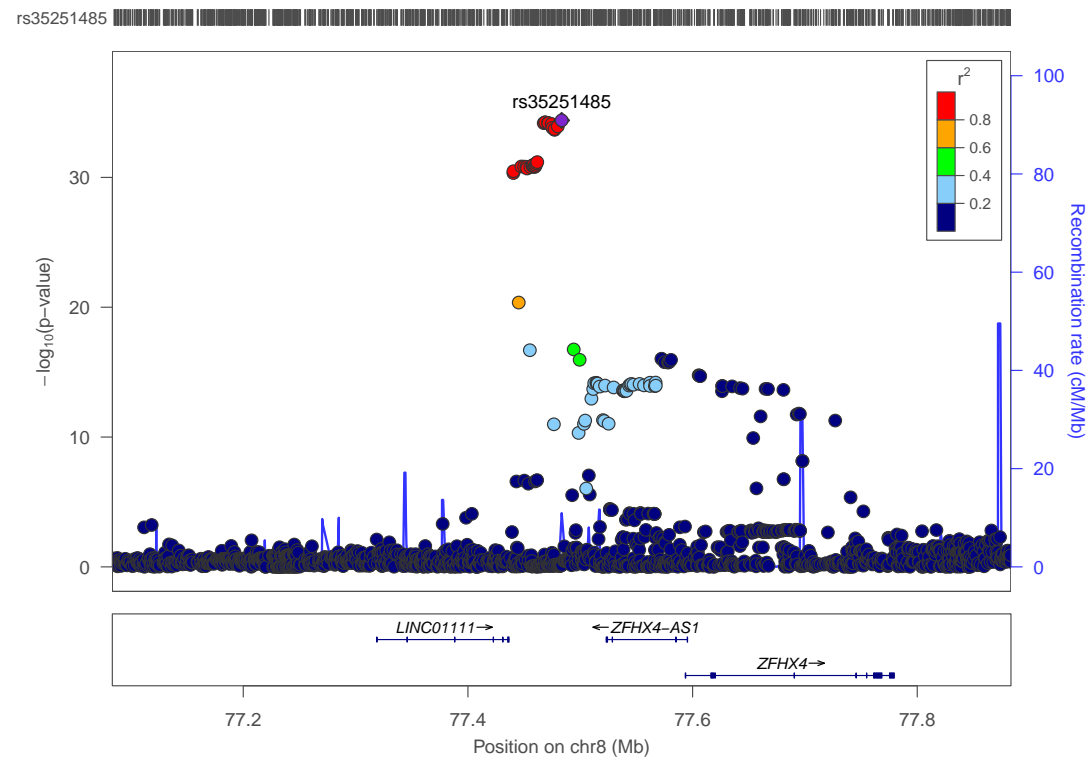

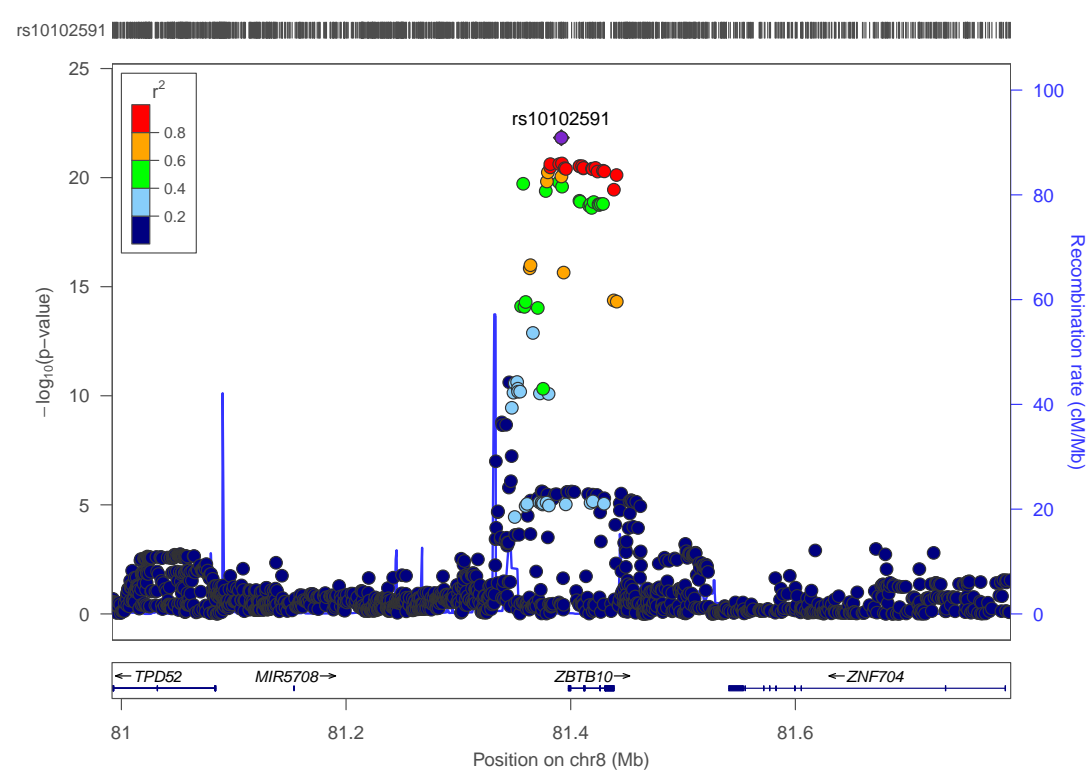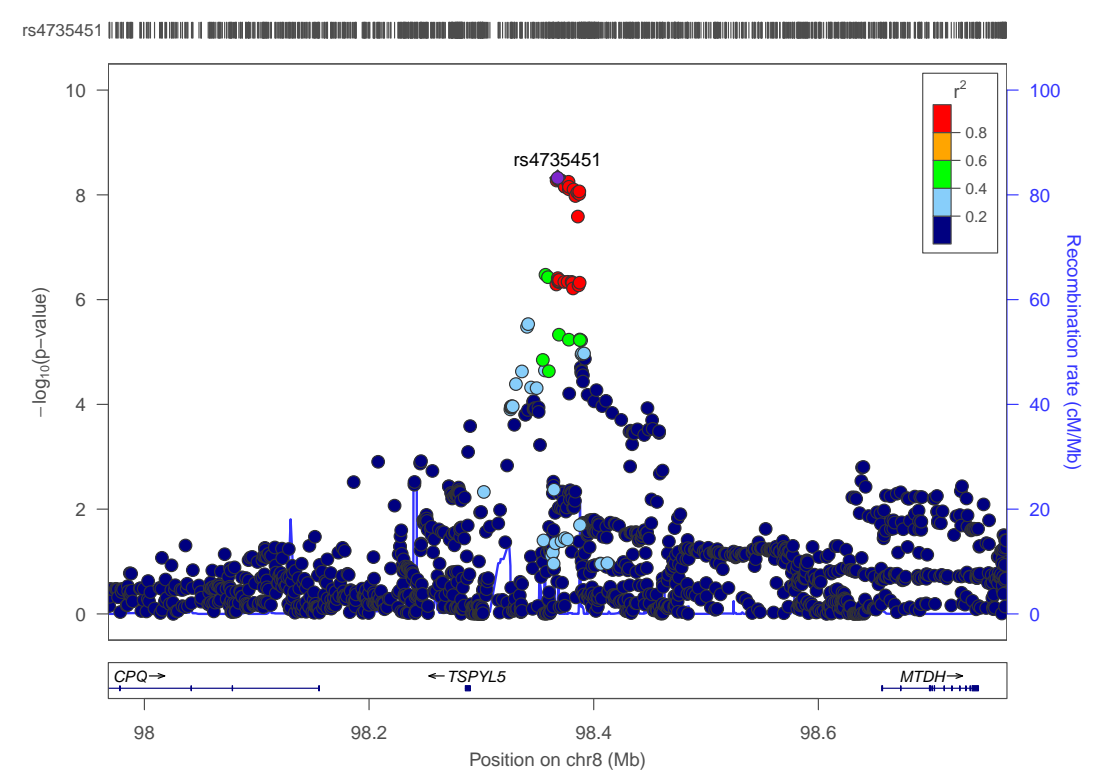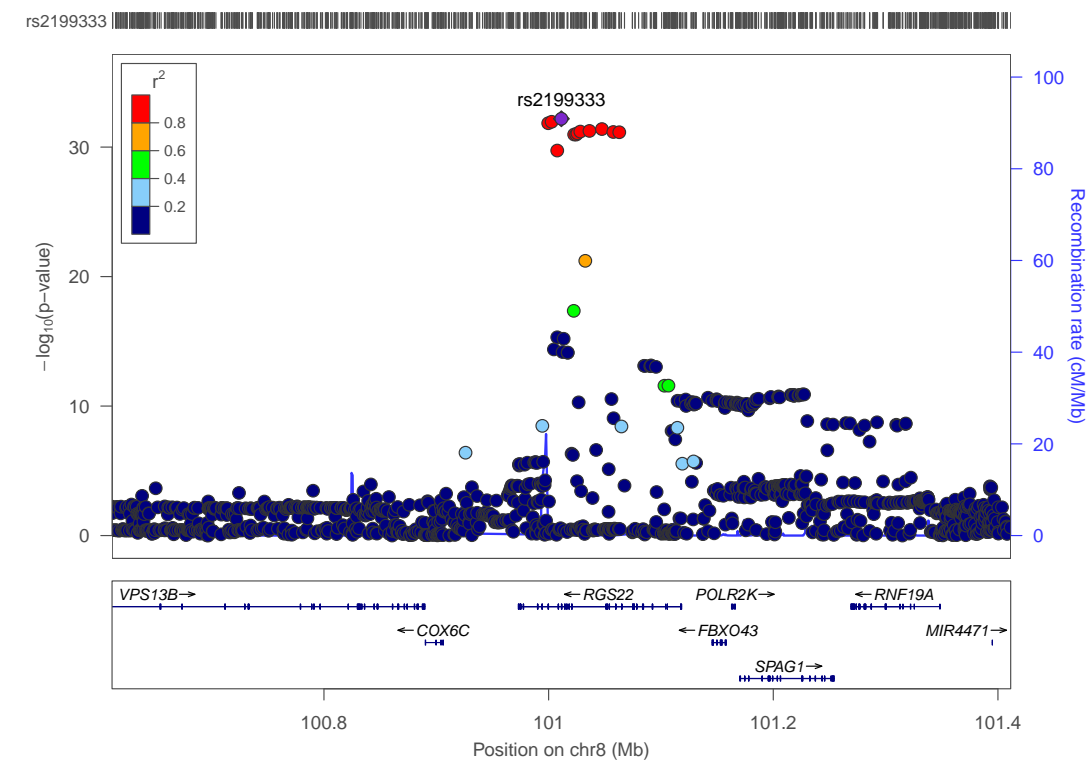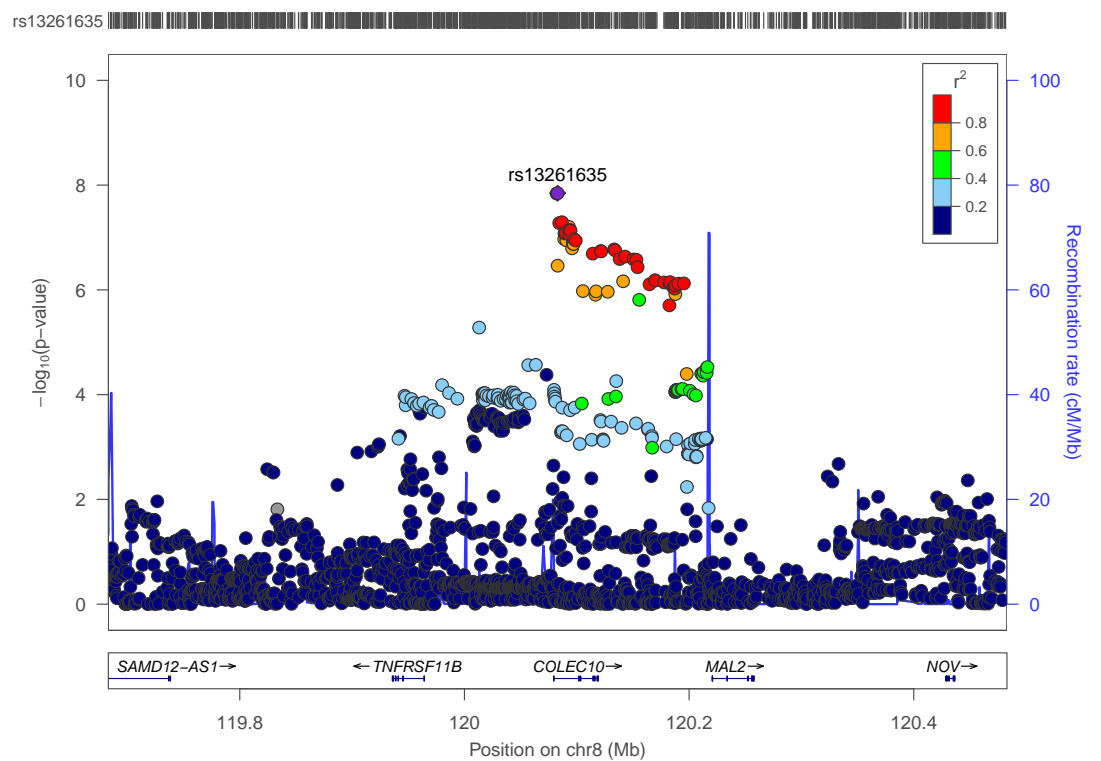

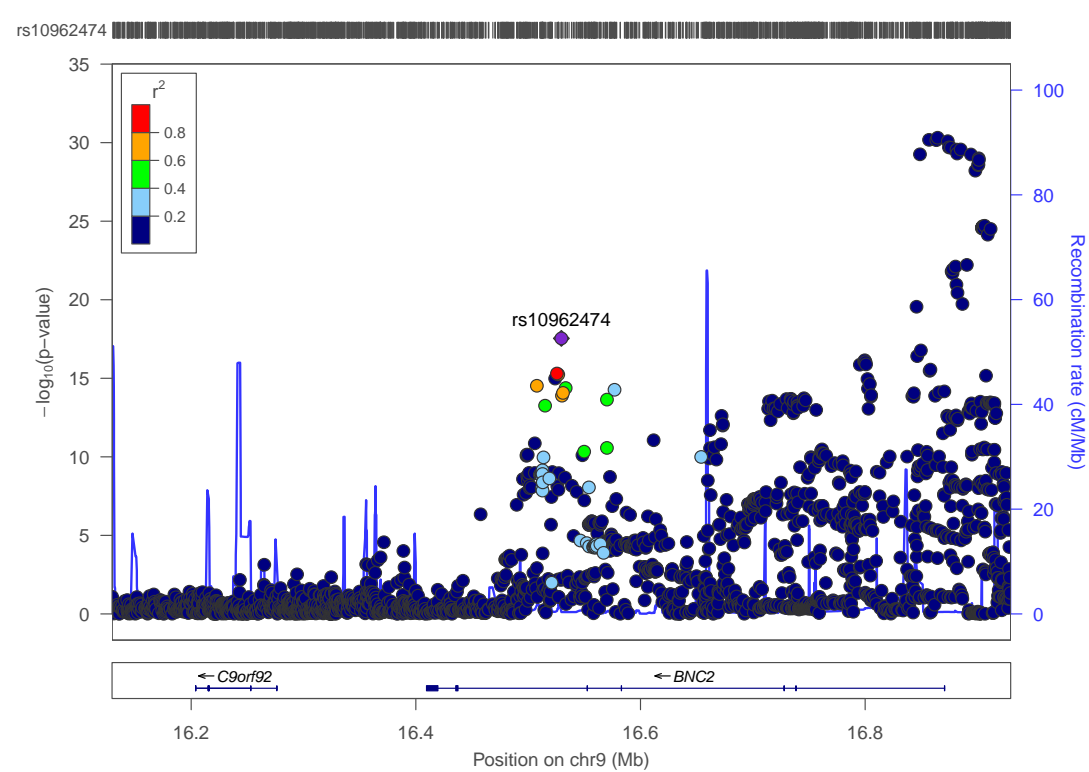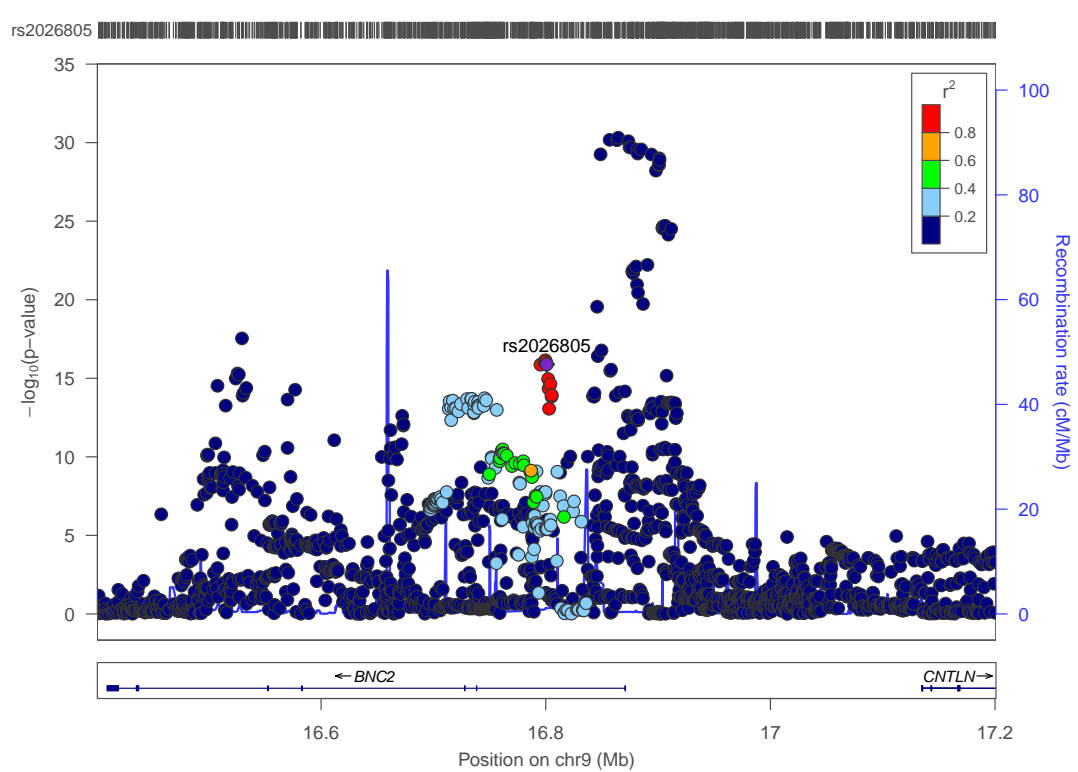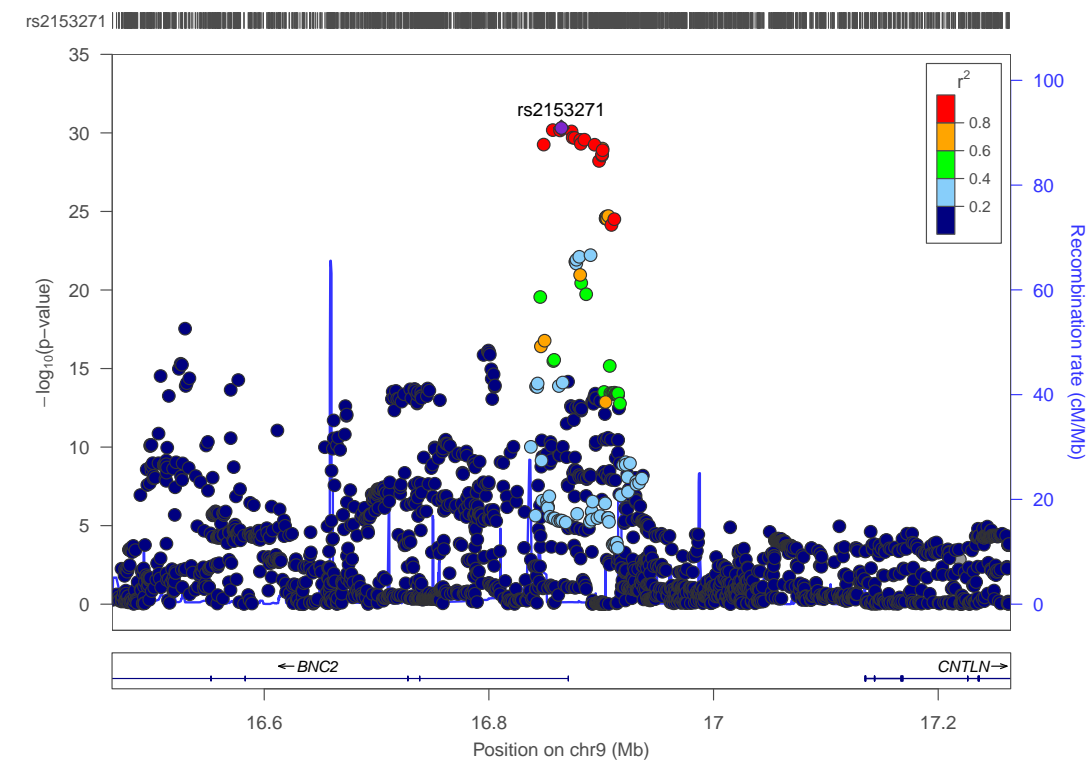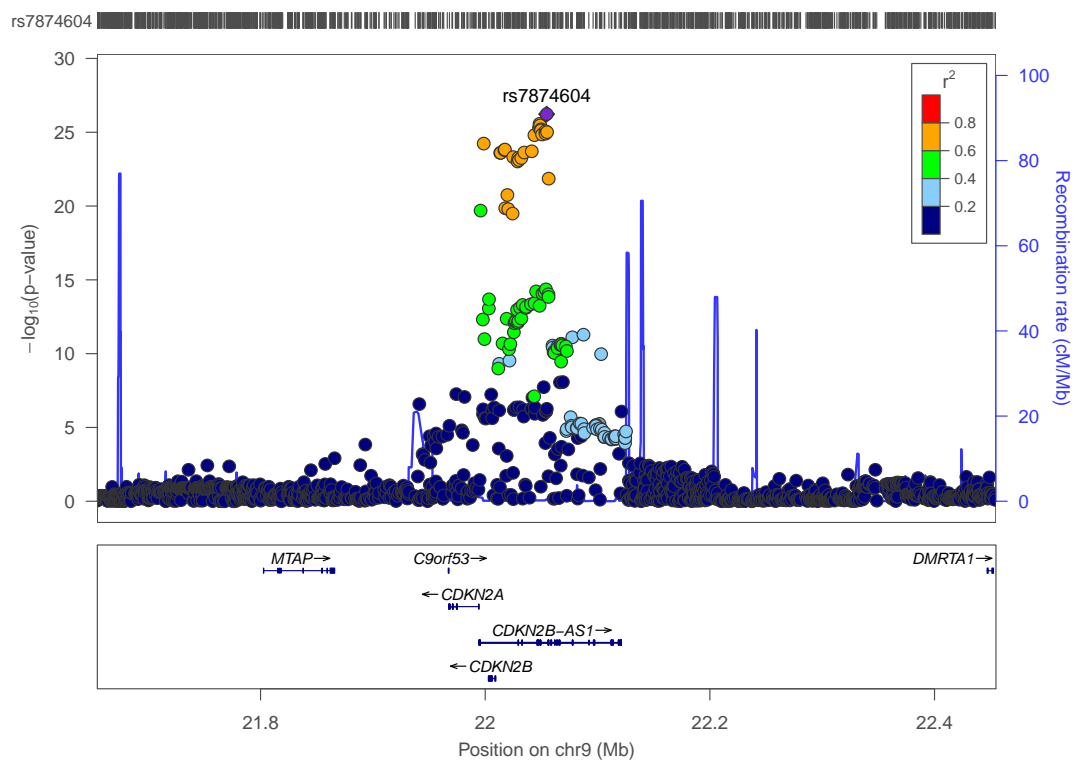

rs3739737

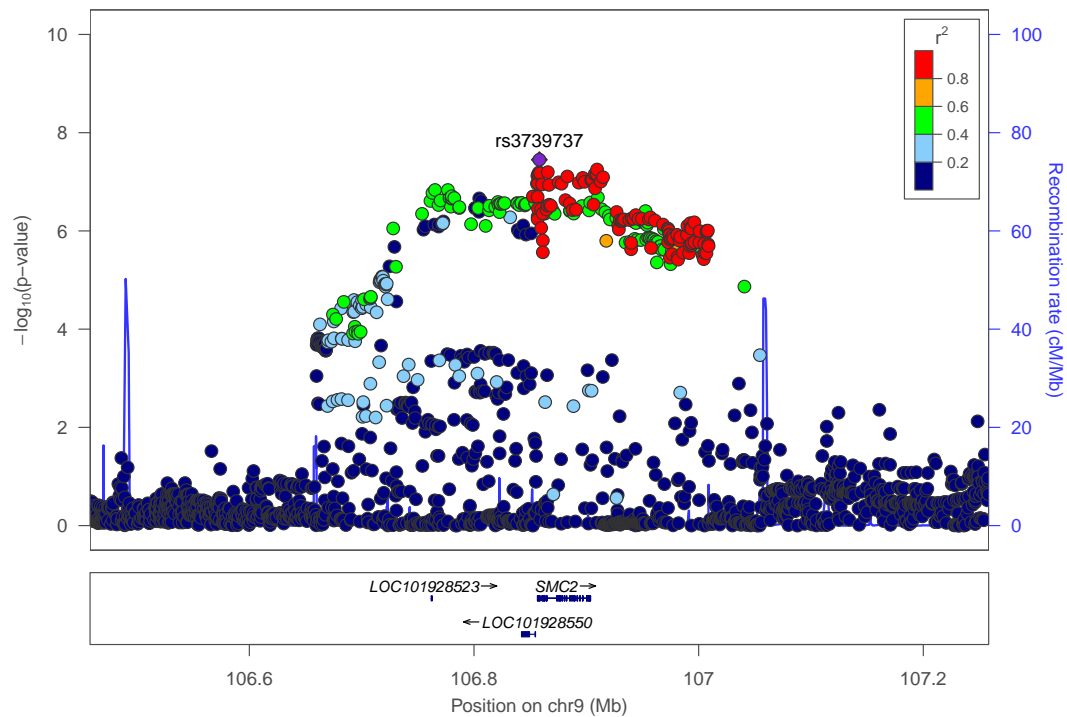

rs138501911

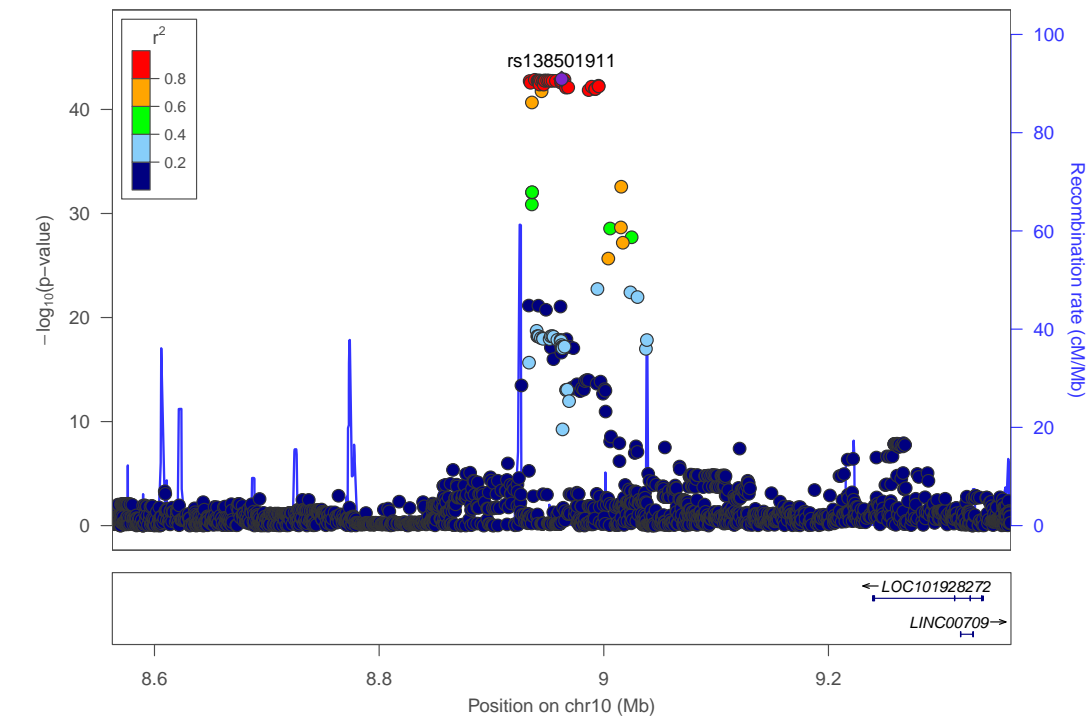

rs12767525

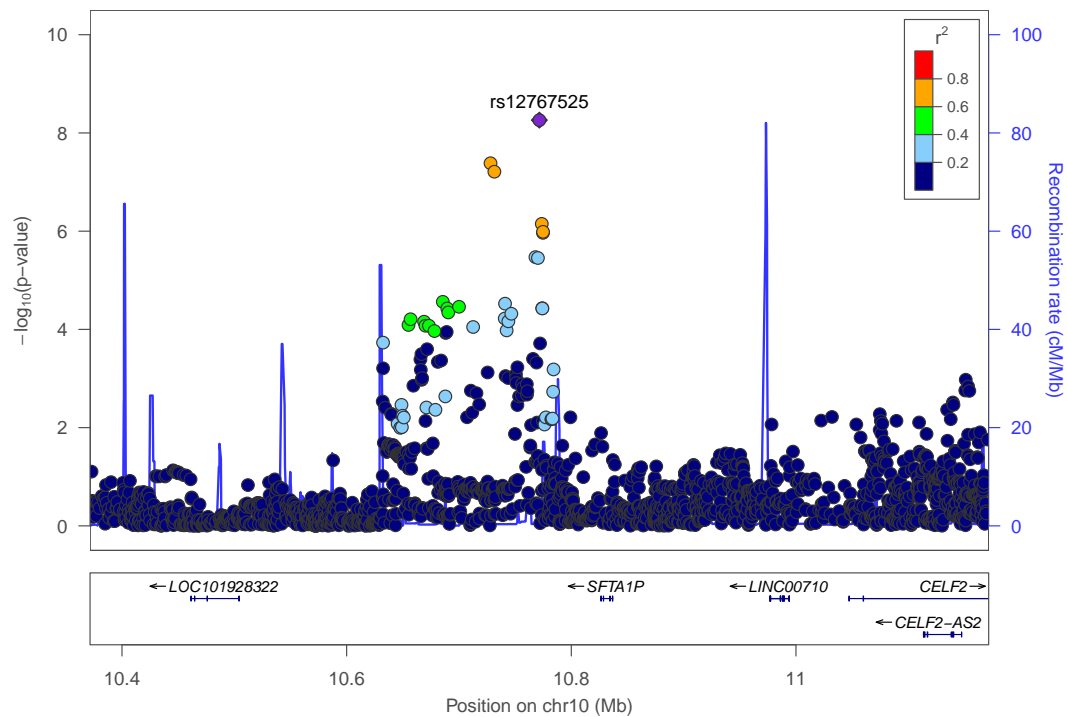

rs10995255

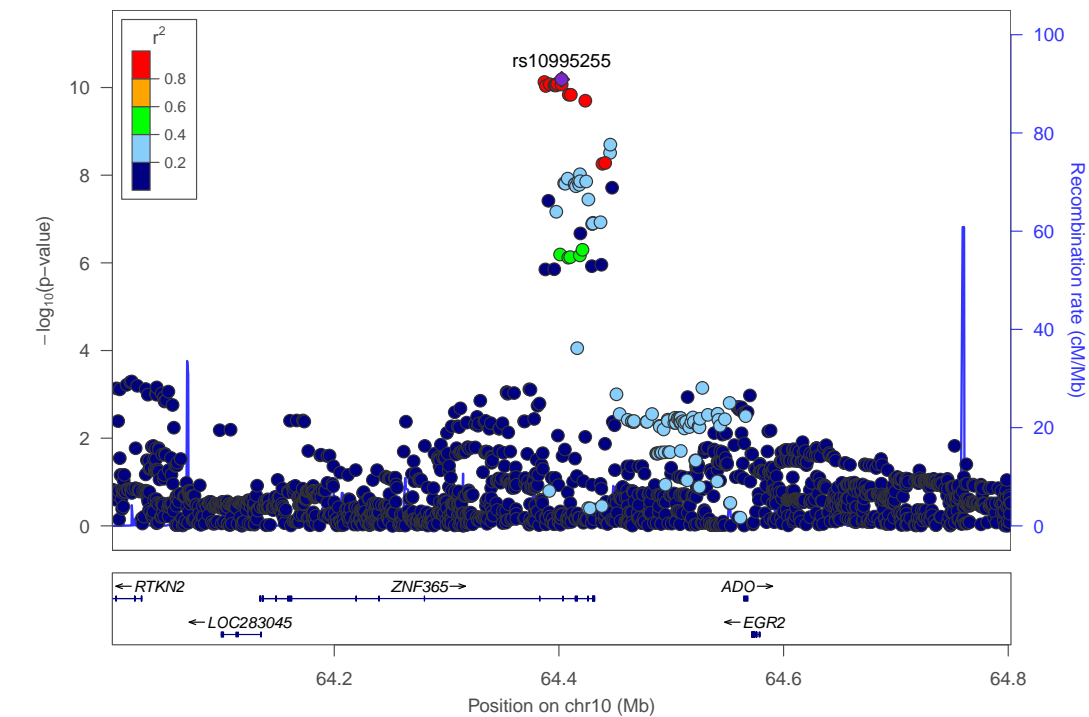

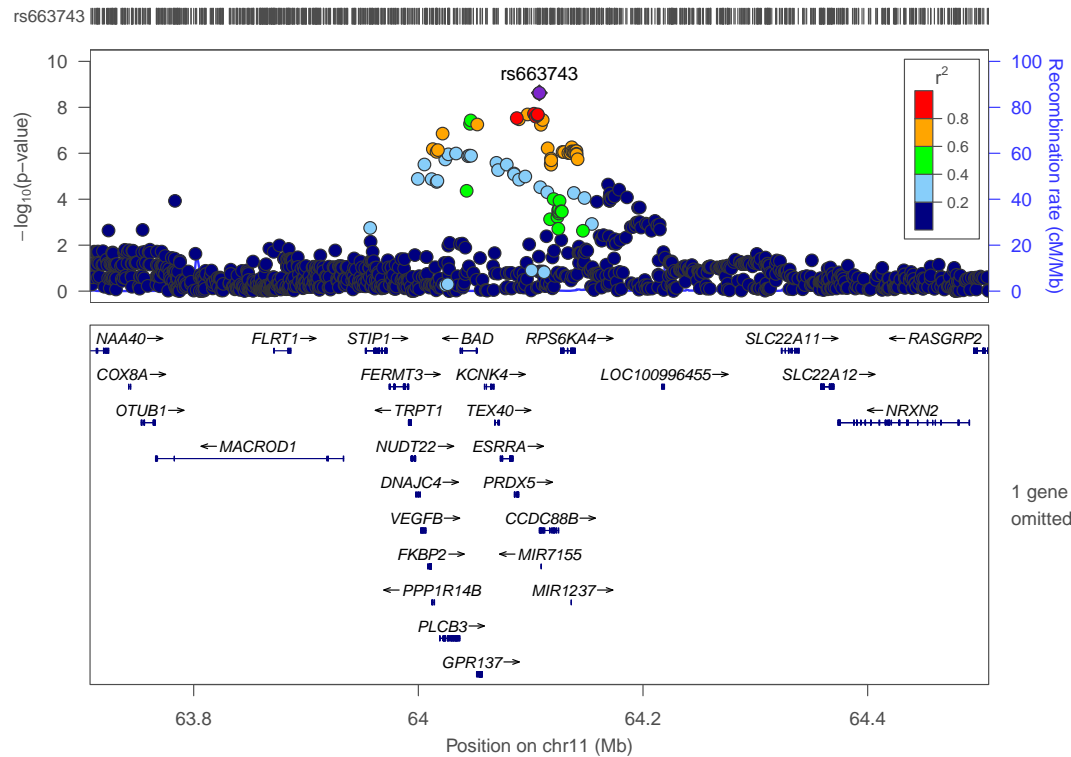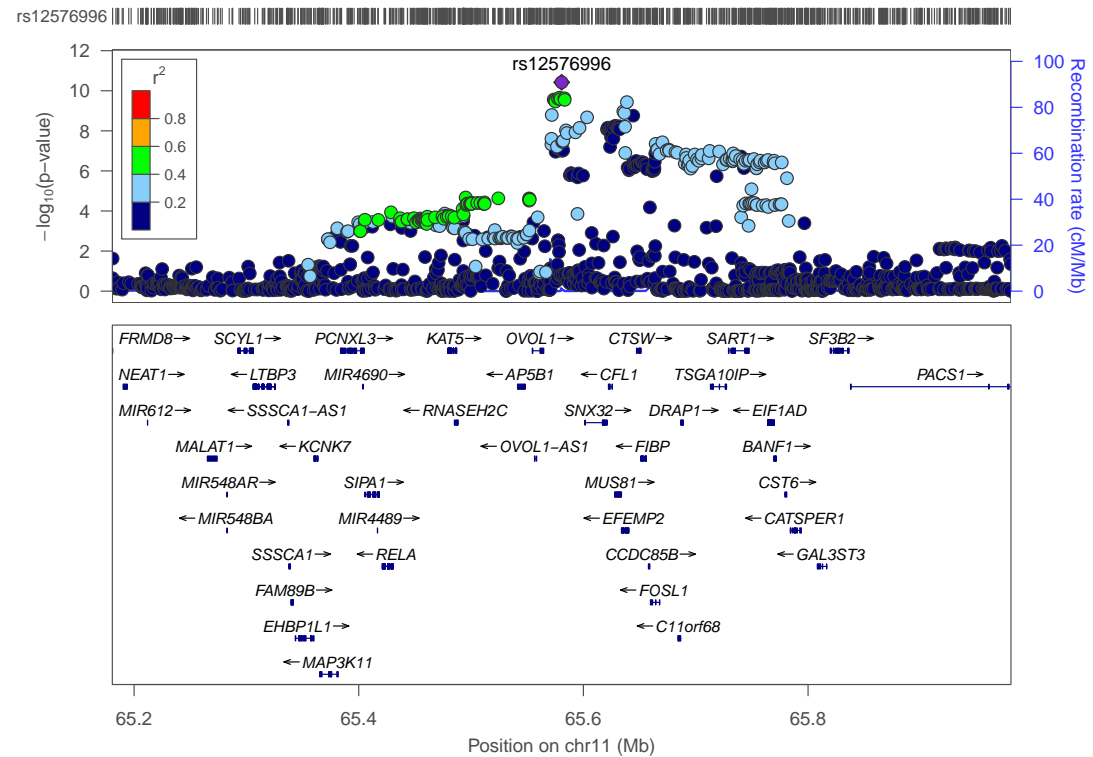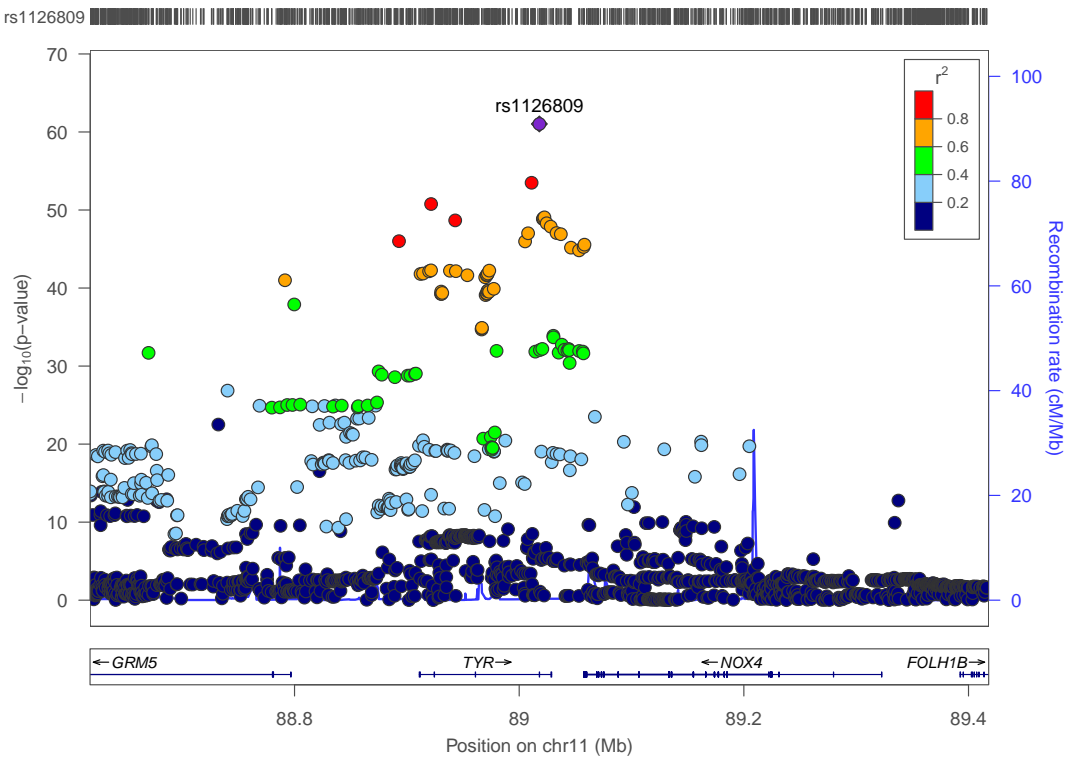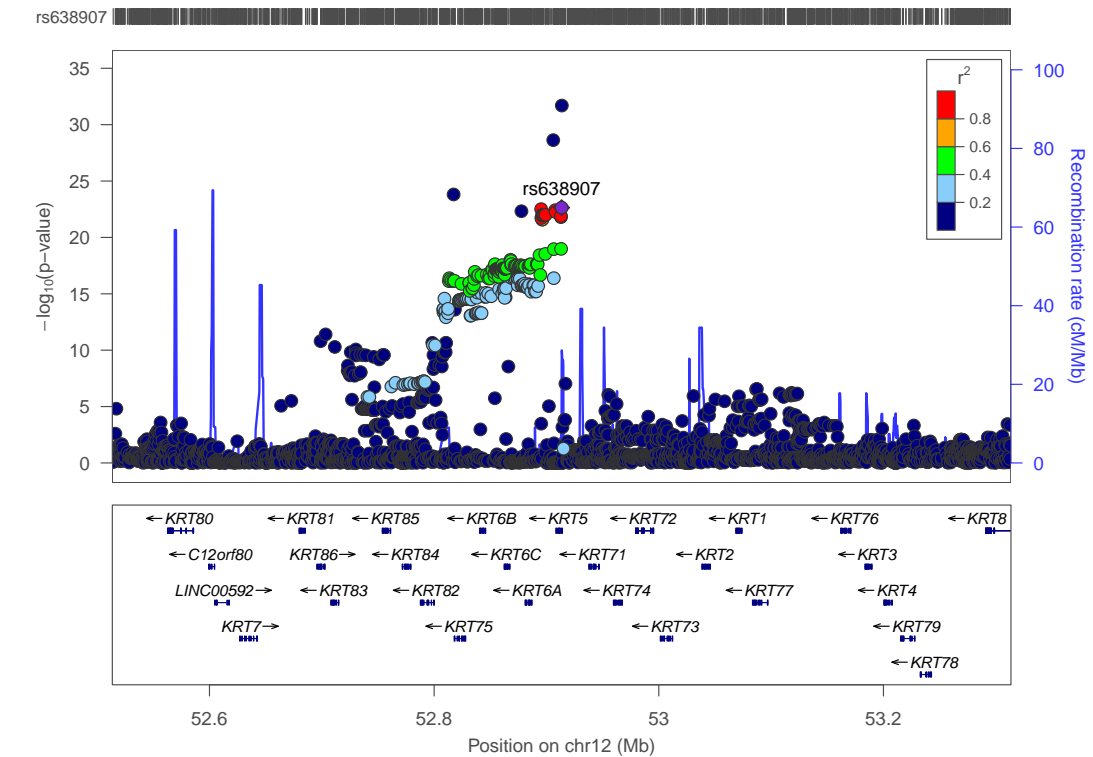

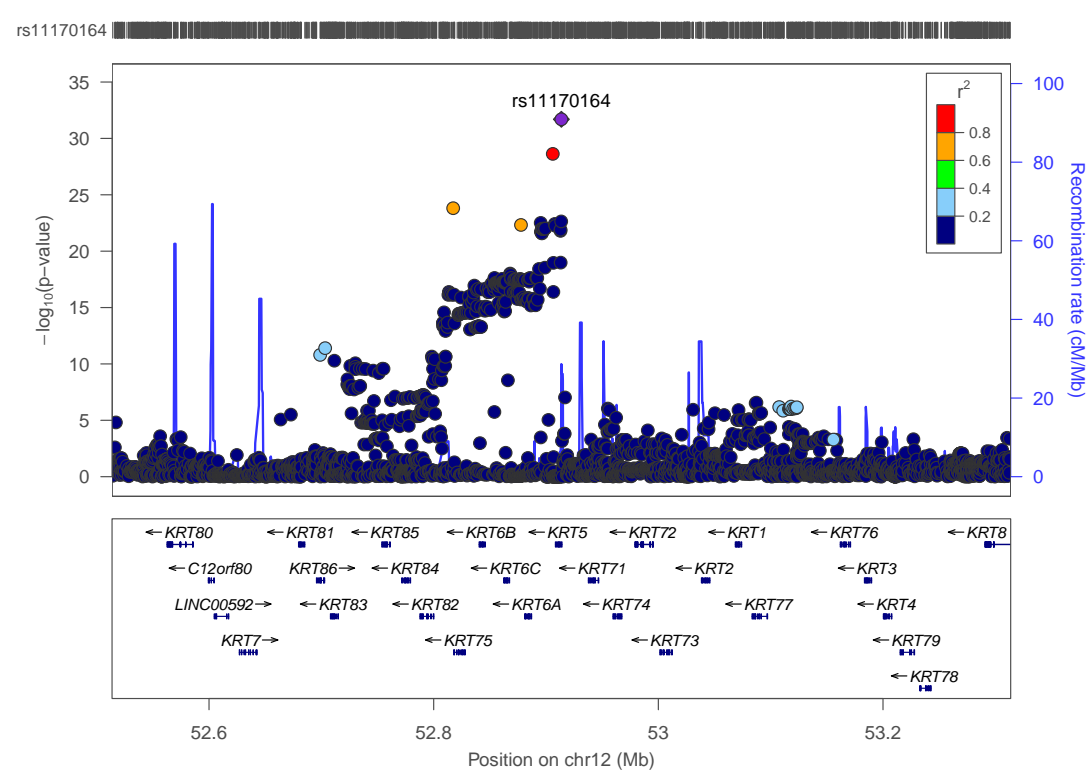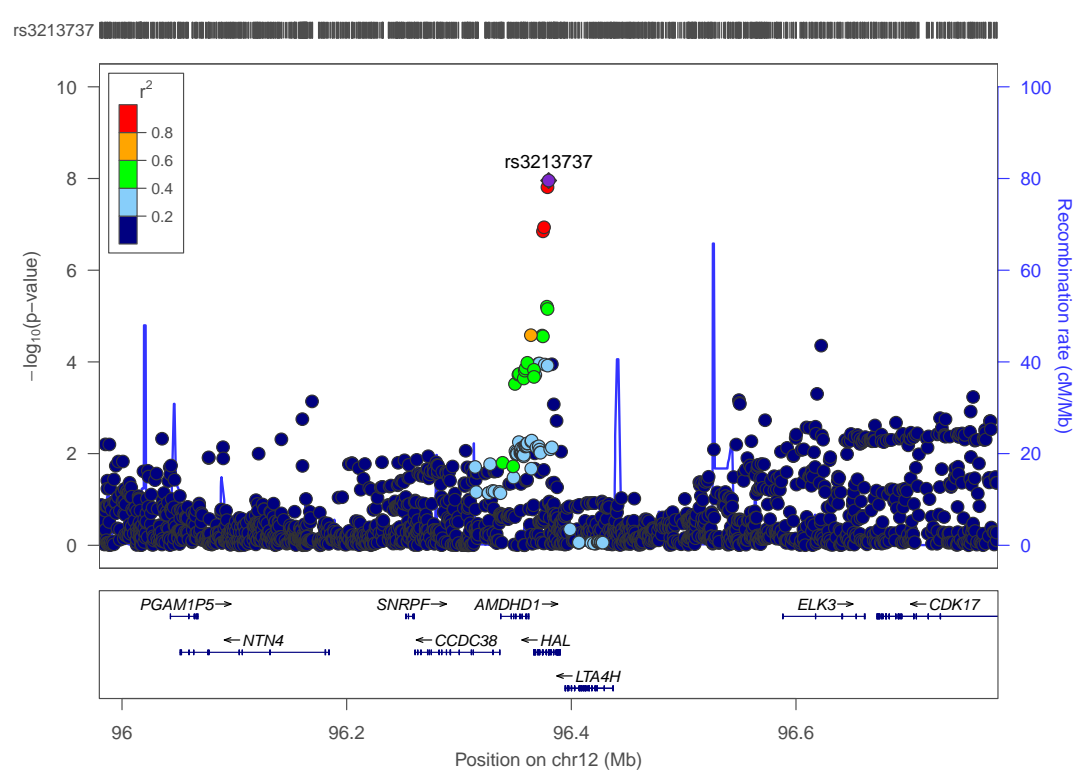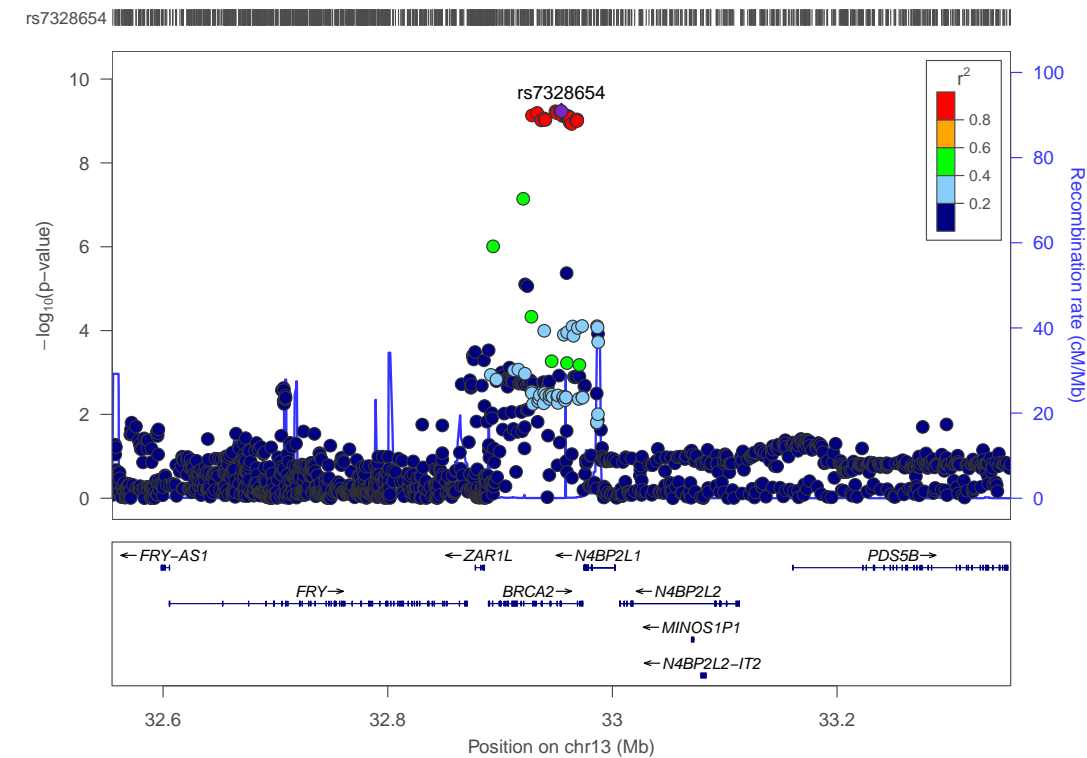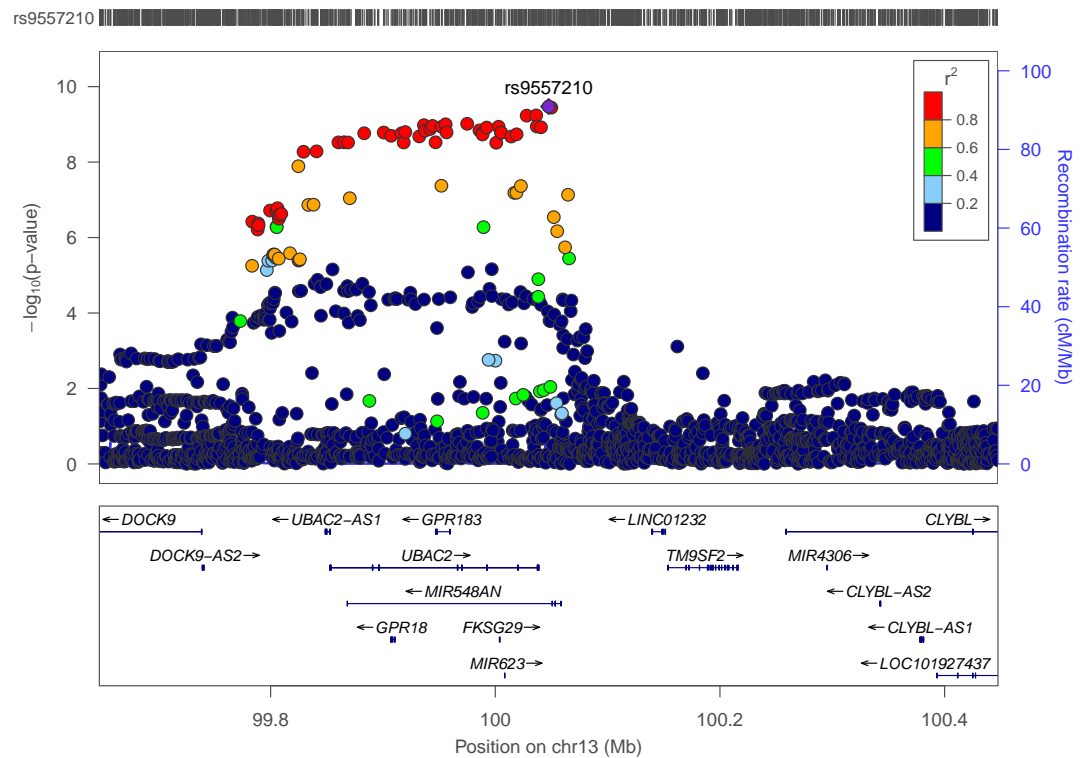

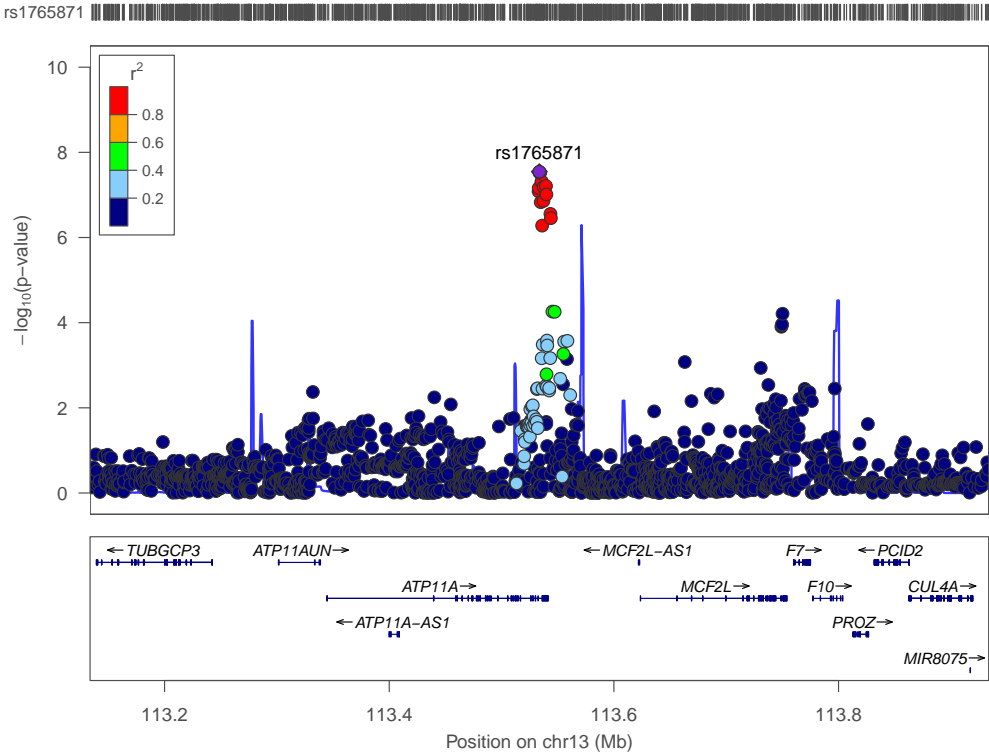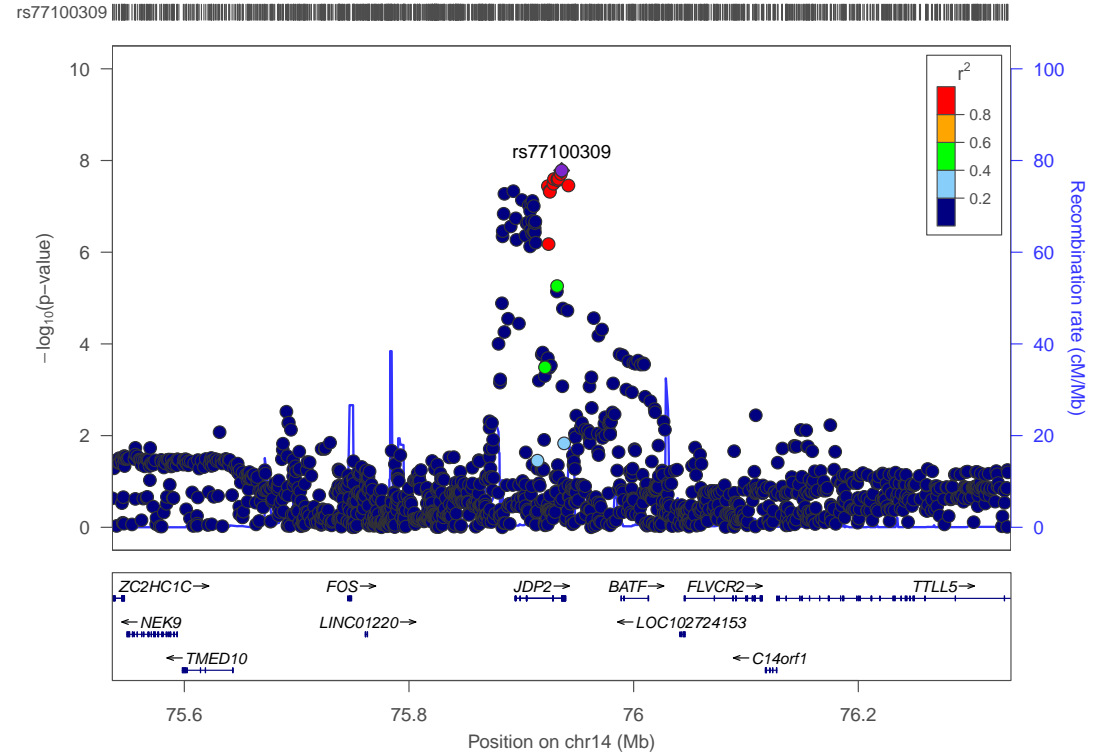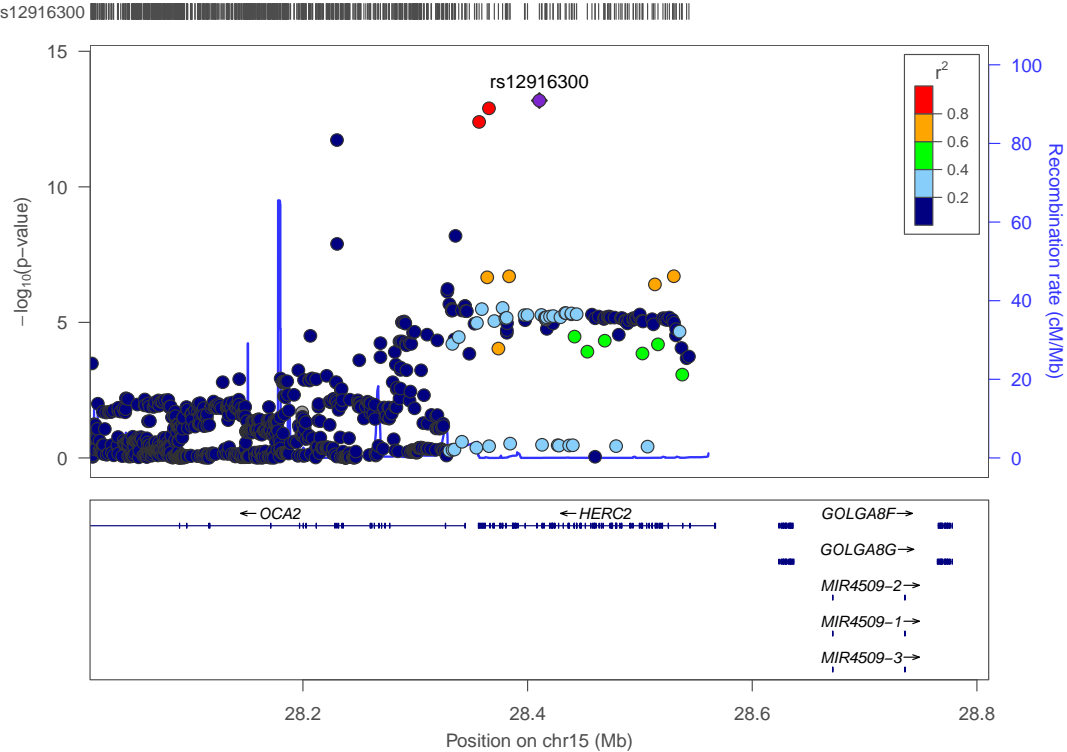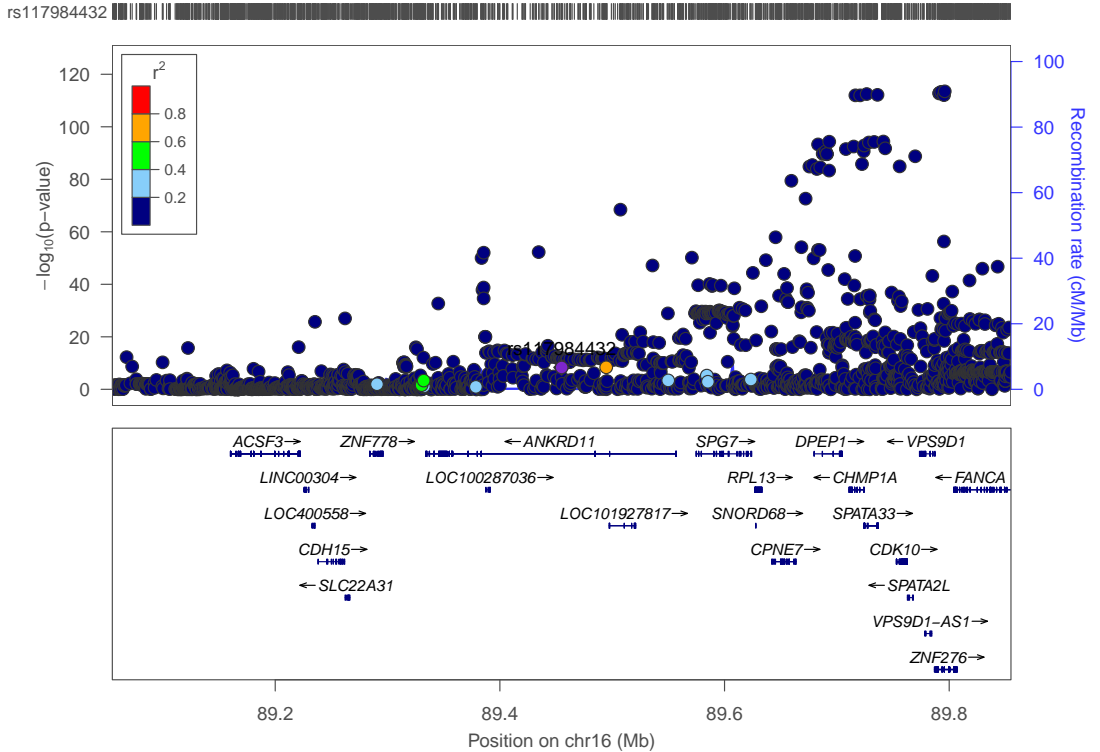

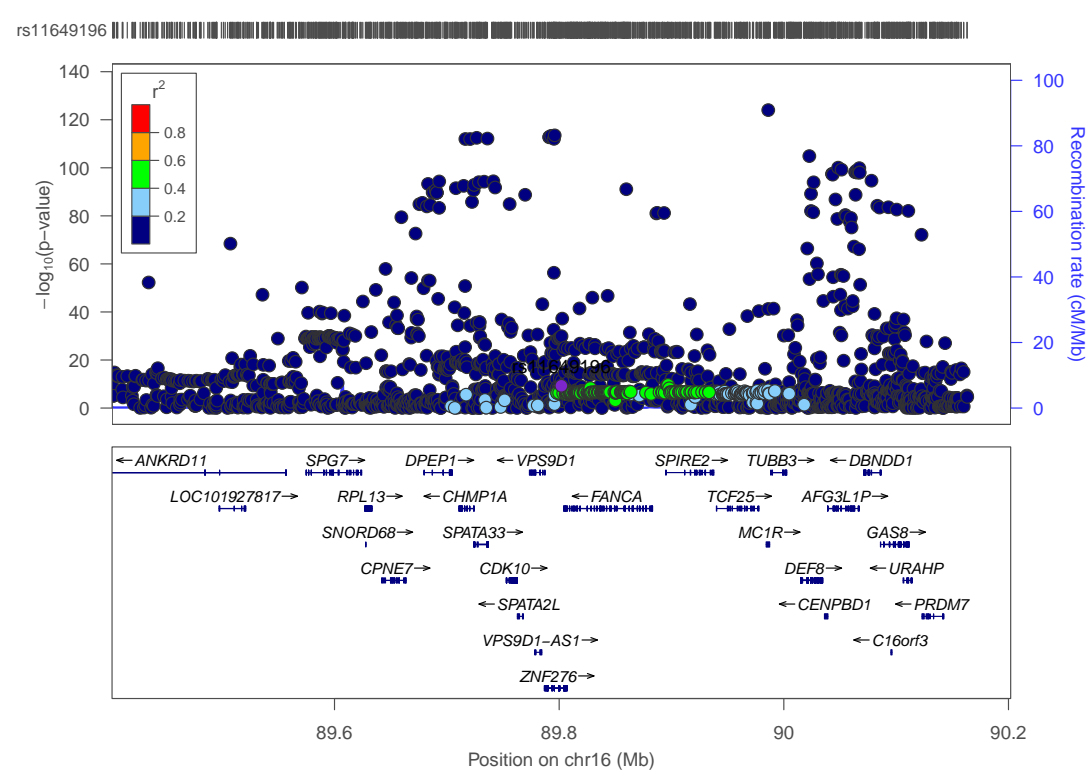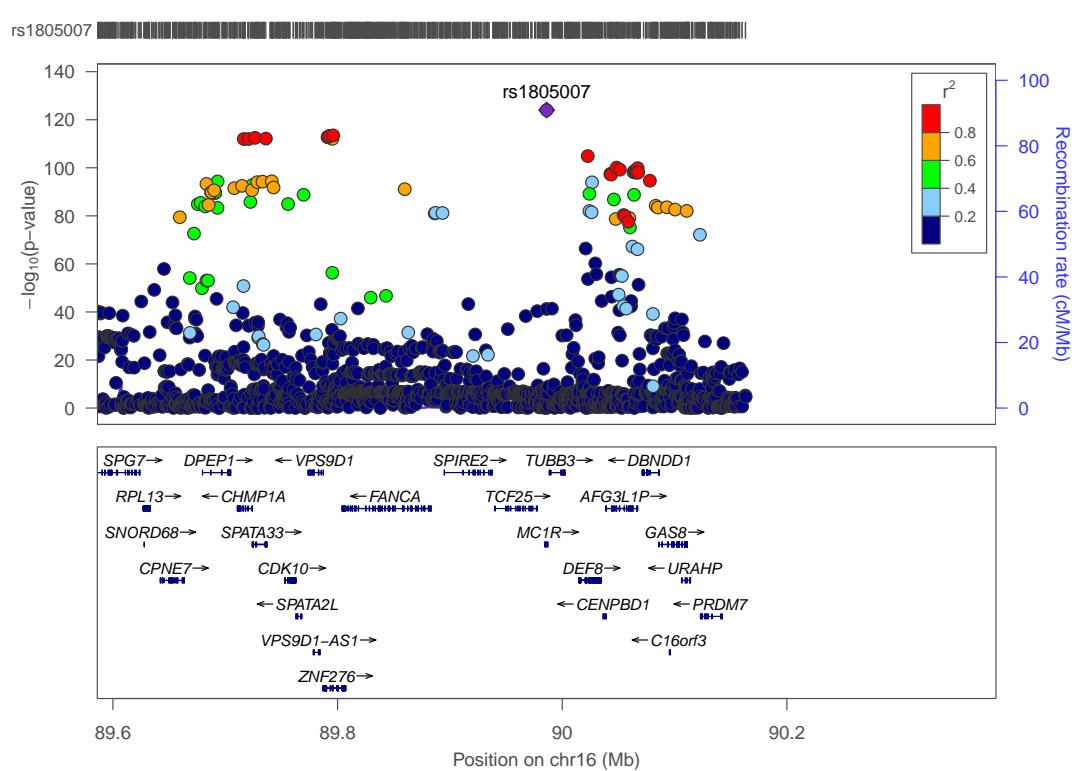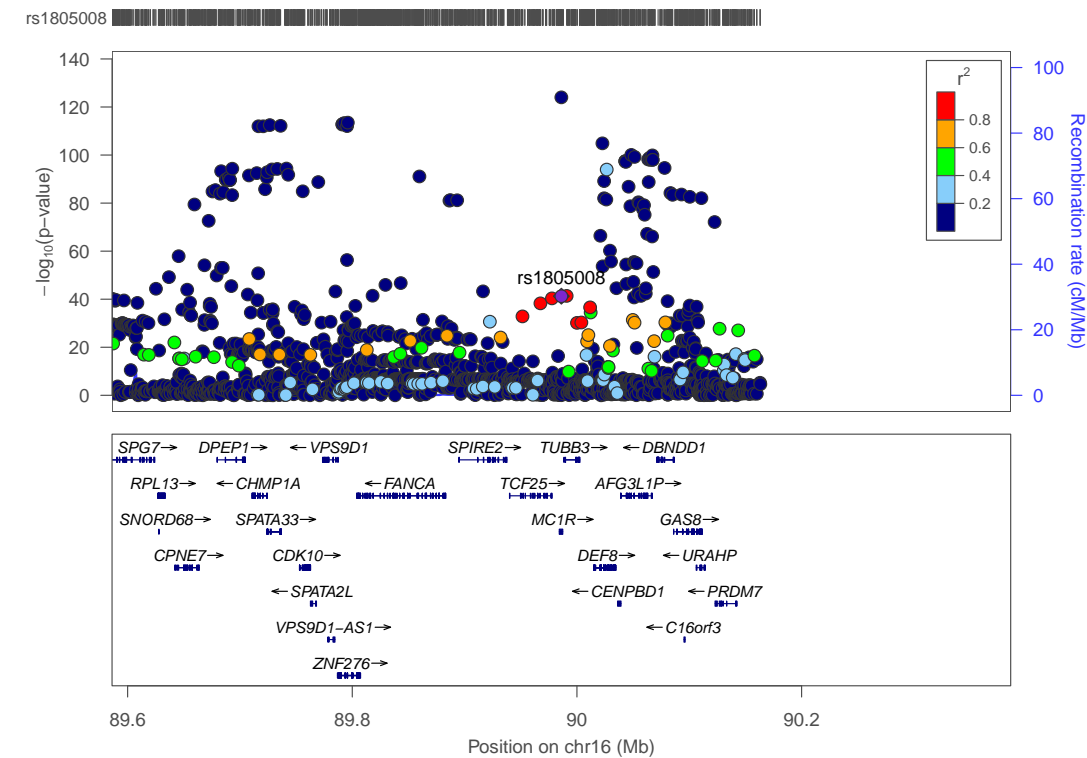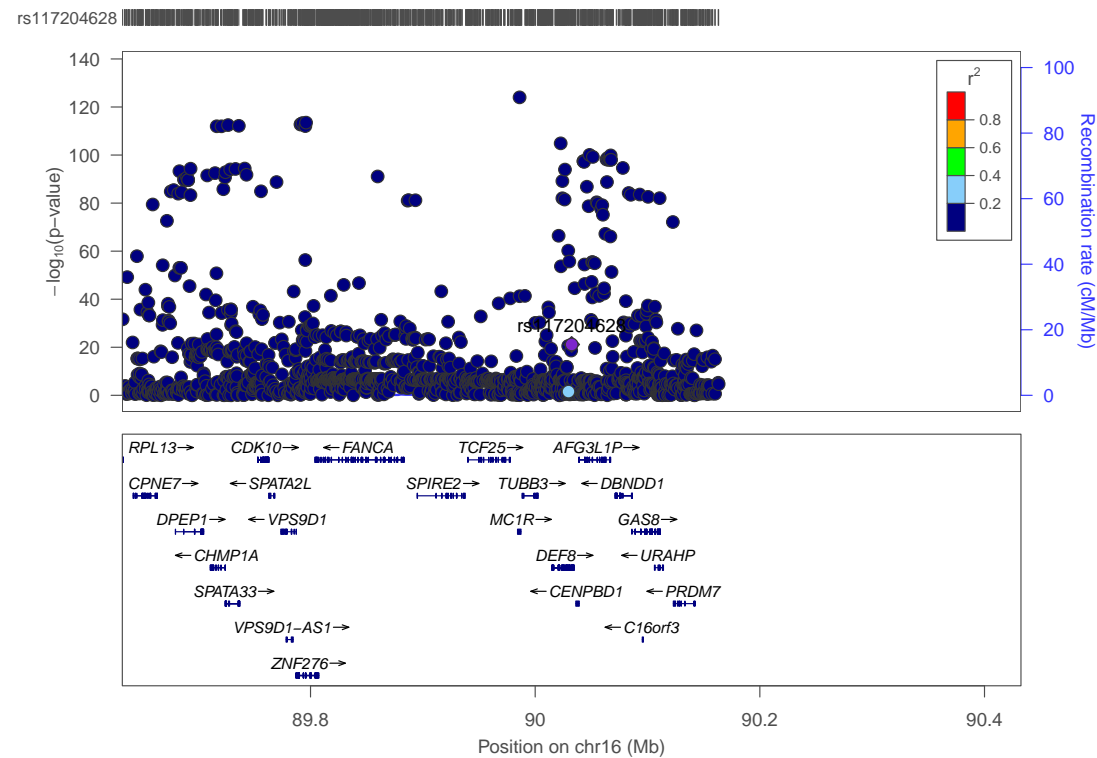

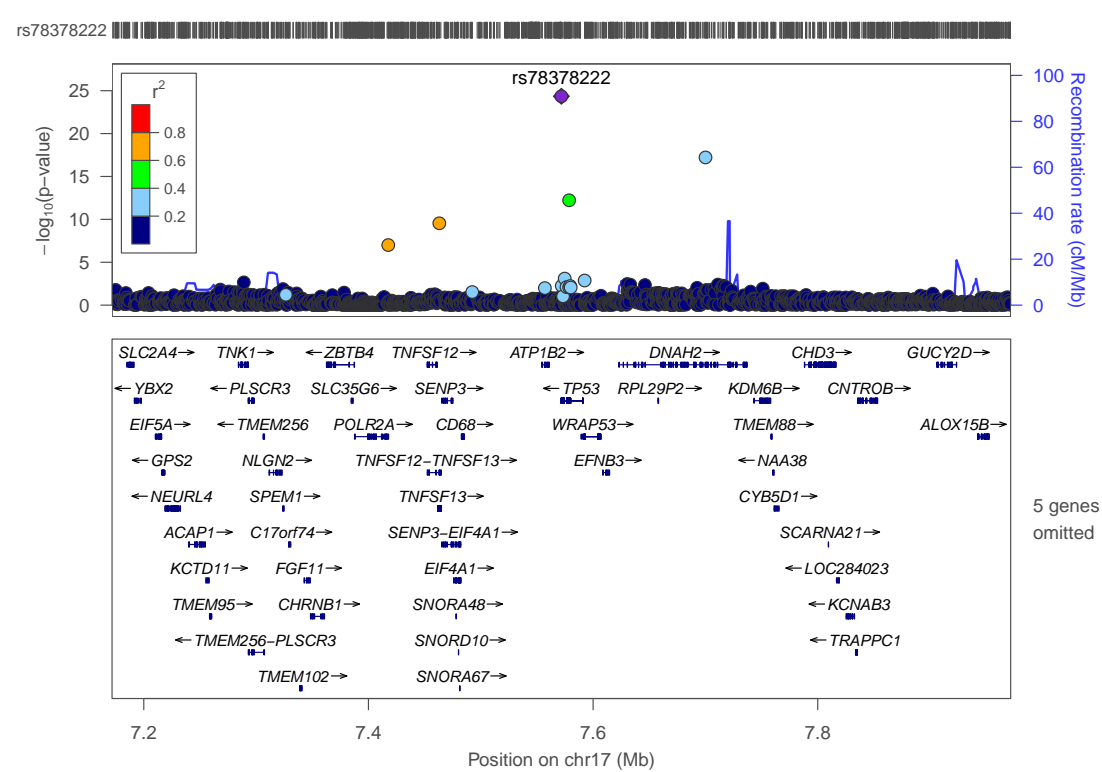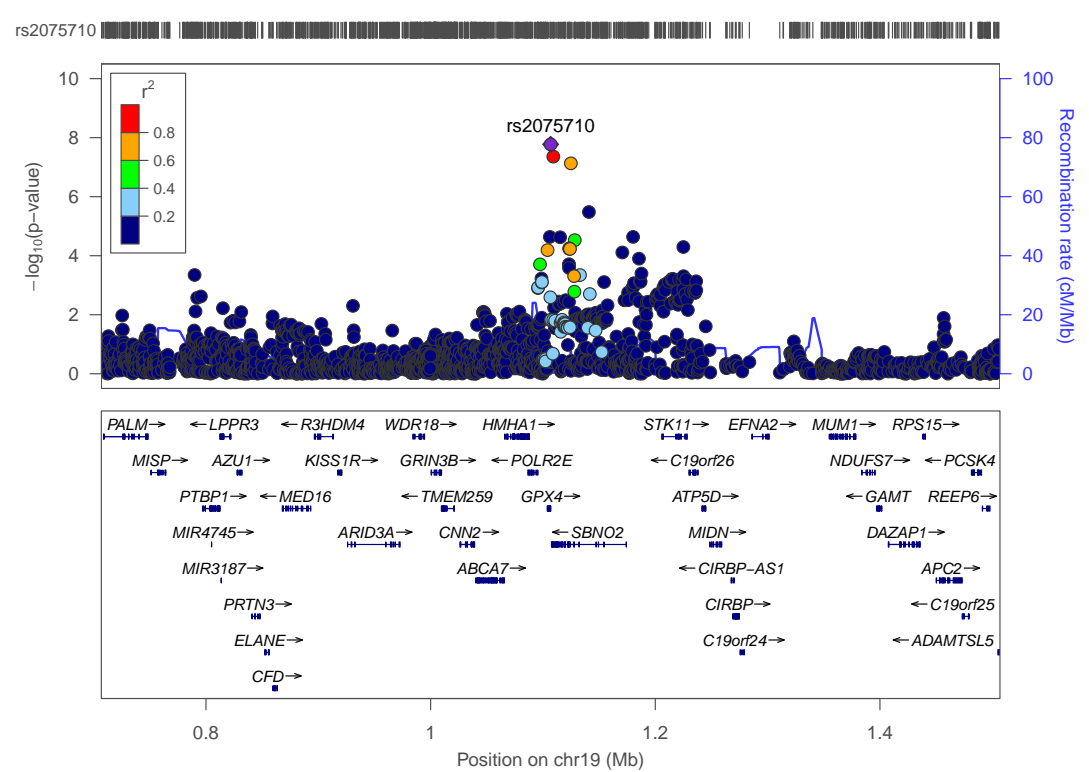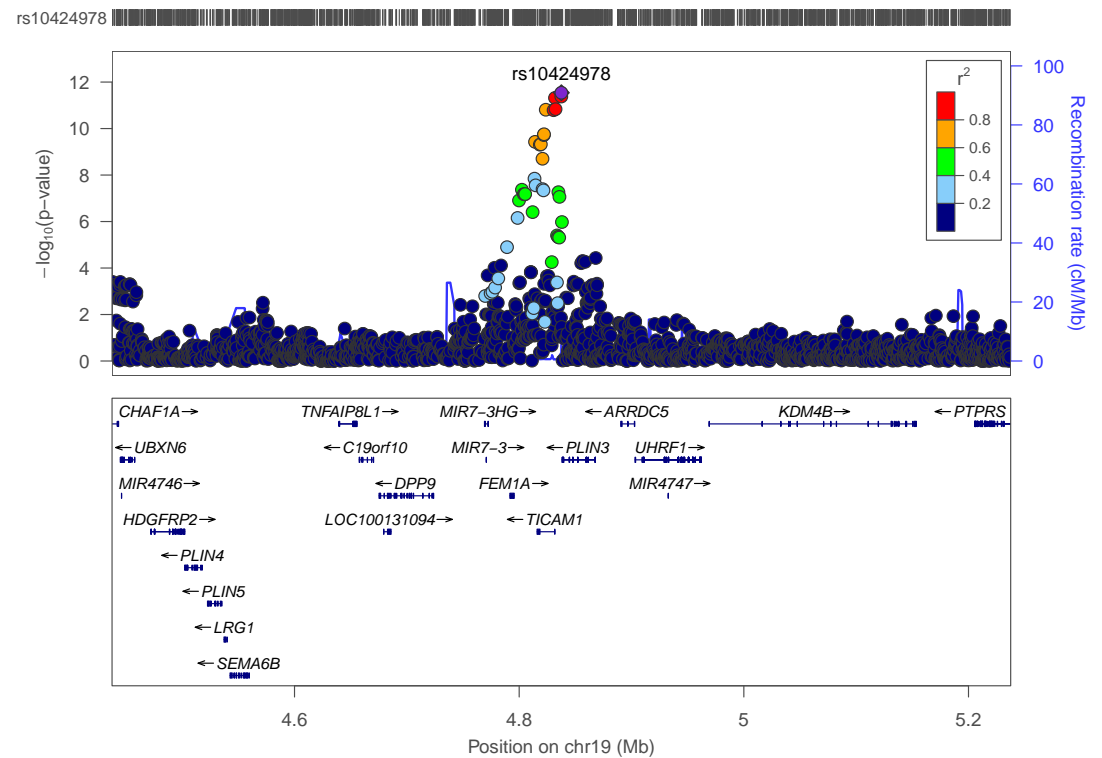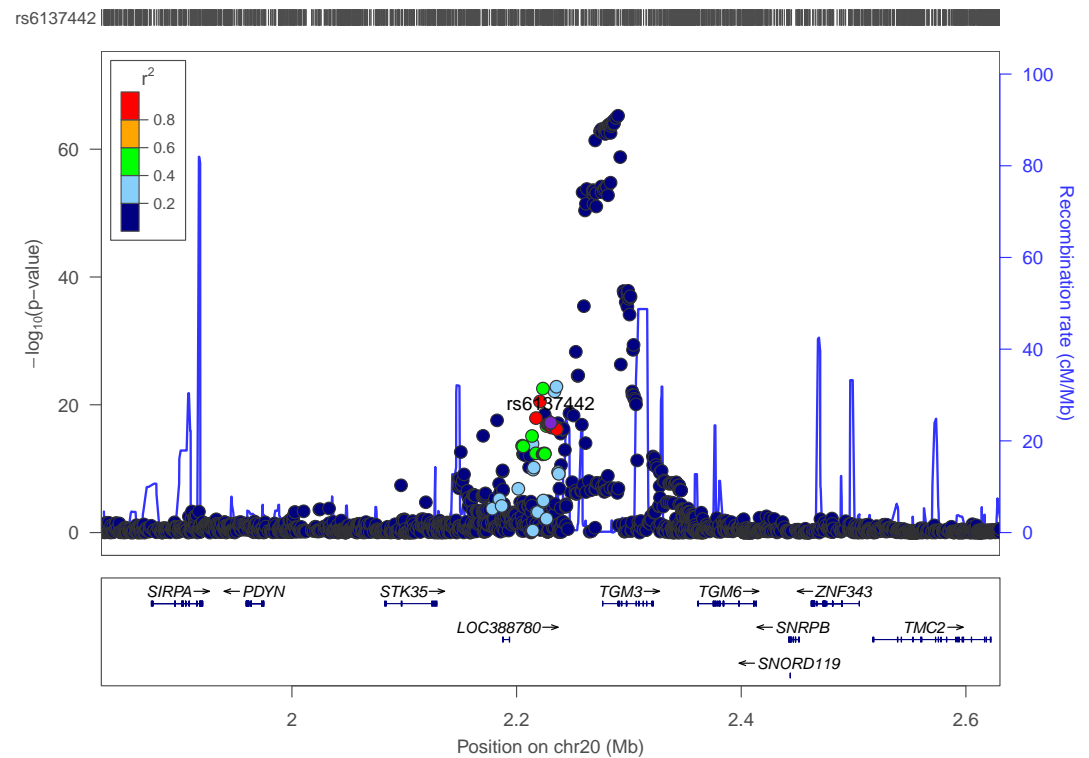

rs214803

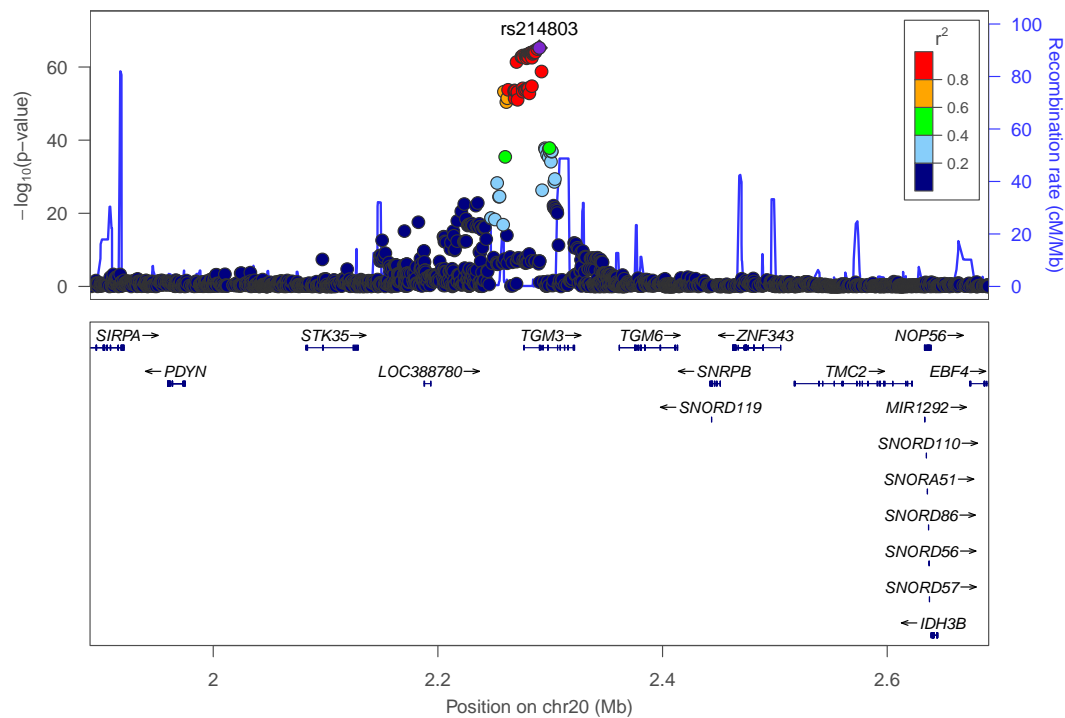

rs214831

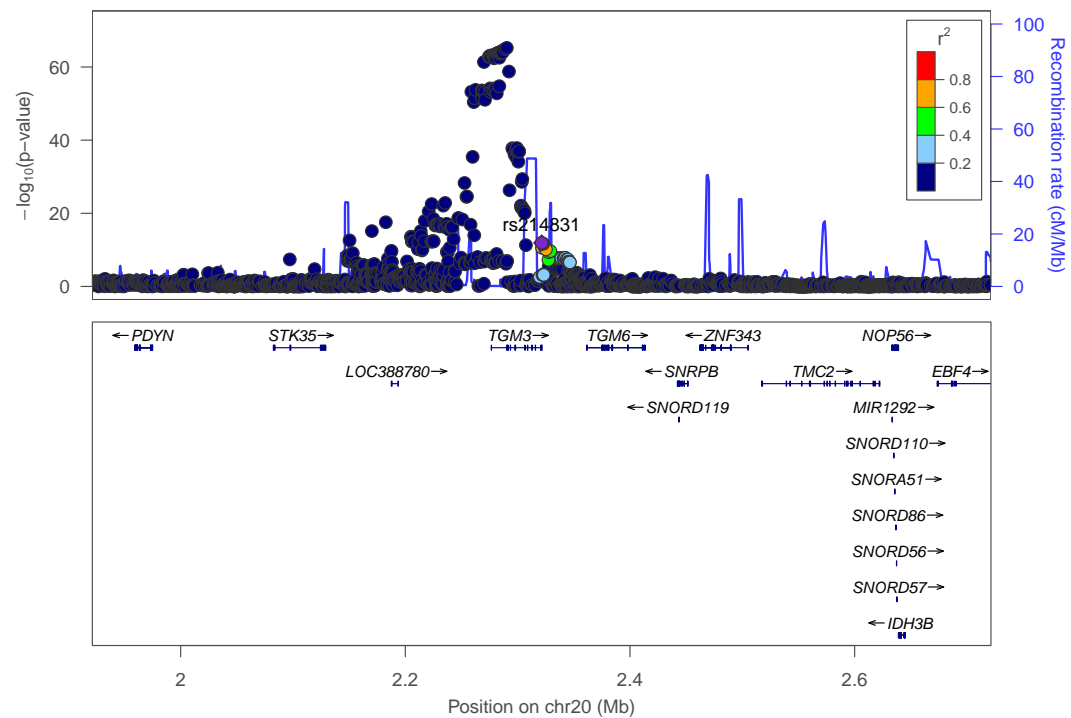

rs6059655

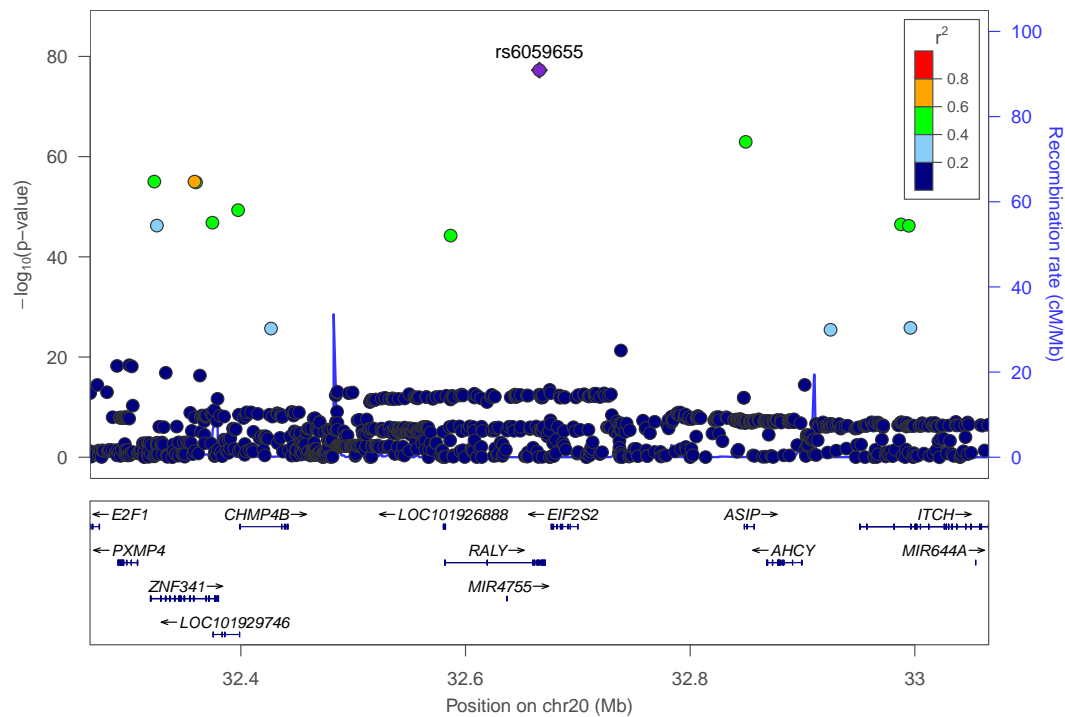

rs75653149

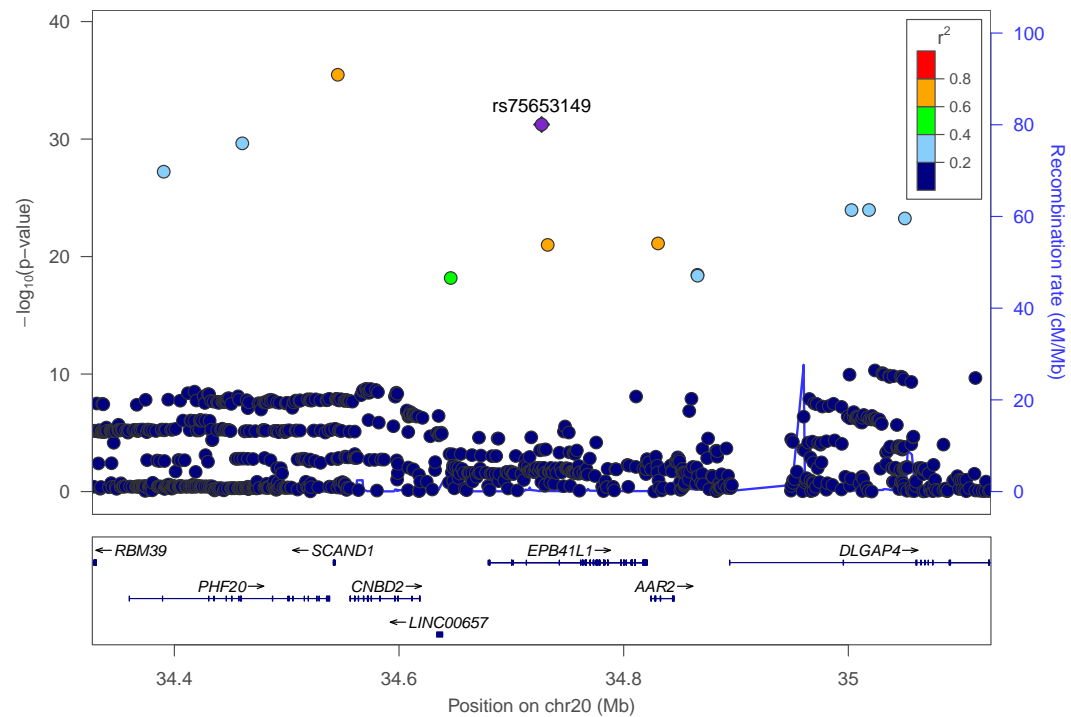

rs62202837

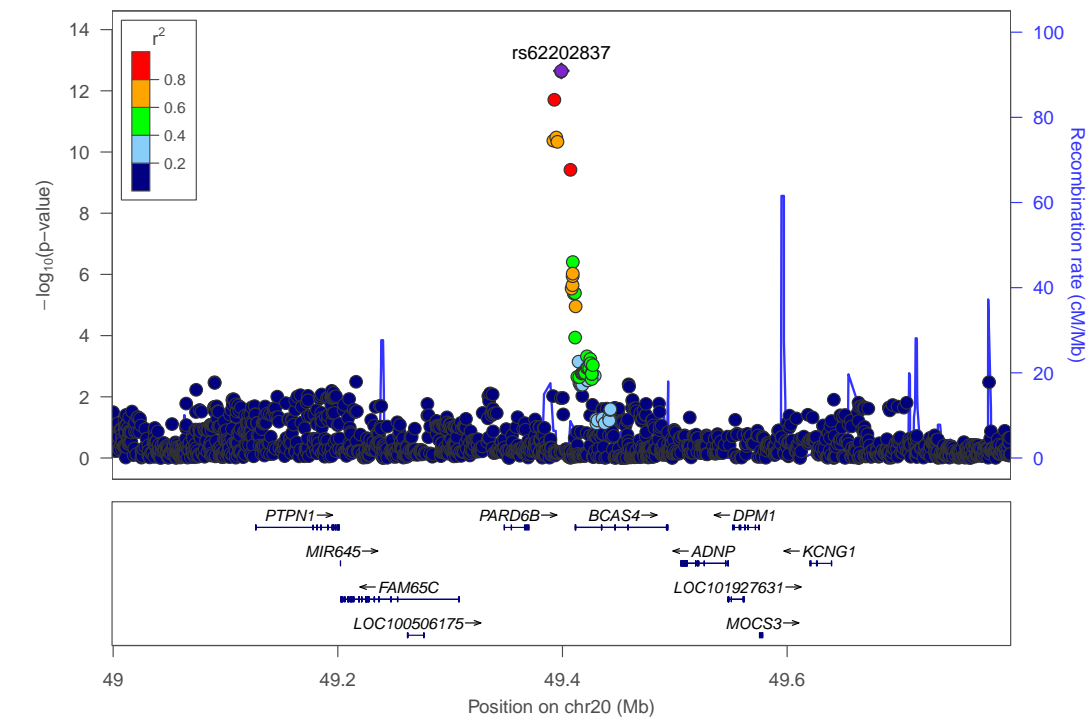

rs2776348

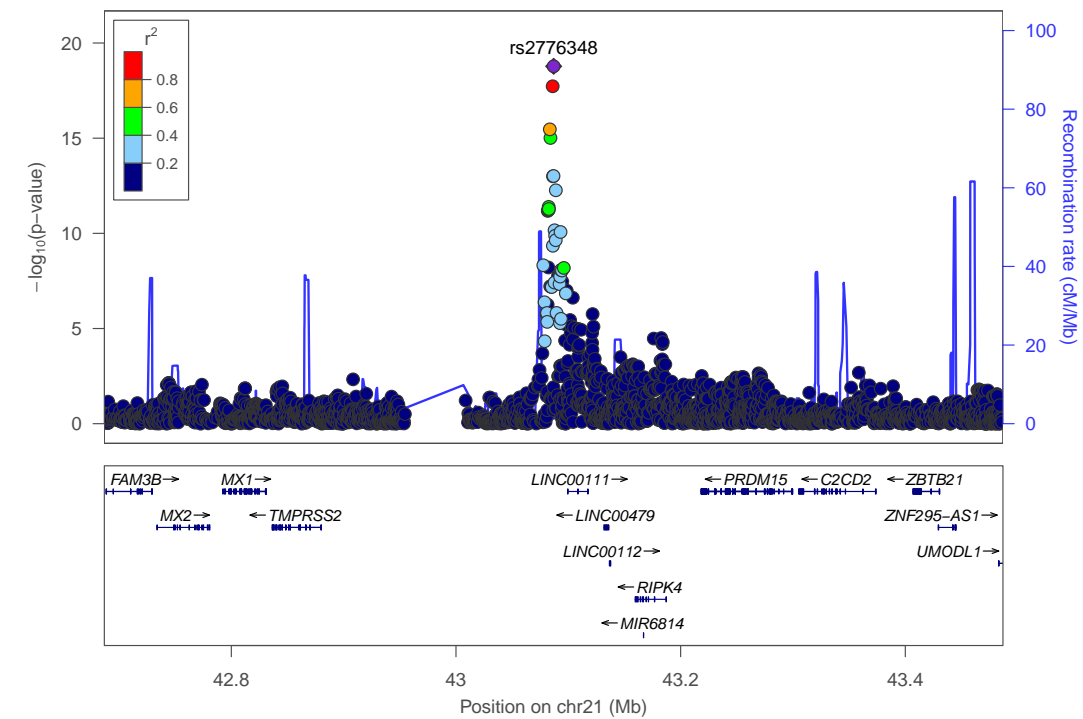

Supplement: suppl_data_ddz121 [file suppl_data_ddz121.zip › KC_regional_plot_Supplementary_Figure_8_ddz121.pdf]

rs9383064

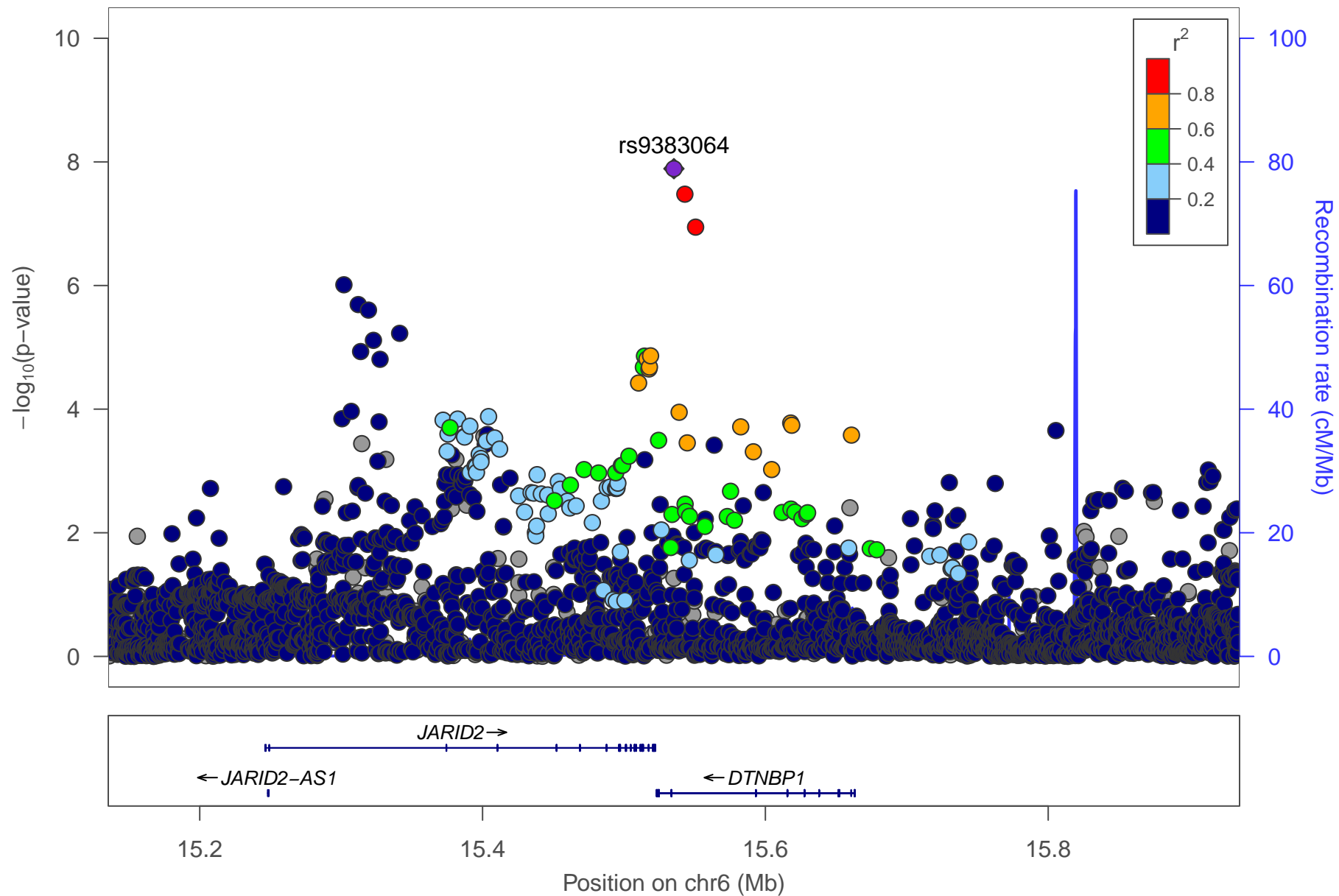

Supplement: suppl_data_ddz121 [file suppl_data_ddz121.zip › SCC_regional_plot_Supplementary_Figure_10_ddz121.pdf]
